# Therapeutic and Prophylactic Agents for Viral Diseases: Current Status, Challenges, and Future Prospects

**DOI:** 10.1002/mco2.71024

**Published:** 2026-11-09

**Authors:** Sawar Khan, Ayesha Nisar, Xiaoxu Li, Hao Yu, Abdul Qadeer, Sardar Azhar Mehmood, Imane Lamraoui, Sijie Liu, Lipeng Zhu, Xu Deng, Bo Kong, Kejun Zhong, Zanxian Xia

**Affiliations:** ^1^ Beijing Life Science Academy Beijing China; ^2^ Department of Cell Biology School of Life Sciences Central South University Changsha China; ^3^ Institute of Molecular Biology and Biotechnology The University of Lahore Lahore Pakistan; ^4^ State Key Laboratory of Genetic Evolution & Animal Models Key Laboratory of Healthy Aging Research of Yunnan Province Kunming Institute of Zoology Chinese Academy of Sciences Kunming Yunnan China; ^5^ Department of Zoology Hazara University Mansehra Pakistan; ^6^ Laboratory of Functional Ecology and Environment Faculty of Natural and Life Sciences Larbi Ben M'hidi University Oum El Bouaghi Algeria; ^7^ Xiangya School of Pharmaceutical Science Central South University Changsha China; ^8^ Hunan Key Laboratory of Animal Models For Human Diseases Hunan Key Laboratory of Medical Genetics & Center for Medical Genetics School of Life Sciences Central South University Changsha China

**Keywords:** antivirals, broad‐spectrum antivirals, CRISPR, mRNA, PrEP/PEP, vaccines

## Abstract

Viral diseases remain a major global health challenge despite substantial advances in vaccination, antiviral therapeutics, and public‐health interventions. However, current antiviral strategies are still constrained by important gaps, including rapid viral evolution, limited broad‐spectrum efficacy, immune escape, antiviral resistance, incomplete durability of protection, and inequitable global access to prevention and treatment. This review provides a comprehensive and integrated overview of contemporary antiviral prevention and therapeutic strategies, spanning vaccines, monoclonal antibodies, direct‐acting antivirals, host‐targeted therapies, immunomodulators, RNA‐based therapeutics, gene‐editing technologies, and cellular immunotherapies. We discuss the mechanistic foundations, clinical applications, pharmacologic considerations, and translational challenges associated with each modality across major human viral pathogens, including HIV, HBV, HCV, influenza, SARS‐CoV‐2, RSV, Ebola virus, arboviruses, and herpesviruses. Particular emphasis is placed on emerging innovations such as long‐acting prophylaxis, Fc‐engineered monoclonal antibodies, nanoparticle and mucosal vaccine platforms, CRISPR‐based antiviral systems, and broadly protective host‐directed approaches designed to overcome resistance and enhance pandemic preparedness. In addition, we highlight the growing importance of real‐time genomic surveillance and equitable global deployment strategies in shaping future antiviral responses, collectively underscoring that the next generation of antiviral medicine will depend on the convergence of universal immunization strategies, durable therapeutics, precision immune modulation, and rapidly deployable platform technologies capable of addressing both endemic and emerging viral threats.

## Graphical abstract

This review summarizes current therapeutic and prophylactic strategies for global viral diseases, including vaccines, PrEP, direct‐acting antivirals, and emerging modalities such as CRISPR and siRNA. It highlights their clinical impact, ongoing challenges, and future priorities toward broader protection, improved delivery, and more effective antiviral intervention.

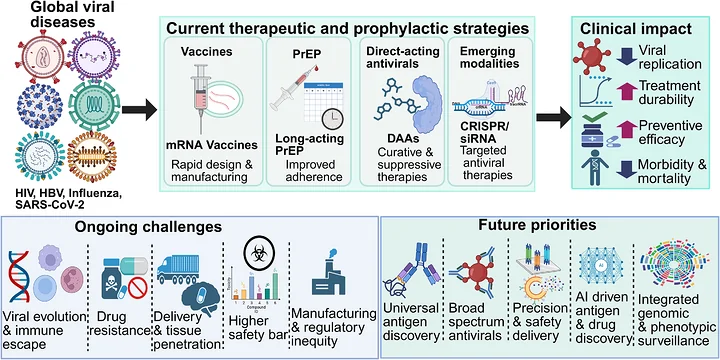

## 1 Introduction

Viral infections remain a leading contributor to global morbidity and mortality. For instance, an estimated 40 million people were living with human immunodeficiency virus (HIV) in 2021, and HIV‐related deaths numbered roughly 0.72 million that year [1]. Seasonal influenza causes an estimated 0.291–0.646 million respiratory deaths worldwide each year [2]. Viral hepatitis also imposes a heavy toll: despite declining rates, hepatitis B and C still contribute hundreds of thousands of deaths and millions of disability‐adjusted life years (DALYs) from cirrhosis and liver cancer annually [3]. Respiratory syncytial virus (RSV) in children under 5 years of age causes on the order of 0.1 million deaths per year [4], almost all in low‐ and middle‐income countries. Dengue infects hundreds of millions of people annually (about 390 million infections per year, of which ∼96 million are clinically apparent) [5], leading to tens of thousands of hospitalizations and thousands of deaths in endemic regions. Emerging zoonotic viruses underscore the unpredictability of viral threats—for example, SARS‐CoV‐2 caused a global pandemic with many millions of cases and deaths, and Ebola virus outbreaks (most recently in Central Africa) continue to cause significant mortality. Notably, the burden of these viral diseases is highly inequitable: low‑ and middle‑income regions bear the greatest share. For instance, influenza mortality and RSV deaths are concentrated in sub‑Saharan Africa and Southeast Asia [2, 4], and disparities in viral hepatitis DALYs reflect unequal access to prevention and care [3].

Current prophylactic and therapeutic strategies have achieved important successes but also face limitations. Vaccination is the cornerstone of viral disease prevention: for example, universal infant HBV vaccination has dramatically reduced new HBV infections, and multivalent influenza vaccines reduce seasonal flu burden (although antigenic drift limits efficacy). Rapidly developed COVID‐19 vaccines (notably mRNA and viral‑vector platforms) exemplify recent advances, but effective vaccines remain lacking for viruses such as HIV, hepatitis C, and dengue (or have limited use, as with the dengue vaccine). Antiviral drugs have transformed management of some infections: combination antiretroviral therapy (ART) can suppress HIV replication and greatly extend survival (though a cure remains elusive), and direct‐acting antivirals (DAAs) now cure the vast majority of hepatitis C virus (HCV) infections [3]. However, long‐term treatment is required, resistance can develop, and access is uneven. Monoclonal antibody (mAb) therapies have also made an impact: for instance, the RSV monoclonal palivizumab used prophylactically in high‐risk infants reduces RSV hospitalizations to only a few percent [6], and human monoclonals (MAb114 and REGN‐EB3) tested in Ebola virus disease significantly improved survival compared with standard care [7]. Immune‐modulating agents (e.g., corticosteroids and interleukin‐6 inhibitors) proved lifesaving in severe COVID‐19; and host‐directed strategies (such as enhancing innate immunity) are under investigation. Yet each approach has caveats: vaccines can be outpaced by viral mutation; antivirals often target only a single virus; mAbs and biologics are expensive and may lose efficacy with viral variants; and nonspecific immunomodulators can have systemic side effects. Though vaccines, antiviral drugs, monoclonals, and immunotherapies have curbed many viral diseases; gaps remain—especially for emerging or neglected viruses—and no single modality is universally effective.

Given this dynamic landscape, a comprehensive, up‐to‐date review of viral prophylaxis and therapy is timely. The COVID‐19 pandemic has accelerated research and platform technologies (e.g. mRNA vaccines, rapid monoclonal development), but it has also highlighted the continuing challenges of viral evolution and access disparities. We therefore synthesize recent advances and persistent challenges in antiviral strategies, aiming to inform clinicians, virologists, and policymakers. Such a synthesis will help guide optimal use of current tools and prioritize future research in the post‐COVID era of rapid biomedical innovation.

## 2 Mechanisms of Viral Pathogenesis

Viral infection proceeds through a coordinated sequence of attachment and entry, genome replication and protein processing, virion assembly and release, and dissemination within susceptible tissues. Each stage exposes molecular vulnerabilities that can be exploited by preventive or therapeutic interventions. Viral receptors, fusion proteins, host proteases, polymerases, reverse transcriptases, viral proteases, and assembly machinery constitute major targets for neutralizing antibodies (nAbs), entry inhibitors, and DAAs [8, 9, 10]. Conversely, antigenic variation, glycan shielding, interference with antigen presentation, latency, cell‐to‐cell transmission, and the selection of drug‐resistant variants can reduce the effectiveness of vaccines, antibodies, and antiviral agents [10]. This section therefore focuses on the mechanistic features of viral infection that directly inform intervention design, including viral entry, replication and protein processing, immune recognition and escape, antiviral resistance, and the influence of tissue tropism and viral reservoirs on treatment efficacy.

### 2.1 Viral Entry and Early Events

Viral infection begins with attachment to specific host‐cell receptors, a major determinant of cellular and tissue tropism. Enveloped viruses subsequently fuse with the plasma or endosomal membrane, whereas nonenveloped viruses penetrate or disrupt cellular membranes to release their genomes [11]. Influenza A virus, for example, uses hemagglutinin (HA) to bind cell‐surface sialic acids and enter through receptor‐mediated endocytosis (Figure 1); endosomal acidification then triggers membrane fusion and release of viral ribonucleoproteins, which are transported to the nucleus [11, 12]. SARS‐CoV‐2 spike binds angiotensin‐converting enzyme 2 (ACE2) and is activated by TMPRSS2 at the cell surface or cathepsins within endosomes [13]. Flaviviruses (such as dengue virus) also enter via endocytosis, followed by low‐pH‐induced rearrangement of its envelope protein and membrane fusion [14]. In contrast to these endocytic pathways, some enveloped viruses fuse directly at the plasma membrane. A notable example is HIV‐1: the HIV envelope glycoprotein (Env) binds to the CD4 receptor on T cells and then to a coreceptor (CCR5 or CXCR4), which induces a conformational change in Env that exposes the fusion peptide and merges the viral and cellular membranes [15]. This pH‐independent fusion at the cell surface allows HIV to inject its capsid into the cytoplasm. Notably, the same Env‐mediated fusion can cause an infected cell to fuse with neighboring uninfected cells, forming multinucleated syncytia—a process that contributes to cell‐to‐cell spread. Overall, viral entry strategies vary across families, but all serve to deliver the viral genome across the protective barrier of the cell membrane and initiate the next phase of infection [16].

**FIGURE 1:**
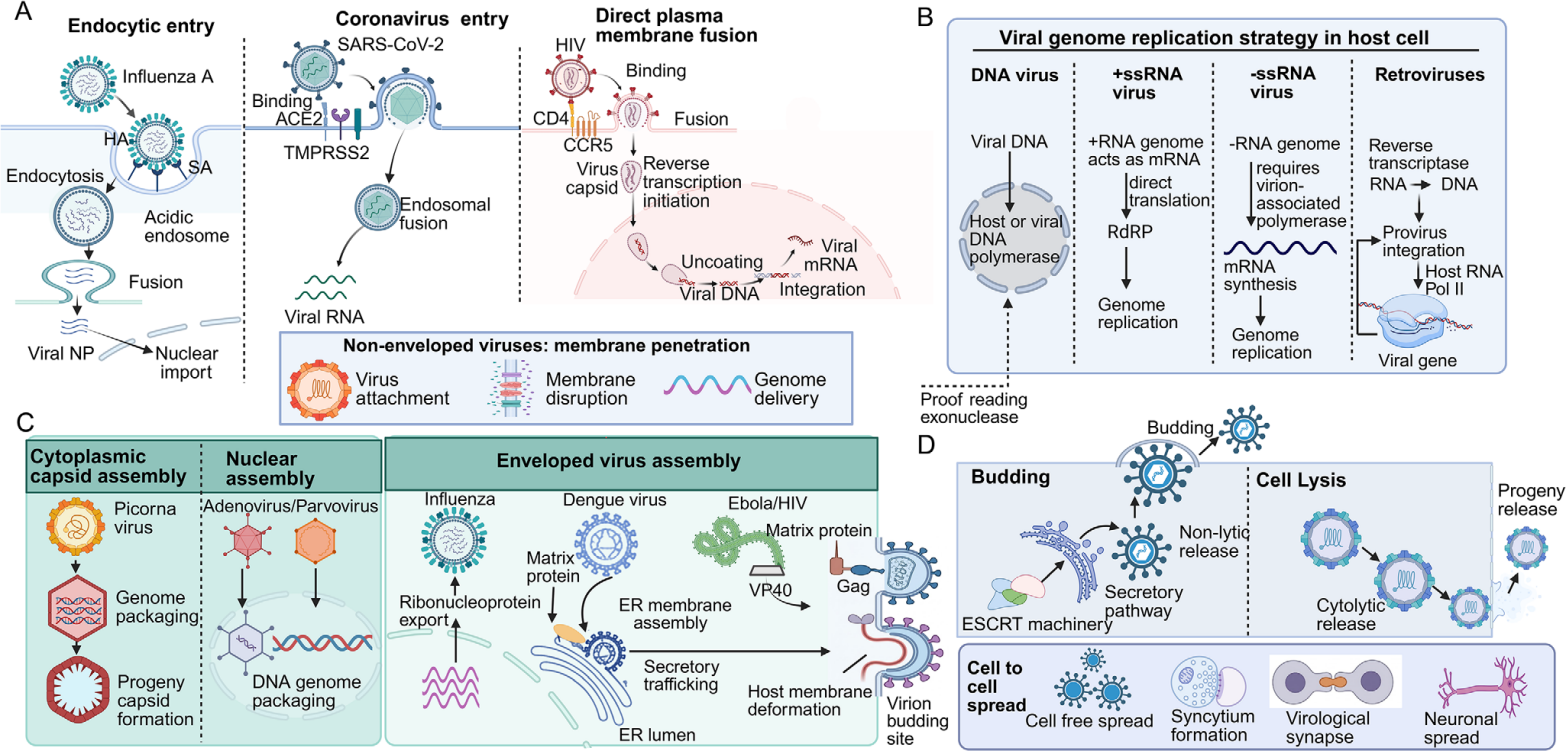
Overview of the viral life cycle. Viruses follow distinct but related steps during infection, including entry, genome replication, assembly, and egress. (A) Viral entry occurs through endocytosis, membrane fusion, or direct penetration, depending on the virus and host receptor usage. (B) Genome replication differs by genome type: DNA viruses use host or viral DNA polymerases, positive‐sense RNA viruses translate their genomes directly, negative‐sense RNA viruses require virion‐associated polymerases, and retroviruses reverse transcribe RNA into DNA for integration. (C) Virion assembly may occur in the cytoplasm, nucleus, or on intracellular membranes, followed by genome packaging and particle formation. (D) Newly formed virions exit by budding, secretion, or cell lysis and spread via cell‐free release, syncytia, virological synapses, or neuronal pathways.

These processes provide major targets for preventive and therapeutic intervention. nAbs can block receptor binding or fusion; receptor antagonists can prevent viral attachment; and fusion or host‐protease inhibitors can interrupt membrane entry. However, alternative entry pathways, variability in surface glycoproteins, and cell‐to‐cell fusion may reduce the durability of entry‐targeted interventions.

### 2.2 Genome Replication and Assembly

After entering the cell, viruses express their genes and replicate their genomes through strategies determined largely by genome type. DNA viruses generally replicate in the nucleus using host or virus‐encoded DNA polymerases, many of which possess proofreading activity and consequently have relatively low mutation rates [12, 17]. Most RNA viruses replicate in the cytoplasm using virus‐encoded RNA‐dependent RNA polymerases, although influenza virus replicates in the nucleus [18, 19]. Positive‐sense RNA genomes can function directly as messenger RNA, whereas negative‐sense RNA viruses must package a polymerase within the incoming virion to initiate transcription [18]. Retroviruses follow a distinct pathway in which reverse transcriptase converts viral RNA into DNA that integrates into the host genome [20, 21]. These essential enzymes provide the mechanistic basis for polymerase, reverse‐transcriptase, integrase, and protease inhibitors.

Replication fidelity strongly influences viral evolution and treatment durability. Most RNA‐dependent RNA polymerases and reverse transcriptases lack efficient proofreading, generating diverse viral quasispecies from which drug‐resistant or immune‐escape variants may be selected [22]. Excessive mutagenesis can, however, compromise viral viability, providing the basis for lethal‐mutagenesis strategies [23]. Coronaviruses are unusual among RNA viruses because nonstructural protein 14 provides proofreading exonuclease activity; loss of this function markedly increases mutation frequency and attenuates viral replication [22, 24].

Following replication, viral genomes and structural proteins assemble into progeny particles. Nonenveloped viruses assemble in the cytoplasm or nucleus, whereas enveloped viruses acquire lipid membranes by budding through cellular membranes [25, 26]. Influenza ribonucleoprotein complexes are exported from the nucleus and assemble with HA and neuraminidase (NA) at the plasma membrane [27, 28, 29, 30], whereas flaviviruses assemble and bud at the endoplasmic reticulum [31, 32, 33]. Matrix or related structural proteins coordinate genome packaging, membrane association, and budding; examples include Ebola virus VP40 [34, 35, 36], paramyxovirus M proteins, and HIV‐1 Gag [37, 38, 39]. Consequently, disruption of capsid formation, matrix interactions, genome packaging, or particle maturation can generate noninfectious progeny and represents an additional antiviral strategy.

### 2.3 Viral Egress and Spread

After assembly, viruses must exit the host cell and spread to new cells. Enveloped viruses commonly exit through budding, whereas many nonenveloped viruses are released by cell lysis or alternative vesicular pathways [40, 41, 42]. HIV‐1 Gag, for example, recruits the host endosomal sorting complexes required for transport machinery to promote membrane scission, and disruption of this interaction inhibits virion release [43, 44]. Some nonenveloped viruses actively induce cell death to facilitate egress, as illustrated by norovirus NS3‐mediated mitochondrial injury [45]. Viruses may also spread directly between cells through syncytia, virological synapses, neuronal connections, or tunneling nanotubes [46, 47]. Syncytium formation by paramyxovirus or SARS‐CoV‐2 fusion proteins [48, 49] and HIV transmission through virological synapses [50, 51, 52] can reduce exposure to nAbs. Herpesvirus movement through neuronal connections [53, 54], local influenza dissemination [55], and norovirus spread from intestinal cells [56, 57] further illustrate how tissue architecture facilitates transmission. Viral late‐domain motifs can recruit host budding machinery [34], whereas influenza NA promotes release by removing cell‐surface sialic acids. These mechanisms explain the activity of NA inhibitors and indicate why interventions that neutralize only extracellular virions may be less effective against direct cell‐to‐cell transmission.

### 2.4 Host Immune Responses

The host immune system detects and responds to viral infections through a combination of innate and adaptive mechanisms. These defenses, while essential for controlling viruses, also contribute to disease symptoms in many cases. Cells have evolved pattern‐recognition receptors that recognize molecular signatures of viruses (often called pathogen‐associated molecular patterns). Upon virus entry, intracellular sensors quickly detect viral components (Figure 2). Toll‐like receptors (TLRs) in endosomes are key sentinels for incoming viral genomes: TLR3 recognizes double‐stranded RNA (a replication intermediate for many viruses, or the genome of reoviruses); TLR7 and TLR8 detect single‐stranded RNA (such as HIV or influenza genomic RNA) rich in certain motifs; and TLR9 detects unmethylated CpG motifs in viral DNA (as found in herpesvirus genomes) [58]. These TLRs are strategically localized in endosomes so they can sample viral genomes that are released during entry (but not be triggered by the host's own nucleic acids, which are usually segregated in the nucleus or cytoplasm). In the cytosol, RNA viruses are chiefly sensed by RIG‐I‐like receptors (RLRs). The RLR family includes RIG‐I and MDA5, which are RNA helicases that bind viral RNA and signal for interferon (IFN) production. RIG‐I is activated by RNA bearing a 5′‐triphosphate or diphosphate end (a feature of many viral RNAs that lack the 5′ cap of cellular mRNAs) and typically detects short double‐stranded or panhandle RNA structures, whereas MDA5 detects long dsRNA [59]. Upon binding viral RNA, RIG‐I and MDA5 interact with the adaptor protein MAVS on mitochondria, triggering a signaling cascade that activates IFN regulatory factors (IRF3/7) and NF‐κB to induce antiviral genes [60, 61]. DNA viruses that enter the cytoplasm (or DNA that leaks from the nucleus during infection) are sensed by cGAS (cyclic GMP–AMP synthase). cGAS binds to cytosolic viral DNA (for example, DNA from herpesviruses or adenoviruses) and produces the second‐messenger cGAMP, which binds and activates STING on the endoplasmic reticulum membrane [62]. STING then triggers pathways that lead to IRF3 and NF‐κB activation, much like the RLR pathway, culminating in IFN production. This cGAS–STING pathway is critical for detecting DNA viruses; for instance, cells lacking cGAS or STING fail to produce IFN in response to cytoplasmic DNA and are much more susceptible to DNA virus infection [62, 63]. Other sensors also contribute (NOD‐like receptors can recognize viral RNA or DNA and trigger inflammasomes, while certain helicases and cytosolic DNA‐binding proteins feed into antiviral responses), but TLRs, RLRs, and cGAS–STING are the main initiators of the antiviral IFN response. These pathways are potential targets for IFNs, innate immune agonists, and other host‐directed interventions.

**FIGURE 2:**
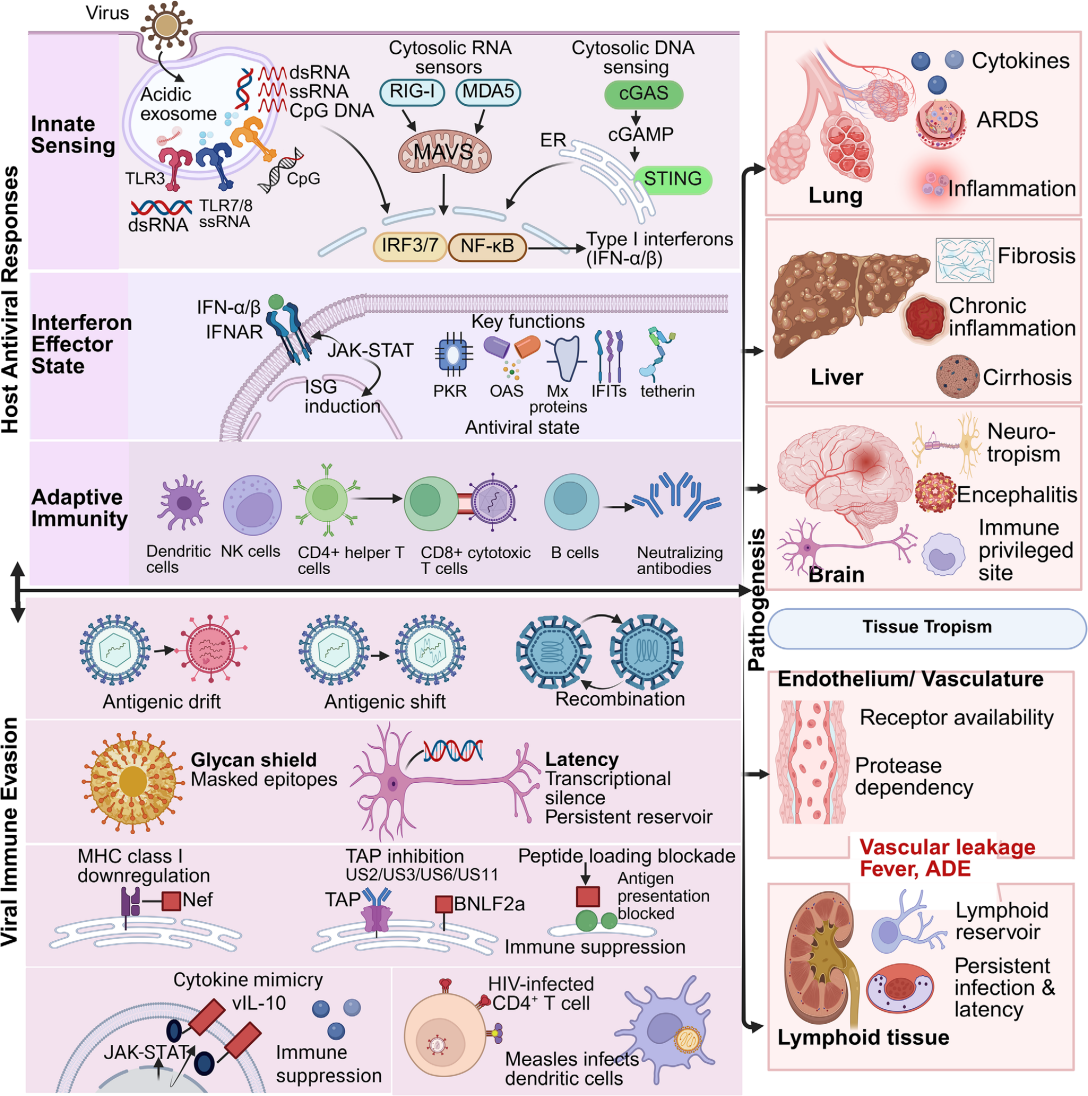
Host antiviral responses, viral immune evasion, and tissue tropism. Viral infection activates innate sensing through endosomal and cytosolic receptors, including TLRs, RIG‐I/MDA5, and cGAS–STING, leading to IRF3/7 and NF‐κB signaling and induction of Type I interferons. Interferon‐driven JAK–STAT signaling establishes an antiviral state by inducing restriction factors such as PKR, OAS, Mx proteins, IFITs, and tetherin. Adaptive immunity is mediated by dendritic cells, NK cells, CD4+ helper T cells, CD8+ cytotoxic T cells, and B cells. Viruses counteract host defense through antigenic drift and shift, glycan shielding, epitope masking, latency, immune suppression, and interference with antigen presentation. On the right side of figure, it shows tropism and disease outcomes in the lung, liver, brain, endothelium, and lymphoid tissues, including inflammation, fibrosis, vascular leakage, and persistent infection.

Type I IFNs signal through the Janus kinase (JAK)–STAT pathway and induce IFN‐stimulated genes that inhibit viral transcription, translation, genome replication, assembly, and release [64]. They also enhance natural killer‐cell activity, dendritic‐cell function, and major histocompatibility complex Class I antigen presentation [65]. Although this broad response creates a high barrier to viral resistance, systemic IFN therapy can be limited by toxicity, and excessive innate activation may contribute to immunopathology.

Adaptive immunity provides antigen‐specific and durable protection. nAbs block attachment or membrane fusion by targeting viral surface proteins, including influenza HA, HIV‐1 Env, and SARS‐CoV‐2 spike [66]. They can also promote viral clearance through aggregation, opsonization, complement activation, and Fc receptor‐mediated mechanisms. Neutralizing‐antibody titers are therefore important correlates of protection for many vaccines, although rapidly evolving viruses may escape pre‐existing antibodies [66].

CD8^+^ cytotoxic T lymphocytes recognize viral peptides presented by major histocompatibility complex Class I molecules and eliminate infected cells through cytolytic and cytokine‐mediated mechanisms [67]. Defective CD8^+^ T‐cell responses can impair control even when antibodies are present [68, 69]. CD4^+^ T cells support B‐cell maturation, cytotoxic T‐cell activity, macrophage activation, and immunological memory [70]. Natural killer cells and dendritic cells further connect innate and adaptive immunity by eliminating cells with reduced major histocompatibility complex expression and initiating T‐cell responses. Vaccines that induce coordinated antibody, CD4^+^ T‐cell, and CD8^+^ T‐cell immunity may therefore provide broader protection than those relying on a single immune mechanism.

### 2.5 Viral Immune Evasion Strategies

Given the effectiveness of host immune responses, viruses have evolved numerous evasion strategies to persist in the host. These strategies allow viruses to avoid detection, prevent destruction by immune cells, or modulate the immune response to their advantage. One of the most direct ways viruses evade immunity is by changing the very targets that immune molecules recognize. High mutation rates in RNA viruses facilitate antigenic drift, the gradual accumulation of mutations in viral proteins that are recognized by antibodies or T cells. A classic example is the influenza virus: point mutations in the HA and NA surface proteins accumulate year to year, altering key epitopes so that existing antibodies from prior infections or vaccines bind less effectively [71]. This antigenic drift is why influenza vaccines must be updated frequently and why individuals can be reinfected with new seasonal flu strains [71]. Antigenic variation is especially pronounced in HIV‐1 as well—the error‐prone reverse transcriptase leads to a “swarm” of HIV variants in each patient, and immune pressure selects for escape mutants. nAbs that initially suppress HIV are circumvented by single amino acid changes in Env glycoprotein, and Cytotoxic T lymphocytes (CTLs) targeting HIV peptides are escaped by mutations that disrupt the peptide–major histocompatibility complex (MHC) recognition. Some viruses also employ antigenic shift or recombination: influenza can reassort its segmented genome with that of other influenza strains when two strains coinfect a cell, suddenly creating a novel HA or NA that the population's immune systems have never seen (e.g., the emergence of pandemic H1N1 or H3N2 strains). Other viruses like dengue have multiple serotypes; infection with one serotype leads to immunity to that serotype but not the others, and the viral population as a whole persists via serotype diversity.

Many viruses cloak their epitopes under a “shield” of glycans (sugars) to hide from antibody recognition. Viruses that heavily glycosylate their envelope proteins include HIV‐1, Lassa fever virus, and coronaviruses, among others. HIV‐1 Env contains approximately 25–30 N‐linked glycans per protomer, forming a dense trimeric glycan shield that can sterically hinder antibody binding [72]. These glycans are self‐carbohydrates derived from the host and thus are seen as “self” by the immune system (nonimmunogenic). Only a few holes in this glycan shield expose protein surfaces, and effective broadly nAbs (bnAbs) against HIV often target such sites (or even recognize the glycans themselves in a specific orientation). Similarly, the SARS‐CoV‐2 spike has numerous N‐linked glycans that shield its receptor‐binding domain and fusion machinery, although not as densely as HIV's Env. Ebola virus GP is also heavily glycosylated with both N‐ and O‐linked sugars that mask its conserved binding sites. Glycan shielding does not entirely prevent immune recognition but forces the immune system to target more variable, protruding loops or scarce unglycosylated sites [73, 74]. This contributes to immune evasion and presents a challenge for vaccine design, as immunogens must induce antibodies that either penetrate the glycan shield or target less accessible conserved elements [72]. Some viruses also vary the positions of glycosylation (glycan shift) as an evolutionary strategy to escape antibodies (e.g., HIV can add/remove glycosylation sites on Env).

Perhaps the most effective immune evasion is to simply hide from the immune system by not producing any antigen—this is the strategy of viral latency. Latency is a state of infection in which the virus largely shuts down gene expression and replication, surviving as a dormant genome within the host cell [75]. Many herpesviruses are masters of latency. Herpes simplex virus (HSV) invades peripheral neurons and establishes latent infection in the trigeminal or dorsal root ganglia, with limited viral gene expression, mainly latency‐associated transcripts and viral noncoding RNAs [76, 77]. In this state, latently infected neurons are essentially invisible to CD8 T cells, because there are no viral peptides to display on MHC Class I [78]. The virus can persist for the lifetime of the host in this protected state, periodically reactivating (when triggered by stress or immunosuppression) to cause recurrent lytic infection (e.g., cold sores or zoster rash) before latency is re‐established. Retroviruses like HIV establish a form of latency by integration of the viral DNA into the host genome. If an HIV‐infected CD4^+^ T cell becomes quiescent (memory T cell), the integrated provirus may lie transcriptionally silent—no viral proteins are made, so the cell is not recognized or killed by the immune system. These latently infected cells form a reservoir that persists despite antiviral therapy and immune responses [79]. The immune system cannot easily eliminate what it cannot see. The latent reservoir of HIV has a long half‐life (estimated ∼44 months) and is the barrier to curing the infection [79]. Other viruses that integrate, like human endogenous retroviruses in germline DNA, also evade immunity by essentially becoming “self.” Even without physical genome integration, some viruses establish chronic persistence in immune‐privileged sites. For example, JC polyomavirus persists in the kidney tubules and can hide from immunity unless reactivated causing PML in the brain under immunosuppression [80]. Overall, latency and integration allow viruses to wait out adverse conditions (like a robust immune response) and reactivate when the coast is clear. During latency, viruses like Epstein–Barr virus (EBV) and HIV may express a few selected genes that help maintain the latent state or modulate the host—but often these are designed not to alarm the immune system (EBV expresses latency proteins in B cells that skew the immune response or remain at low levels).

To avoid cytotoxic T lymphocytes, many viruses actively interfere with the host's antigen presentation pathway. One common target is the MHC Class I pathway. For CD8 T cells to recognize an infected cell, viral peptides must be presented on MHC I on the cell surface. Viruses such as adenovirus, cytomegalovirus (CMV), EBV, and HIV have all evolved proteins to interrupt this pathway. CMV is notorious for this: it produces multiple proteins (US2, US3, US6, US11 in human CMV) that block different steps of MHC I processing and trafficking—some cause degradation of MHC I heavy chains in the ER, another (US6) prevents peptide loading by inhibiting the TAP transporter [81, 82]. The end result is infected cells with little MHC I on their surface, so CD8 T cells cannot detect viral peptides (at least not from those viruses) and infected cells are spared T‐cell killing. HIV‐1 encodes the accessory protein Nef, which has a well‐documented function of downregulating MHC I from the surface of infected cells. Nef binds to the cytosolic tails of MHC I and reroutes them to lysosomes for degradation or prevents their transport to the plasma membrane [67]. This Nef‐mediated MHC I downregulation makes HIV‐infected T cells much less visible to CTLs, allowing HIV to replicate within T cells even in the presence of active virus‐specific CTLs. However, many viruses that do this face a trade‐off: by reducing MHC I, they can become targets for NK cells (since NK cells kill cells with absent MHC I—the “missing‐self” recognition). Some viruses therefore selectively downregulate certain MHC I alleles (e.g., HIV Nef downregulates HLA‐A and ‐B [restricting CTLs] but not HLA‐C or ‐E, which engage NK cell inhibitory receptors)—a nuanced evasion to avoid T cells while placating NK cells [83]. EBV during its lytic cycle expresses BNLF2a, a protein that inhibits the TAP peptide transporter, thereby preventing antigen presentation on MHC I; simultaneously, EBV produces vIL‐10 to reduce immune surveillance. These mechanisms underscore how important antigen presentation is—viruses devote genes to shutting it off.

Viruses also evade immunity by actively modulating the host immune response. A striking example is EBV, which encodes a viral homolog of interleukin‐10, called vIL‐10 (encoded by the BCRF1 gene). IL‐10 is an anti‐inflammatory cytokine that suppresses Th1 responses and cell‐mediated immunity. EBV's vIL‐10 can bind IL‐10 receptors and mimic the effects of host IL‐10, dampening the immune response during the early phase of infection [84]. This results in reduced production of proinflammatory cytokines and decreased activation of T cells and macrophages, allowing EBV‐infected B cells to evade elimination. EBV also expresses BNLF2a, which, as noted, blocks antigen presentation by TAP and protects newly infected B cells from CTLs [84]. Together, vIL‐10 and BNLF2a help EBV establish a foothold in the host by creating an immunosuppressive microenvironment and hiding viral antigens [85]. Another well‐known strategy is direct infection of immune cells. HIV infects CD4^+^ T helper cells, progressively depleting them and causing immunodeficiency, which allows the virus and other pathogens to proliferate unchecked. Measles virus transiently suppresses the immune system by infecting dendritic cells and memory T and B cells, leading to what's called “immune amnesia”—after measles, children become susceptible to other infections they once had immunity too [86, 87]. Many viruses encode cytokine decoys or inhibitors. Poxviruses are famous for producing secreted versions of cytokine receptors like a soluble TNF receptor or IL‐1 receptor that bind and neutralize cytokines, preventing those cytokines from doing their job in antiviral defense [88, 89, 90]. Herpesviruses and CMV encode chemokine homologs that attract cells favorable for infection or away from the infection site [91]. Viruses can also interfere with immune signaling pathways; for instance, adenoviruses produce RID proteins that inhibit death receptors and thus avoid apoptosis, and many viruses express proteins to block the NF‐κB pathway, IFN production, or IFN signaling [92, 93]. By broadly suppressing or misdirecting the immune system, viruses can buy time to replicate and spread. A double‐edged consequence of such immunosuppression is that it can render the host susceptible to secondary infections (as in HIV or measles cases). From the virus's perspective, however, disabling certain arms of immunity (like T cells, NK cells, or the complement system) greatly enhances its survival. Successful human pathogens often strike a balance: they disable enough of the immune response to persist, but not so much that the host dies immediately (at least in most cases), as the virus's survival often depends on the host surviving long enough to transmit.

### 2.6 Antiviral Resistance: Emergence, Surveillance, and Mitigation

Antiviral resistance arises when ongoing viral replication under drug pressure selects variants that reduce drug binding, alter the targeted viral process, or compensate for the fitness costs imposed by primary resistance mutations. The probability of resistance is influenced by viral population size and mutation rate, target conservation, the number of mutations required to confer clinically meaningful resistance, drug exposure, treatment adherence, and the presence of other fully active agents [94, 95, 96]. Consequently, antiviral potency alone does not determine durability; the genetic barrier to resistance and the pharmacological context in which a drug is administered are equally important.

Nirmatrelvir inhibits the SARS‐CoV‐2 main protease, Mpro or 3CLpro, which is required for processing viral polyproteins. Experimental selection studies have identified several mutational pathways that reduce nirmatrelvir susceptibility, including substitutions at E166, S144, L167, and other residues within or surrounding the inhibitor‐binding pocket [97, 98]. Among these, E166V produced an approximately 100‐fold reduction in susceptibility but impaired viral replication, whereas accompanying substitutions such as L50F or T21I restored replicative fitness. E166V has also been identified in viral isolates from some nirmatrelvir‐treated participants [99]. Nevertheless, these resistance‐associated substitutions have generally remained uncommon in population‐level sequence datasets, emphasizing the need to distinguish experimentally or clinically observed resistance pathways from widespread transmission of resistant variants.

Resistance is also clinically important for lenacapavir, a long‐acting HIV‐1 capsid inhibitor. Capsid substitutions such as M66I, Q67H/K/N, K70N/R/S, N74D, A105T, and T107A/C/S have emerged during virological failure [100, 101, 102, 103, 104]. M66I is particularly important because it can produce a marked reduction in lenacapavir susceptibility [105]. In the CAPELLA study, resistance‐associated capsid substitutions were detected in 16 out of 28 participants with confirmed virological failure who had postbaseline genotypic data [106]. Resistance occurred particularly when lenacapavir effectively functioned as monotherapy because the accompanying regimen contained no fully active agents or adherence to companion antiretrovirals was inadequate.

Resistance mitigation requires coordinated virological, pharmacological, and clinical measures. Baseline treatment history and resistance profiles should guide regimen selection, while viral‐load monitoring and genotypic or phenotypic resistance testing should be undertaken when virological failure or rebound occurs. For emerging infections such as SARS‐CoV‐2, sequencing of viruses obtained after persistent infection or treatment failure, together with population‐level genomic surveillance, can identify resistance‐associated substitutions before they become widely disseminated [107, 108]. Whenever supported by clinical evidence, combining agents with independent targets can increase the barrier to resistance and reduce the probability that a single viral genome will contain all mutations required for escape. For HIV, lenacapavir should be administered with an optimized regimen containing fully active companion agents, and adherence to both the long‐acting injection schedule and oral background therapy is essential. Because residual lenacapavir concentrations can persist for 12 months or longer, missed injections or discontinuation should be managed with appropriate oral bridging or timely initiation of an alternative fully suppressive regimen [109]. Comparisons of resistance barriers should therefore consider the number and type of required mutations, changes in phenotypic susceptibility, effects on viral fitness, pharmacokinetic (PK) exposure, the duration of residual drug concentrations, and the activity of accompanying agents rather than relying solely on in vitro potency.

### 2.7 Pathogenesis and Tissue Tropism

The pathology caused by viruses can result from direct damage to infected cells. Viruses that cause acute lytic infections often produce dramatic cytopathic effects (CPEs) in cells: they subvert the host cellular machinery to replicate, which can lead to shutdown of host protein synthesis, degradation of cellular DNA, alterations in cell membrane permeability, and ultimately cell death. For instance, poliovirus causes lysis of motor neurons in the spinal cord by hijacking the host ribosomes and inducing cell death—the loss of these neurons manifests as paralysis [110]. Many viruses induce apoptosis or necrosis in infected cells as a mechanism of egress, facilitating viral dissemination. When such CPEs occur extensively within essential tissues, they directly drive disease pathogenesis. For instance, rotavirus‐mediated destruction of intestinal epithelial cells compromises absorptive function and results in diarrheal disease [111, 112], whereas yellow fever virus‐induced hepatocyte death leads to significant hepatic injury [113]. Some viruses induce unique cytopathic features: rabies virus infection leads to Negri bodies in neurons [114]; herpesviruses can cause large nuclear inclusions [115, 116]; poxviruses cause cytoplasmic factories [117, 118]. These inclusions are often virus assembly sites but can disrupt normal cell architecture. Syncytium formation is another direct effect—in RSV infection of the lungs, multinucleated giant cells form and their eventual death exposes the lung tissue to inflammation and fluid accumulation [119]. Cytopathic viruses can also promote cancer indirectly through chronic tissue damage and repeated regeneration. By contrast, viruses such as HPV can more directly drive proliferation through viral oncogenes, but this is a long‐term effect rather than an acute cytopathic one [120, 121, 122]. The severity of direct cytopathic injury often correlates with disease severity. Eastern equine encephalitis virus can cause encephalitis with high fatality because neurons have limited regenerative capacity [123]. On the other hand, some viruses cause minimal CPEs in certain cells, leading to chronic infection without obvious immediate damage. Many persistent hepatitis B infections have low‐level viral replication with little liver damage until immunopathologic mechanisms kick in [124, 125].

A significant component of viral pathogenesis is due to the immune response itself. While immune responses are essential for controlling infection, they can unintentionally damage host tissues—a phenomenon often referred to as immunopathology. One extreme example is the “cytokine storm” observed in severe cases of influenza (such as the 1918 pandemic flu) and COVID‐19. In these situations, an initial antiviral immune response becomes dysregulated, leading to excessive production of proinflammatory cytokines (IL‐6, IL‐1β, TNF‐α, IFN‐γ, etc.) and the recruitment of large numbers of leukocytes to the infection site. This hyperinflammatory state can cause more damage than the virus itself. In COVID‐19, for instance, an aggressive immune response can lead to acute respiratory distress syndrome (ARDS)—the lungs fill with inflammatory fluid, and extensive tissue damage occurs, which has been a major cause of mortality in severe SARS‐CoV‐2 infection [126]. Similarly, in H5N1 avian influenza and SARS, researchers noted that an overwhelming IFN and cytokine response was associated with fatal outcomes [127]. Another example of immunopathology is dengue hemorrhagic fever: a phenomenon called antibody‐dependent enhancement (ADE) can occur when a person with immunity to one dengue serotype gets infected by a different serotype [128, 129]. Non‐nAbs from the first infection form complexes with the new virus and facilitate its entry into Fc‐receptor‐bearing cells (like monocytes), leading to increased viral load and a robust activation of memory T cells and cytokine release. The result is a systemic inflammatory response with plasma leakage, hemorrhaging, and potentially shock—much of this damage is due to the immune response rather than direct viral cytopathy. Chronic viral infections also illustrate immunopathology: in chronic hepatitis B or C, continuous activation of virus‐specific T (VST) cells and other immune cells in the liver leads to fibrosis and cirrhosis over years—the liver damage is largely a result of the immune system killing infected cells and secreting inflammatory mediators, gradually destroying liver architecture [125, 130, 131, 132, 133]. In certain viral infections of the brain (viral encephalitis), inflammatory swelling and demyelination in diseases like SSPE or PML can cause severe neurological deficits; sometimes reducing inflammation (with corticosteroids) is a part of therapy to prevent permanent damage. Thus, viral diseases often have a two‐edged nature: the virus injures cells, and the immune system's attempt to clear the virus can collateral damage tissues. A balanced immune response typically results in virus clearance with minimal pathology, whereas an unbalanced one (too weak or too strong) can lead to either uncontrolled viral spread or immunopathology. This understanding has led to therapeutic strategies that include immunomodulation—for example, using steroids, IL‐6 blockers, or JAK inhibitors in severe COVID‐19 to tone down the cytokine storm [126, 134, 135].

Different viruses are specialized to infect particular cell types and organs—their tissue tropism—which largely dictates the clinical syndrome they cause. Tropism is determined by factors like receptor availability, cell proteases needed for entry, temperature sensitivities, and intracellular host factors that permit replication. For instance, the neurotropism of rabies virus stems from its ability to bind neuronal cell adhesion molecules (like the acetylcholine receptor) at neuromuscular junctions and travel within axons to the central nervous system [136]. Consequently, rabies infection leads to encephalitis with symptoms like seizures, paralysis, and hydrophobia. Poliovirus is also neurotropic (binding to the PVR receptor on motor neurons), causing poliomyelitis (flaccid paralysis) when those neurons are destroyed [137, 138]. Viruses like hepatitis B and C are hepatotropic—they infect hepatocytes, likely because those cells have the entry receptors (NTCP, sodium taurocholate co‐transporting polypeptide for HBV; and various factors for HCV) and supportive environment for the virus. The result is liver‐specific disease (hepatitis, jaundice, potential liver failure, or cancer). Some viruses have a very narrow cell tropism: for example, human papillomaviruses (HPVs) infect keratinocytes of the skin or mucosa and no other cell type, which is why HPV causes warts or cervical cancer in those tissues and nowhere else [139]. In contrast, certain viruses are pantropic (infect many cell types) but may still cause predominant symptoms in particular organs based on entry route or immune targeting. Tropism also influences how severe an infection is—infection of immune‐privileged sites like the central nervous system (CNS) or eyes can be particularly dangerous. For example, viral hemorrhagic fevers like Ebola involve viruses that not only cause cytopathic damage but also target endothelial cells and cells of the immune system, leading to widespread vascular leakage [140, 141].

Some tissues act as reservoirs where viruses persist long‐term, and these often coincide with limited immune surveillance. The central nervous system (brain and spinal cord) is one such site—immune responses in the CNS are relatively restrained (to avoid immunopathology in such a critical area), and the blood–brain barrier limits entry of immune cells and molecules. HIV provides a clear example: HIV can establish infection in brain microglia and macrophages; the brain then becomes a sanctuary site where the virus is somewhat shielded from both antiretroviral drugs (many of which penetrate the CNS poorly) and immune clearance [142]. Studies have found that HIV can be detected in the brain even in patients on suppressive ART, and the virus can evolve separately in the CNS [142]. This may contribute to viral persistence and compartmentalized rebound risk. The lymphoid tissue is another reservoir: for HIV, lymph node follicles harbor infected T follicular helper cells, and these sites are somewhat inaccessible to CD8 T cells under normal conditions. Viruses like EBV and CMV reside in immune cells (B cells for EBV, myeloid cells for CMV) within lymphoid organs, using latent programs to avoid cytotoxic cells [143, 144]. The bone marrow can hide viruses (parvovirus B19 persists in erythroid precursors) [145, 146], and the eye and testes are immune‐privileged (Ebola virus has been known to persist in the eyes and testes of survivors for months, causing uveitis or being sexually transmitted long after recovery) [147]. From a treatment perspective, these reservoir sites pose a huge challenge—for instance, drug delivery to the CNS requires drugs that cross the blood–brain barrier (many antivirals do not, or do so in low concentrations). Similarly, clearing latent proviruses requires flushing them out of hiding (which is the goal of “shock and kill” strategies in HIV cure research—to induce latent viruses so infected cells become visible to the immune system). Tissue tropism also affects drug development: if a virus is extracellular (like in the blood), antibodies or soluble receptor decoys might work; if it is intracellular in specific organs, you need antivirals that reach those cells in effective concentrations.

## 3 Prophylactic Strategies and Agents

Prophylactic strategies against viral diseases encompass active immunization, passive antibody‐based protection, chemoprophylaxis, and population‐level interventions designed to prevent infection, reduce disease severity, or interrupt transmission. Vaccines remain the cornerstone of prevention by inducing durable humoral and cellular immunity through diverse platforms, including live‐attenuated, inactivated, protein‐based, viral‐vector, and nucleic‐acid technologies [148, 149, 150]. mAbs provide immediate protection for individuals who are at high risk, immunocompromised, or unlikely to mount adequate vaccine responses, whereas antiviral agents can be deployed before or shortly after exposure as pre‐exposure or postexposure prophylaxis [151, 152]. The effectiveness of these approaches depends not only on biological potency but also on PK, dosing frequency, adherence, antigenic evolution, and accessibility. In this section, we examine the major vaccine platforms and their immunological correlates of protection, the prophylactic and therapeutic applications of mAbs, the principles of pre‐ and postexposure prophylaxis, and the programmatic considerations that influence implementation. It further considers population‐level measures, including mass and ring vaccination, passive immunization programs, and vector‐control strategies, which collectively extend prophylaxis from individual protection to community‐wide suppression of viral transmission.

### 3.1 Vaccines

Vaccines are biological preparations that provide acquired immunity against specific infectious diseases by safely mimicking natural infection and training the immune system to recognize and eliminate pathogens. By introducing a harmless antigen or genetic blueprint of a pathogen, they activate immune cells to produce antibodies and T‐cell responses while also generating memory cells for long‐term protection [153, 154]. As a result, the body can mount a rapid and effective defense upon future exposure to the real pathogen, without experiencing the disease itself. Key vaccine components include the antigen, which is the active ingredient that stimulates the immune system; adjuvants, which are added to enhance the immune response to the antigen; and stabilizers or preservatives, which help maintain vaccine effectiveness and prevent contamination [149]. Vaccines are among the most effective tools in modern medicine for preventing viral disease by safely priming the immune system to recognize and respond to pathogen‐specific antigens (Figure 3). By presenting weakened, inactivated, or molecular components of a virus, vaccines stimulate the production of antibodies, activate T‐cell responses, and generate long‐lived memory cells that enable a rapid and coordinated defense upon later exposure to the authentic pathogen [155, 156]. Viral vaccines are developed using several platform technologies (including live‐attenuated, inactivated, protein subunit, virus‐like particle, viral vector, and nucleic acid approaches), each designed to present viral antigens to the immune system in a different way [149, 157, 158]. Unlike conventional drugs that act through a single defined target, vaccines engage multiple layers of innate and adaptive immunity, making their mechanisms of protection highly complex but exceptionally effective [153].

**FIGURE 3:**
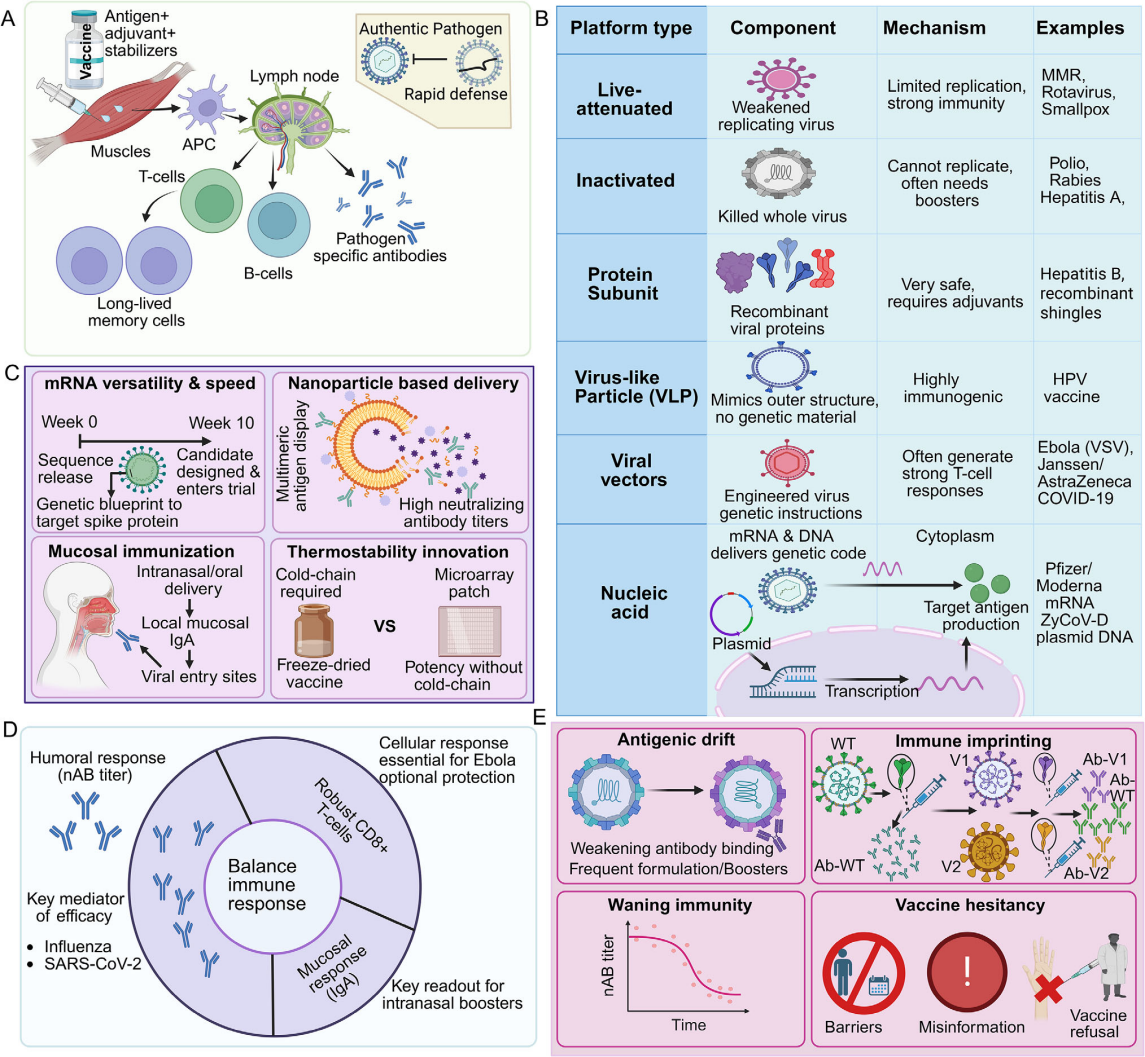
Major vaccine platforms, delivery innovations, and immunological considerations for antiviral vaccination. (A) Vaccination delivers antigen with adjuvants to activate antigen‐presenting cells, drain to lymph nodes, and induce T‐ and B‐cell responses, long‐lived memory, and neutralizing antibodies. (B) Major vaccine platforms include live‐attenuated, inactivated, protein subunit, virus‐like particle, viral vector, and nucleic acid‐based vaccines, each differing in safety, immunogenicity, and mechanism of antigen presentation. (C) Emerging strategies improve speed, versatility, mucosal immunity, and thermostability, including mRNA vaccination, nanoparticle‐based delivery, intranasal/oral administration, and cold‐chain‐independent formulations. (D) Protective efficacy depends on a balanced humoral, cellular, and mucosal immune response, with different readouts relevant for distinct pathogens. (E) Vaccine performance is influenced by antigenic drift, immune imprinting, waning immunity, and vaccine hesitancy, which together shape durable protection and booster requirements.

Live‐attenuated vaccines contain a weakened form of the virus that can still replicate to a limited extent, usually inducing strong and long‐lasting immunity; examples include measles–mumps–rubella, rotavirus, and vaccinia‐based smallpox vaccination [158]. Inactivated vaccines are made from viruses that have been killed by chemical, heat, or radiation treatment, so they cannot replicate and often require booster doses; examples include polio, hepatitis A, and rabies vaccines [158]. Protein subunit vaccines contain only selected viral proteins, often produced by recombinant methods, and are generally very safe but may need adjuvants to strengthen the immune response; examples include hepatitis B and recombinant shingles vaccines [159]. Virus‐like particle vaccines mimic the outer structure of a virus without containing any genetic material, making them highly immunogenic; the HPV vaccine is a classic example [158]. Viral vector vaccines use a harmless engineered virus to deliver genetic instructions for a viral antigen into host cells and often generate strong T‐cell responses; examples include the Ebola vaccine based on vesicular stomatitis virus and the adenoviral vector COVID‐19 vaccines from Janssen and AstraZeneca [160]. Nucleic acid vaccines, including mRNA and DNA vaccines, deliver the genetic code for the antigen so that host cells produce the viral protein themselves; mRNA vaccines such as Pfizer‐BioNTech and Moderna deliver this message in lipid nanoparticles to the cytoplasm, whereas DNA vaccines (e.g., ZyCoV‐D, a plasmid DNA COVID‐19 vaccine developed by Zydus) must first enter the nucleus before being transcribed into mRNA [161].

Vaccines are mechanistically more complex than most pharmaceutical agents because they do not act through a single ligand–receptor interaction. Instead, vaccine antigens and adjuvants engage multiple components of the immune system locally at the site of immunization and, in some cases, at systemic sites, thereby priming an anamnestic response to a later infectious challenge that may occur at a different anatomical site and long after vaccination. This response can be further complicated by antigenic differences between the vaccine and the infecting virus, as well as by prior exposure to related viruses, making vaccine immunology a particularly rich and fascinating area of study. In the past decade, nucleic‐acid vaccines (mRNA and DNA) and self‐amplifying RNA replicons have emerged as game‐changers, alongside advances in nanoparticle‐based antigen delivery [162, 163]. Mucosal immunization strategies (intranasal and oral) are also being explored to induce local immunity at viral entry sites [164, 165, 166, 167]. Each platform has distinct advantages: live‐attenuated vaccines elicit robust broad immunity but carry slight safety risks; inactivated and protein subunit vaccines are safer but often less immunogenic; viral vectors can induce strong T‐cell responses; and mRNA vaccines offer rapid, “plug‐and‐play” design for new pathogens [162, 163]. This versatility was exemplified in the COVID‐19 pandemic, where within weeks of SARS‐CoV‐2's sequence release, an mRNA vaccine candidate was designed and entered human trials just 66 days later [168]. The successful deployment of mRNA vaccines in 2020–21 has firmly established this platform, enabling unprecedented speed in development and facile updating of vaccine strains.

The mRNA vaccine platform demonstrated the power of rapid redesign, as seen with variant‐adapted COVID‐19 booster shots targeting emerging strains within a year of their appearance [169]. Protein subunit vaccines formulated as nanoparticles have also shown promise; for example, a recombinant nanoparticle vaccine for SARS‐CoV‐2 induced high nAb titers and gained authorization, validating that multimeric antigen display can enhance immunogenicity [170, 171]. Novel thermostable formulations are being pursued to ease distribution constraints—approaches like freeze‐dried vaccines and microarray patches are being developed and have shown promise for improving thermostability and delivery [172, 173, 174]. Such heat‐stable and needle‐free vaccine technologies could greatly expand immunization in resource‐limited settings.

A critical aspect of vaccine design and evaluation is identifying immunological correlates of protection. For many viral vaccines, the strength of the humoral response—often measured by nAb titers—is a key predictor of efficacy [175, 176, 177, 178]. For instance, an influenza hemagglutination‐inhibition antibody titer of 1:40 has historically been associated with ∼50% protection in young adults [175]. High nAb levels against SARS‐CoV‐2 similarly correlate with reduced infection risk and disease severity in COVID‐19 [179, 180, 181]. However, antibody correlates do not tell the whole story. Robust T‐cell immunity can be essential for viruses where antibodies alone are insufficient. T‐cell responses may contribute to protection for some viral vaccines [175, 182, 183]. Mucosal IgA is another important correlate for respiratory and enteric viruses—for example, secretory IgA in nasal fluids correlates with protection against influenza and has become a key readout for intranasal COVID‐19 boosters [175, 184]. These observations have guided vaccine development to aim for a balanced immune response. Next‐generation vaccines are evaluated on multiple immune parameters (neutralizing IgG, mucosal IgA, memory T‐cell responses, etc.) as surrogate endpoints of protection [175, 184, 185, 186]. Regulatory reliance on such correlates was evident in COVID‐19, where immune markers allowed vaccine updates without lengthy efficacy trials [178, 181, 187].

Despite major advances, several challenges continue to limit the full effectiveness of viral vaccines. Antigenic drift, particularly in RNA viruses such as influenza and SARS‐CoV‐2, allows mutations to accumulate and weaken vaccine‐induced protection, often necessitating frequent reformulation of influenza vaccines and updated boosters for emerging variants such as Omicron [181, 188, 189, 190, 191, 192, 193, 194, 195]. In addition, immune imprinting can bias responses toward earlier antigens and reduce responsiveness to newer variants, making strategies such as antigenically distinct boosters or mosaic antigen designs an active area of investigation [192, 196, 197, 198, 199]. Vaccine hesitancy further undermines uptake, with misinformation, lack of trust, and access barriers contributing to poor coverage and resurgences of preventable diseases; this issue was recognized by the World Health Organization as a major global health threat [200, 201, 202]. Finally, waning immunity remains a practical concern for many vaccines, as some induce long‐lasting protection whereas others require periodic boosting, particularly in the face of declining nAbs and evolving viral variants (including coronaviruses), highlighting the need to balance booster timing, antigen selection, and public acceptance [200, 201, 202, 203].

### 3.2 Monoclonal Antibodies

mAbs have become an important form of passive immunization for viral diseases, complementing vaccines by providing immediate, preformed protection, especially in high‐risk or vaccine‐limited populations. Their prophylactic value is well illustrated by palivizumab, an anti‐RSV mAb that has been used since the late 1990s to protect premature infants and those with chronic lung or heart disease during RSV season [204, 205], with clinical studies showing substantial reductions in RSV hospitalization rates (∼39–78%) [206, 207]. However, palivizumab requires monthly intramuscular injections during the RSV season and is costly [208], which limits its use in many settings and in fewer than 5% of infants in well‐resourced countries. To improve feasibility, newer long‐acting antibodies have been engineered. Nirsevimab, a fully human anti‐RSV mAb containing Fc YTE mutations that extend half‐life by enhancing neonatal Fc receptor binding, persists for roughly 70–90 days and can provide protection for an entire RSV season after a single preseason dose [207, 209]. In Phase III trials, one injection reduced RSV‐related hospitalizations by about 71–78% in healthy infants compared with placebo [210, 211], leading to its approval in 2023 and incorporation into infant immunization programs as a universal passive immunization strategy for the first RSV season [212, 213, 214]. This success highlights how Fc engineering can markedly improve antibody prophylaxis by reducing dosing frequency and extending the window of protection [209, 215].

A similar approach was applied during the COVID‐19 pandemic with the antibody cocktail tixagevimab/cilgavimab (Evusheld), which was developed for pre‐exposure prophylaxis in people who respond poorly to vaccination, including immunocompromised patients. The two noncompeting antispike antibodies were engineered with YTE Fc modifications to prolong half‐life for approximately 3–4 months and to reduce Fc effector activity that might contribute to ADE [216, 217, 218, 219, 220]. In a placebo‐controlled trial, a single intramuscular administration reduced symptomatic COVID‐19 by 82% over 6 months in high‐risk, unvaccinated individuals [221], making it an important protective option when vaccine responses were inadequate. Yet this success also exposed a major limitation of mAbs: viral evolution can rapidly undermine their activity. As SARS‐CoV‐2 accumulated mutations, especially in Omicron subvariants [222, 223], spike alterations prevented binding and rendered Evusheld ineffective by late 2022 [224]. This experience underscores a central challenge of mAb prophylaxis, namely, immune escape, and has driven interest in bnAbs and multiantibody cocktails that target conserved epitopes. Such strategies are also being pursued for influenza, where HA stem‐directed bnAbs show cross‐strain activity, and for HIV, where combinations of bnAbs are being tested because single antibodies such as VRC01 protect only against susceptible strains [225, 226, 227, 228].

Beyond prophylaxis, mAbs have also proven valuable as early therapeutics for acute viral infections, where timing is critical. Their greatest benefit is usually seen when administered before extensive viral replication and tissue injury occur, as demonstrated in Ebola and COVID‐19, where patients with lower viral loads and milder disease derived the most benefit [229, 230]. During the 2018–2019 Ebola outbreak in the Democratic Republic of the Congo, the antibody therapies REGN‐EB3 and mAb114 significantly improved survival compared with ZMapp, with lower mortality rates and better outcomes when administered early [7, 231, 232]. REGN‐EB3 (Inmazeb) is a combination of three mAbs—atoltivimab, maftivimab, and odesivimab—that target the Ebola glycoprotein, whereas mAb114, later known as ansuvimab, is a single antibody derived from an Ebola survivor (a human monoclonal IgG1) [233, 234]. ZMapp is an older cocktail of three chimeric mAbs (c13C6, c2G4, and c4G7) engineered with mouse variable regions for Ebola glycoprotein recognition and human constant regions to reduce immunogenicity and produced through transient expression in tobacco plants [235]. REGN‐EB3 (Inmazeb) and ansuvimab (Ebanga) were approved in 2020 and are now stockpiled for Ebola outbreaks [234, 236]. Similarly, several anti‐SARS‐CoV‐2 mAbs developed in 2020–2021, including bamlanivimab, casirivimab/imdevimab, and sotrovimab, reduced hospitalization and death when used soon after symptom onset in high‐risk outpatients [237, 238, 239, 240]. Their (mAbs) main limitations are cost, injection‐based delivery, limited accessibility in resource‐poor settings, and the risk of resistance when viruses mutate under antibody pressure. For this reason, combination mAbs or pairing antibodies with DAAs are increasingly favored to improve breadth, durability, and resistance prevention.

#### 3.2.1 Pre‐Exposure Versus Postexposure Prophylaxis (PrEP Versus PEP)

mAbs can be used both as pre‐exposure prophylaxis (given before potential exposure, to high‐risk individuals or during high‐transmission seasons) and as postexposure prophylaxis (given immediately after a known exposure, to prevent infection or disease). In the context of COVID‐19, for example, the Regeneron antibody cocktail (casirivimab plus imdevimab) was tested for postexposure prophylaxis in household contacts: a single subcutaneous dose reduced the risk of developing symptomatic COVID‐19 by 81% in those who had a household member with COVID [241, 242]. For other viruses like rabies, postexposure prophylaxis is standard care: after a suspected rabid animal bite, prompt administration of rabies immunoglobulin (passive antibodies) around the wound, together with active vaccination, is nearly 100% effective at preventing the fatal infection. Recently, human mAb cocktails (e.g. two mAbs targeting nonoverlapping epitopes of rabies virus glycoprotein) have been developed as replacements for human rabies immunoglobulin, to ensure consistent quality and supply [243, 244]. Clinical trials indicate that these rabies mAb cocktails are safe and effective in neutralizing virus during the incubation period, offering a modern alternative to blood‐derived immunoglobulin for PEP. Another common example of antiviral PEP is the use of antivirals (drugs) for influenza: if an index case is diagnosed in a household or nursing home, oseltamivir (a NA inhibitor) can be given to exposed individuals for 7–10 days to prevent flu illness. Trials show that oseltamivir PEP can achieve ∼80–90% efficacy in preventing influenza transmission in households [245, 246]. Newer antivirals like baloxavir (a cap‐dependent endonuclease inhibitor) have similarly been shown to curb spread when given to contacts shortly after an index case is identified [247, 248, 249, 250]. Thus, monoclonals and small‐molecule antivirals are important tools for PEP in outbreak containment or high‐risk exposure scenarios (such as preventing influenza outbreak in long‐term care facilities). The timing of PEP is critical—it must be administered before the virus disseminates and causes disease, which for fast‐moving viruses means within days (or even hours) of exposure. When deployed swiftly, PEP can avert illness or substantially attenuate its severity.

#### 3.2.2 Programmatic and Pharmacologic Considerations

Prophylactic use of antivirals and antibodies requires careful consideration of PK, pharmacodynamics (PD), and adherence to dosing regimens. Antiretroviral pre‐exposure prophylaxis for HIV provides a telling example. Daily oral PrEP with emtricitabine/tenofovir (nucleoside/nucleotide reverse transcriptase inhibitors) combination antiretroviral medication has been shown to be highly effective (approaching 99% risk reduction in adherent individuals) [251, 252]. However, the real‐world effectiveness of oral PrEP is tightly linked to adherence—efficacy drops markedly if doses are missed [253]. Long‐acting injectable PrEP was developed to overcome the adherence challenge: Phase III trials of injectable cabotegravir (integrase inhibitor) given every 8 weeks showed it was up to 88% more effective than daily oral PrEP, largely because drug levels were maintained without user action, reducing dependence on daily adherence [254, 255]. The cabotegravir depot achieves sustained plasma and tissue levels, but it also raises new considerations: the long pharmacologic “tail” after injections (drug lingering for a year or more) can lead to sub‐therapeutic levels if a dose is missed, potentially selecting for resistant HIV if infection occurs. Programmatically, ensuring that individuals return for maintenance injections (or providing oral cover during the tail) is essential. For mAbs, PK/PD optimization has centered on extending half‐life to reduce dosing frequency, as discussed for nirsevimab and Evusheld. Extending mAb half‐life (via Fc engineering) not only improves convenience but also can ensure more consistent protective titers during high‐risk periods.

#### 3.2.3 Public‐Health Prophylaxis Measures

Beyond individual prophylaxis, there are broad public‐health strategies aimed at preventing viral infections community‐wide. Foremost among these is vaccination at the population level: mass vaccination campaigns have eradicated or curtailed multiple viral diseases. For example, the global polio eradication initiative has relied on coordinated oral polio vaccine campaigns reaching billions of children, pushing wild polio to the brink of extinction. Measles and rubella elimination programs use nationwide catch‐up immunization campaigns to close immunity gaps. Such campaigns are resource‐intensive but highly effective in raising herd immunity rapidly and uniformly. Ring vaccination is another strategy that has seen success against viruses like Ebola. In the 2014–2016 West African Ebola outbreak and subsequent outbreaks in the DRC, ring vaccination (vaccinating all contacts and contacts‐of‐contacts of each case) using a live recombinant vesicular stomatitis virus‐vectored Ebola vaccine helped break chains of transmission [256, 257, 258, 259, 260, 261]. By conferring immunity to those most likely to be exposed next, ring vaccination can contain an outbreak even when case numbers are still in the low hundreds. This approach was informed by smallpox eradication, where ring vaccination around cases ultimately proved more practical than mass vaccination once case numbers dwindled. Another pillar of prophylaxis for vector‐borne viral diseases is vector control. For arboviruses like dengue, Zika, and chikungunya (transmitted by Aedes mosquitoes), reducing or modifying mosquito populations is a form of prophylaxis at the community level. Traditional methods include insecticide spraying and eliminating breeding sites, but innovative biological interventions have emerged. Notably, the release of Wolbachia‐infected mosquitoes—which leads to mosquitoes with reduced capacity to transmit dengue virus—has shown remarkable impact. A cluster‐randomized trial in Indonesia demonstrated a 77% reduction in dengue incidence in areas seeded with Wolbachia‐carrying Aedes mosquitoes, compared with control areas [262, 263]. This indicates that vector control can significantly lower viral infection rates without a human vaccine. Other vector control methods, such as engineered sterile male mosquitoes or gene drives, are being tested to suppress mosquito populations and thereby human infections [264, 265]. These environmental prophylaxis measures are especially important for viruses (dengue, West Nile, yellow fever) where vaccines are absent or only partially protective.

Passive immunization programs are also employed as public‐health measures for certain exposures. For example, health authorities maintain stockpiles of immune globulin for postexposure prophylaxis in high‐risk situations: administering polyclonal immunoglobulin to susceptible individuals can prevent disease or attenuate illness. Measles exposure in an immunocompromised person is a case in point—if a nonimmune cancer patient is exposed to measles, they can receive intravenous immunoglobulin within 6 days to confer immediate antibodies and prevent severe measles [266, 267, 268]. Similarly, hepatitis A immunoglobulin is recommended after known exposure in older or immune‐suppressed patients who have not been vaccinated [269, 270]. These passive immunization interventions are targeted, last‐resort measures to protect those who cannot be actively immunized or where vaccine‐induced immunity would take too long to develop. With the advent of mAbs, some passive immunization programs are shifting to recombinant products.

## 4 Therapeutic Agents

The landscape of antiviral therapy has expanded significantly in the past decade, with diverse therapeutic strategies targeting either viral components or host factors. In this section, we discuss major classes of therapeutic agents against human viral diseases, including their mechanisms of action, representative examples, and key considerations such as PK, resistance, and combination use.

### 4.1 Therapeutic Vaccines and Combination Immunotherapies

Unlike prophylactic vaccines, which are administered before infection to prevent viral acquisition, therapeutic vaccines are given after infection to restore, broaden, or redirect antiviral immunity. Their principal objective is generally to induce durable virus‐specific CD4^+^ and CD8^+^ T‐cell responses capable of eliminating infected or transformed cells, reducing persistent viral reservoirs, or maintaining viral control without continuous treatment [271, 272, 273, 274]. Accordingly, therapeutic vaccines often target conserved internal viral proteins or antigens that remain expressed in persistently infected or virus‐transformed cells rather than relying primarily on nAbs against surface proteins. Candidate platforms include synthetic peptides, recombinant proteins, DNA and mRNA vaccines, viral vectors, dendritic‐cell vaccines, and heterologous prime‐boost regimens [275, 276, 277].

In chronic hepatitis B virus infection, therapeutic vaccines are being developed to restore functionally impaired HBV‐specific T‐cell responses and promote functional cure, commonly defined by sustained loss of hepatitis B surface antigen. Candidates such as GS‐4774 and BRII‐179 have demonstrated the ability to induce or enhance HBV‐specific immune responses, although vaccination alone has generally produced limited rates of surface‐antigen clearance [278, 279, 280, 281]. Current strategies therefore combine therapeutic vaccination with nucleos(t)ide analog therapy, antigen‐lowering agents, innate immune agonists, or other immunomodulators to reduce viral antigen burden before immune reactivation. In HIV infection, therapeutic vaccines based on conserved or protective T‐cell immunogens aim to strengthen cytotoxic responses against infected cells, restrict viral diversity, and delay viral rebound after analytical treatment interruption [282, 283]. Although several candidates have generated measurable HIV‐specific T‐cell responses, durable control without ART has been inconsistent, supporting combination approaches involving bnAbs, latency‐reversing agents, or reservoir‐targeting interventions.

Therapeutic vaccination is also being investigated for persistent high‐risk HPV infection and HPV‐associated premalignant lesions or cancers. In contrast to licensed prophylactic HPV vaccines based on the L1 capsid protein, therapeutic candidates commonly target the constitutively expressed E6 and E7 oncoproteins to promote cytotoxic elimination of infected or transformed cells [284]. The synthetic DNA vaccine VGX‐3100 provided clinical proof of concept by promoting histopathological regression and viral clearance in some patients with HPV16‐ or HPV18‐associated cervical intraepithelial neoplasia, although therapeutic HPV vaccination remains investigational and does not replace prophylactic vaccination or cervical screening [285].

The efficacy of therapeutic vaccines may be constrained by high antigen burden, viral latency, immune exhaustion, suppressive tissue microenvironments, and insufficient trafficking of vaccine‐induced T cells to infected tissues. Consequently, therapeutic vaccination is increasingly being integrated with cellular immunotherapies and immune‐checkpoint modulation [286]. Adoptive transfer of VST cells, including T‐cell receptor‐engineered or chimeric antigen receptor (CAR) T cells, can increase the number and functional potency of antiviral effector cells, whereas blockade of inhibitory pathways such as PD‐1–PD‐L1 may partially restore exhausted T‐cell activity [287, 288, 289, 290]. However, checkpoint inhibition in chronic viral infection requires caution because nonspecific immune activation can cause hepatitis, tissue inflammation, or other immune‐related toxicities. Therapeutic vaccines are therefore most likely to achieve meaningful clinical benefit as components of rational combination regimens that simultaneously reduce viral or antigen burden, reverse immune dysfunction, and enhance targeted clearance of infected cells.

### 4.2 Small‐Molecule Antivirals

#### 4.2.1 Direct‐Acting Antivirals (DAAs)

Most approved antivirals are DAAs that target specific viral enzymes or proteins (Table 1), often with high potency but narrow spectrum [291, 292]. A prime example is polymerase inhibitors, typically nucleoside or nucleotide analogs that mimic natural nucleotides to terminate viral genome replication. For instance, remdesivir and molnupiravir are nucleoside analogs (Nas) that inhibit the RNA‐dependent RNA polymerase of RNA viruses. Remdesivir was developed as a broad‐spectrum Na and advanced clinically for Ebola virus disease, and molnupiravir for Venezuelan equine encephalitis virus, but both were repurposed during COVID‐19 as broad‐spectrum antivirals [293, 294, 295, 296] (Figure 4). Molnupiravir induces lethal mutagenesis in viral RNA, while remdesivir causes chain termination in nascent viral genomes [297, 298, 299, 300]. Another notable polymerase inhibitor is favipiravir, which is orally bioavailable and has shown activity against influenza and other RNA viruses by a similar mutagenesis mechanism [301, 302]. For DNA viruses like HBV and herpesviruses, approved nucleos(t)ide analogs (e.g., entecavir, tenofovir for HBV; acyclovir for herpes) target viral DNA polymerases.

**TABLE 1:** Chemical structures, molecular targets, and regulatory status of small‐molecule antivirals.

Drug/class and target/use/regulatory status	2D structure/formula/PubChem CID	Drug/class and target/use/regulatory status	2D structure/formula/PubChem CID
Remdesivir Nucleotide prodrug; viral RdRp inhibitor; SARS‐CoV‐2; US FDA‐approved (Veklury); intravenous treatment	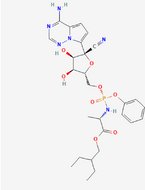 C_27_H_35_N_6_O_8_P; CID 121304016	Entecavir Guanosine analogue; HBV reverse transcriptase/DNA polymerase; chronic HBV; US FDA‐approved oral antiviral	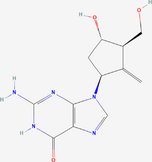 C_12_H_15_N_5_O_3_; CID 135398508
Tenofovir disoproxil (TDF active prodrug moiety); acyclic nucleotide phosphonate prodrug; reverse transcriptase inhibitor; HIV‐1 and chronic HBV; US FDA‐approved in fumarate‐containing products	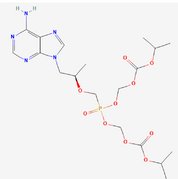 C_19_H_30_N_5_O_10_P; CID 5481350	Tenofovir alafenamide (TAF) Intracellular tenofovir prodrug; reverse transcriptase inhibitor; HIV‐1 and chronic HBV; US FDA‐approved in fumarate‐containing products	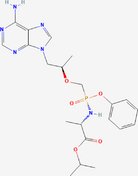 C_21_H_29_N_6_O_5_P; CID 9574768
Acyclovir Guanosine analogue; viral thymidine‐kinase activation and DNA‐polymerase inhibition; HSV‐1/2 and VZV; US FDA‐approved antiviral	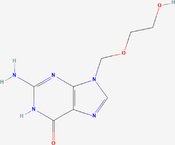 C_8_H_11_N_5_O_3_; CID 135398513	Lopinavir HIV‐1 protease inhibitor; HIV‐1; US FDA‐approved only in ritonavir‐boosted products not approved for COVID‐19	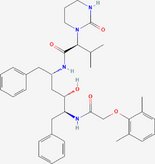 C_37_H_48_N_4_O_5_; CID 92727
Ritonavir HIV protease inhibitor; potent CYP3A inhibitor used as a booster; HIV‐1; boosts lopinavir and nirmatrelvir; US FDA‐approved; antiviral and pharmacokinetic enhancer. No intrinsic anti‐SARS‐CoV‐2 activity at booster dose	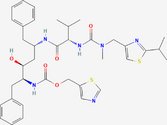 C_37_H_48_N_6_O_5_S_2_; CID 392622	Atazanavir HIV‐1 protease inhibitor; HIV‐1; US FDA‐approved	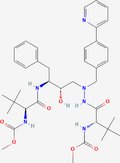 C_38_H_52_N_6_O_7_; CID 148192
Glecaprevir HCV NS3/4A protease inhibitor; chronic HCV; US FDA‐approved as the fixed‐dose glecaprevir/pibrentasvir combination (Mavyret)	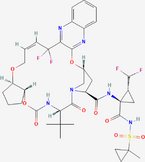 C_38_H_46_F_4_N_6_O_9_S; CID 66828839	Pibrentasvir HCV NS5A inhibitor; chronic HCV; US FDA‐approved as the fixed‐dose glecaprevir/pibrentasvir combination (Mavyret)	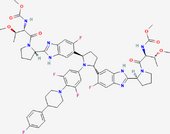 C_57_H_65_F_5_N_10_O_8_; CID 58031952
Sofosbuvir Nucleotide prodrug; HCV NS5B polymerase inhibitor; chronic HCV; US FDA‐approved alone and in fixed‐dose DAA combinations	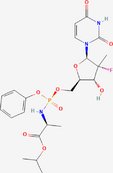 C_22_H_29_FN_3_O_9_P; CID 45375808	Velpatasvir HCV NS5A inhibitor; chronic HCV; US FDA‐approved with sofosbuvir (Epclusa) and in sofosbuvir/velpatasvir/voxilaprevir (Vosevi)	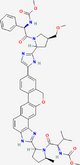 C_49_H_54_N_8_O_8_; CID 67683363
Voxilaprevir HCV NS3/4A protease inhibitor; chronic HCV after selected prior DAA failure; US FDA‐approved in the fixed‐dose Vosevi rescue regimen	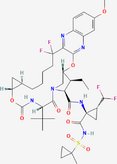 C_40_H_52_F_4_N_6_O_9_S; CID 89921642	Nirmatrelvir Covalent reversible SARS‐CoV‐2 Mpro/3CLpro inhibitor; COVID‐19; US FDA‐approved for eligible high‐risk adults only as nirmatrelvir/ritonavir (Paxlovid); pediatric use remains under EUA	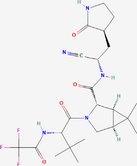 C_23_H_32_F_3_N_5_O_4_; CID 155903259
Maraviroc Host CCR5 antagonist; blocks viral entry; CCR5‐tropic HIV‐1; US FDA‐approved entry inhibitor; tropism testing is required before use	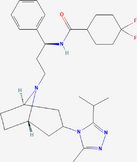 C_29_H_41_F_2_N_5_O; CID 3002977	Oseltamivir Oral prodrug; influenza neuraminidase inhibitor; influenza A and B; US FDA‐approved; marketed as the phosphate salt	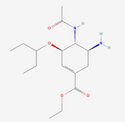 C_16_H_28_N_2_O_4_; CID 65028
Zanamivir Influenza neuraminidase inhibitor; influenza A and B; US FDA‐approved inhaled antiviral (Relenza)	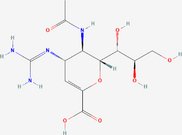 C_12_H_20_N_4_O_7_; CID 60855	Baloxavir marboxil Prodrug; influenza cap‐dependent endonuclease inhibitor; influenza A and B; US FDA‐approved single‐dose oral antiviral (Xofluza)	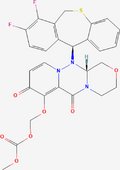 C_27_H_23_F_2_N_3_O_7_S; CID 124081896
Lenacapavir Multistage HIV‐1 capsid inhibitor; Multidrug‐resistant HIV‐1 treatment; HIV‐1 PrEP; US FDA‐approved as Sunlenca for treatment (2022) and Yeztugo for PrEP (2025); 6‐monthly maintenance injection after initiation	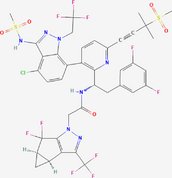 C_39_H_32_C_l_F_10_N_7_O_5_S_2_; CID 133082658	Ribavirin Nucleoside analogue with multiple antiviral mechanisms; selected RSV and HCV indications; other uses vary by jurisdiction; US FDA‐approved products exist, although contemporary use is limited and indication/formulation specific	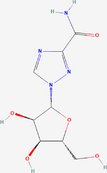 C_8_H_12_N_4_O_5_; CID 37542
Zidovudine (AZT) Thymidine analogue; HIV reverse‐transcriptase inhibitor; HIV‐1; US FDA‐approved nucleoside reverse‐transcriptase inhibitor; now used in combination regimens	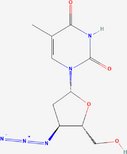 C_10_H_13_N_5_O_4_; CID 35370	Cobicistat CYP3A inhibitor; pharmacokinetic enhancer without intrinsic antiviral activity; boosts selected antiretrovirals; US FDA‐approved only as a pharmacoenhancer in HIV products; should not be described as an antiviral itself	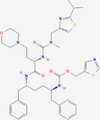 C_40_H_53_N_7_O_5_S_2_; CID 25151504
Molnupiravir Prodrug of N4‐hydroxycytidine; RdRp‐mediated lethal mutagenesis; COVID‐19; US FDA EUA‐authorized for selected high‐risk adults; not US FDA‐approved; the COVID‐19 drug‐EUA declaration is scheduled to terminate June 29, 2027	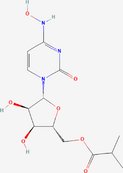 C_13_H_19_N_3_O_7_; CID 145996610	Favipiravir (T‐705) Purine nucleobase analogue; viral RdRp inhibition/mutagenesis; novel or re‐emerging influenza; investigational for other RNA viruses; Not US FDA‐approved. Restricted Japanese approval/stockpile use; not routinely marketed in Japan	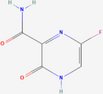 C_5_H_4_FN_3_O_2_; CID 492405
Nitazoxanide Host/viral‐protein‐processing effects proposed; mechanism is context dependent; investigational antiviral repurposing; US FDA‐approved as an antiprotozoal, not as an antiviral; antiviral use remains investigational	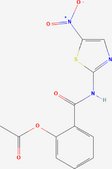 C_12_H_9_N_3_O_5_S; CID 41684	Boceprevir First‐generation HCV NS3/4A protease inhibitor; Chronic HCV (historical); formerly US FDA‐approved; US marketing discontinued and superseded by modern DAA combinations	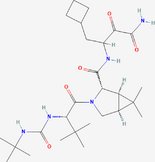 C_27_H_45_N_5_O_5_; CID 10324367
Telaprevir First‐generation HCV NS3/4A protease inhibitor; chronic HCV (historical); Formerly US FDA‐approved; US marketing discontinued and superseded by modern DAA combinations	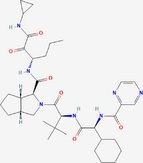 C_36_H_53_N_7_O_6_; CID 3010818	ALG‐000184 CAM‐E (Class II) HBV capsid assembly modulator; prodrug of ALG‐001075; chronic HBV; investigational; active, not recruiting Phase IIB‐SUPREME study (NCT06963710; not US FDA‐approved	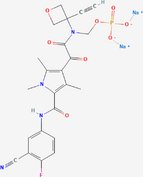 C_23_H_20_FN_4_Na_2_O_8_P; CID 172677403
Canocapavir (ZM‐H1505R) HBV core/capsid assembly modulator; chronic HBV in combination with nucleos(t)ide analogues; investigational; registered Phase III study NCT07095855; Not US FDA‐approved	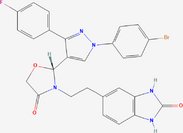 C_27_H_21_BrFN_5_O_3_; CID 137477479	Docosanol Long‐chain aliphatic alcohol; interferes with membrane fusion/entry; recurrent herpes labialis; US FDA‐approved OTC topical antiviral (Abreva)	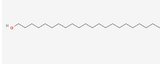 C_22_H_46_O; CID 12620

*Note*: Structures are shown as the PubChem parent/active moiety [346] unless a prodrug is explicitly named; marketed salts and excipients are not drawn. US FDA approval refers only to the stated product/indication and should not be extrapolated to investigational antiviral uses. EUA is legally distinct from US FDA approval. Trial status can change; Last accessed in August 2026.

*Abbreviations*: 3CLpro, 3C‐like protease; AZT, 3′‐azido‐3′‐deoxythymidine; CAM‐E, Class II capsid assembly modulator that promotes the formation of empty capsids; CCR5, C–C chemokine receptor Type 5; CID, PubChem compound identifier; COVID‐19, coronavirus disease 2019; CYP3A, cytochrome P450 3A; DAA, direct‐acting antiviral; DNA, deoxyribonucleic acid; EUA, Emergency Use Authorization; US FDA, US Food and Drug Administration; HBV, hepatitis B virus; HCV, hepatitis C virus; HIV‐1, human immunodeficiency virus Type 1; HSV‐1/2, herpes simplex virus Types 1 and 2; Mpro, main protease; NCT, ClinicalTrials.gov study identifier; NS3/4A, hepatitis C virus nonstructural protein 3/4A protease; NS5A, hepatitis C virus nonstructural protein 5A; NS5B, hepatitis C virus nonstructural protein 5B RNA‐dependent RNA polymerase; OTC, over‐the‐counter; PrEP, pre‐exposure prophylaxis; RdRp, RNA‐dependent RNA polymerase; RNA, ribonucleic acid; RSV, respiratory syncytial virus; SARS‐CoV‐2, severe acute respiratory syndrome coronavirus 2; TAF, tenofovir alafenamide; TDF, tenofovir disoproxil fumarate; VZV, varicella‐zoster virus.

**FIGURE 4:**
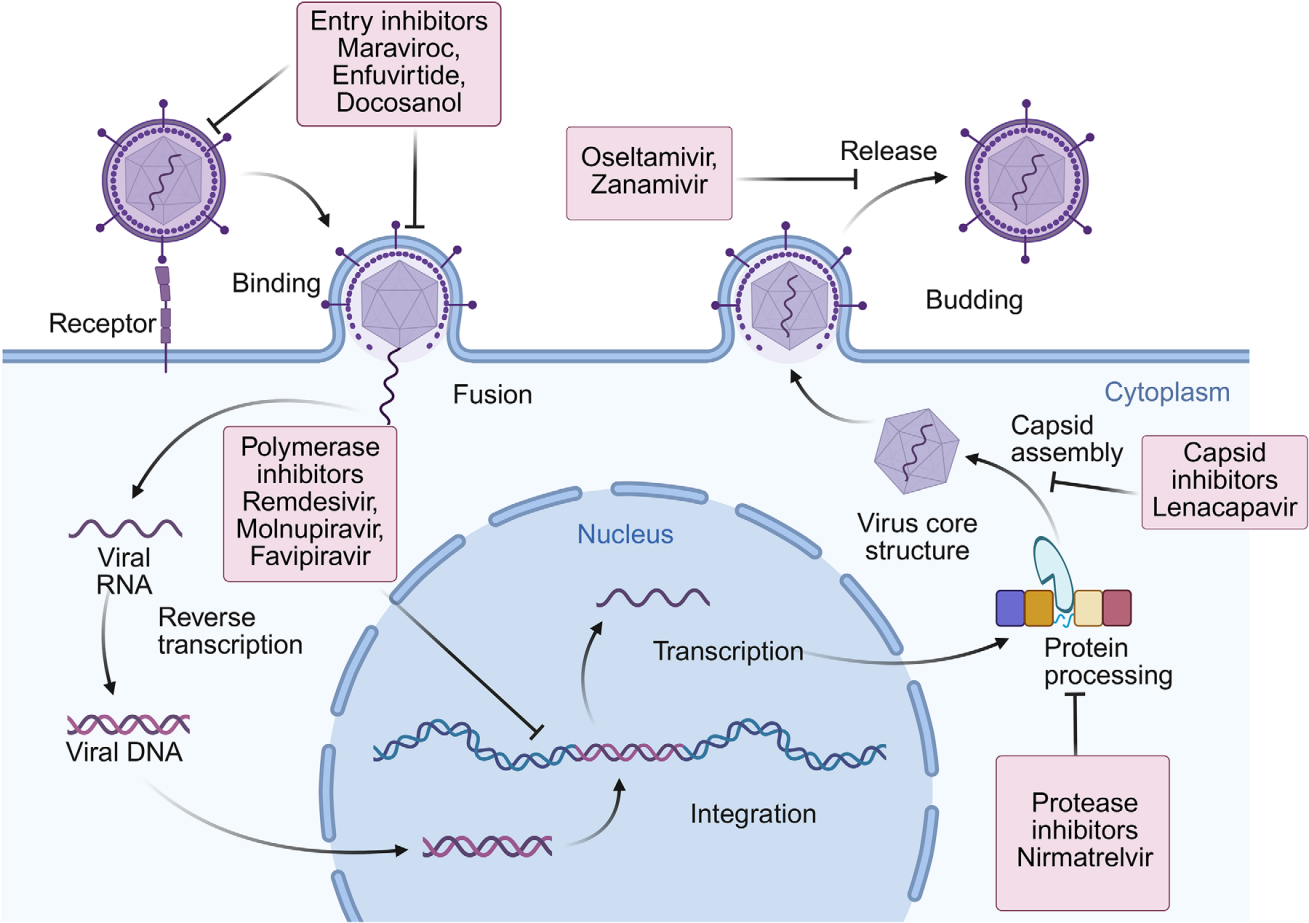
Antiviral drugs and their major molecular targets during viral infection. Schematic illustration highlighting the stages of viral infection targeted by antiviral agents. Entry inhibitors, including maraviroc, enfuvirtide, and docosanol, prevent viral attachment, receptor engagement, or membrane fusion. Polymerase inhibitors, such as remdesivir, molnupiravir, and favipiravir, suppress viral genome replication by inhibiting viral polymerase activity. Protease inhibitor, for example, nirmatrelvir, block proteolytic processing of viral polyproteins required for maturation. The capsid inhibitor lenacapavir interferes with capsid assembly and stability. Neuraminidase inhibitors oseltamivir and zanamivir inhibit viral particle release from infected cells.

Protease inhibitors are another cornerstone of DAAs. In HIV therapy, protease inhibitors lopinavir and atazanavir has revolutionized the treatment by blocking the viral protease needed to process HIV polyproteins [303, 304]. In HCV therapy, current all‐oral regimens incorporate second‐generation NS3/4A protease inhibitors as components of potent, pangenotypic combinations [305]. Glecaprevir is coformulated with the NS5A inhibitor pibrentasvir and is widely recommended for treatment‐naïve patients with chronic HCV infection [306, 307]. Voxilaprevir is combined with the NS5B polymerase inhibitor sofosbuvir and the NS5A inhibitor velpatasvir and is used primarily as a rescue regimen following failure of previous sofosbuvir‐based DAA therapy [308]. These contemporary combinations have largely replaced first‐generation protease inhibitors such as boceprevir and telaprevir, which are no longer used in current clinical practice. Recently, nirmatrelvir—a 3CL protease inhibitor—was rapidly developed for SARS‐CoV‐2 as a part of the oral combination Paxlovid [98, 309, 310]. Protease inhibitors often require coadministration of a low‐dose ritonavir (a potent CYP3A4 inhibitor) to boost their plasma levels. This PK boosting allows once‐daily dosing and higher drug exposure [311], but comes with a risk of drug–drug interactions due to ritonavir's broad effect on liver enzymes. Entry inhibitors prevent viruses from entering host cells. One class are receptor antagonists: for example, maraviroc is a CCR5 antagonist that blocks HIV from binding the CCR5 coreceptor on T cells. Fusion inhibitors like enfuvirtide (a peptide drug) bind viral envelope proteins to prevent fusion of viral and cellular membranes. Enfuvirtide was the first HIV fusion inhibitor, and similar fusion‐blocking strategies are being explored for other viruses (e.g., small molecules targeting the RSV fusion protein) [312]. Another entry inhibitor class are neutralizing attachment inhibitors; an example is docosanol (an OTC cream for HSV), which intercalates into cell membranes to block HSV entry [313].

For influenza, the prototypical DAAs are NA inhibitors such as oseltamivir (Tamiflu) and zanamivir. These prevent release of new influenza virions from infected cells by inhibiting the viral NA enzyme. They have been standard of care for influenza treatment for over two decades. Additionally, a newer influenza DAA, baloxavir marboxil, targets the viral cap‐dependent endonuclease (a component of the polymerase), representing a different mechanism to inhibit viral RNA synthesis. Emerging DAA classes include capsid assembly inhibitors. These agents interfere with formation of the viral capsid or core, thus blocking the viral life cycle at a postreplication stage. A milestone in this category was the approval of lenacapavir for multidrug‐resistant HIV in 2022 [106, 314]. Lenacapavir binds to the HIV capsid protein (p24), stabilizing capsid structures and impairing essential processes like nuclear import of viral DNA and assembly of new virions [315, 316]. It is long‐acting (dosed bi‐annually) and, as the first‐in‐class capsid inhibitor, retains efficacy against HIV strains resistant to other drug classes. In HBV, several capsid assembly modulators are in clinical trials; by targeting the HBV core protein, they aim to prevent proper capsid formation and deplete the pool of cccDNA, a critical viral replicative intermediate.

#### 4.2.2 Broad‐Spectrum Candidates

Traditional DAAs are often virus‐specific, but broad‐spectrum antivirals are being pursued to address emergent pathogens [291]. Broad‐spectrum Nas like ribavirin have long been known to inhibit a range of RNA and DNA viruses, although often with only moderate efficacy. Discovered in the 1970s, ribavirin introduced the concept of a pan‐viral antiviral and has been used often in combination therapy against RSV, Lassa fever, HCV, and others [317, 318]. Favipiravir (T‐705), as noted, has activity against multiple RNA viruses by inducing error catastrophe [319]. These broad‐spectrum NAs hold appeal for novel outbreaks, as seen when remdesivir and molnupiravir were swiftly repurposed for COVID‐19 [320].

Another approach to broad‐spectrum therapy is targeting host molecules co‐opted by many viruses. Some small molecules originally developed for other diseases have shown broad antiviral effects by modulating host pathways. For example, nitazoxanide, an antiparasitic drug, has demonstrated modest antiviral activity against influenza, coronaviruses, paramyxovirus, and hepatitis viruses by broadly affecting processes like glycoprotein maturation [321, 322, 323, 324]. In a flu trial, nitazoxanide shortened symptom duration [325], and a pilot study in COVID‐19 hinted at accelerated viral clearance and reduced inflammatory markers [326]. While not a potent DAA per se, nitazoxanide's host‐targeted mechanism (thought to involve IFN pathway priming and viral protein processing blockade) gives it pan‐viral potential. Other host‐targeted small molecules with broad antiviral properties are being investigated (e.g., inhibitors of cyclophilin, which is exploited by HIV, HCV, and coronaviruses for protein folding [327].

#### 4.2.3 Pharmacokinetics, Resistance, and Drug Interactions

Small‐molecule antivirals must achieve sufficient levels at infection sites without undue toxicity. Oral bioavailability and half‐life are critical; for instance, molnupiravir's oral dosing made it convenient for early COVID outpatient therapy, whereas remdesivir requires intravenous administration. PK boosting with ritonavir (as used in HIV and now in Paxlovid for COVID‐19) exemplifies how PK can be manipulated: ritonavir's inhibition of CYP3A4 dramatically raises levels of coadministered antivirals, enabling lower or less frequent dosing [328, 329]. However, this strategy necessitates careful management of drug–drug interactions. Ritonavir and the similar booster cobicistat can interact with a wide array of medications (statins, anticoagulants, etc.), requiring clinicians to review concomitant drugs to avoid adverse effects [330, 331].

Exposure is further modified by age, body size, pregnancy, inflammation, renal or hepatic impairment, genetic variation in metabolizing enzymes or transporters, and adherence. Renally cleared antivirals can accumulate when glomerular filtration is reduced, whereas hepatic dysfunction can change biotransformation, biliary transport, and protein binding [332, 333, 334, 335]. Dose selection should therefore be based on route‐appropriate PK data, organ function, and, when available, exposure–response modeling rather than a uniform dose for all patients. Infection‐site PK is especially important in sanctuary sites such as the central nervous system, genital tract, lung epithelial lining fluid, lymphoid tissue, and intracellular reservoirs, where subtherapeutic exposure can permit continued replication despite apparently adequate plasma levels [336, 337, 338]. Therapeutic drug monitoring is not routine for every antiviral but can be useful when absorption, organ dysfunction, adherence, or a clinically important PK interaction is uncertain [339].

Antiviral resistance is a perpetual concern. RNA viruses, in particular, mutate rapidly under drug pressure. Monotherapy with DAAs often leads to the emergence of resistant variants, as observed historically with single AZT therapy in HIV or with early HCV protease inhibitor monotherapy. Even short courses for acute infections carry risk: SARS‐CoV‐2 variants with reduced susceptibility to nirmatrelvir and remdesivir have already been documented [340, 341]. To mitigate resistance, combination therapy is a guiding principle. Using drugs with different targets and resistance profiles can produce synergistic suppression of the virus and prevent any one mutation from conferring high‐level resistance [94, 342, 343]. This multipronged approach was key to the success of modern HIV (3‐drug ART) and HCV regimens (2–3 DAAs), which achieve durable suppression or cure while greatly reducing the likelihood of resistance [344, 345]. The trade‐off of combination regimens is the increased complexity and potential for PK interactions among the agents, underscoring the need for regimen optimization and therapeutic drug monitoring in some cases.

Drug interactions can alter antiviral exposure during absorption, metabolism, transport, or elimination. Mechanisms include changes in gastric pH or intestinal chelation; inhibition or induction of cytochrome P450 enzymes or uridine diphosphate glucuronosyltransferase pathways. PK boosting illustrates both benefit and risk: ritonavir or cobicistat can increase and prolong exposure to CYP3A substrates, but they may also substantially raise concentrations of comedications with narrow therapeutic indices. Conversely, strong enzyme inducers may lower antiviral exposure and cannot always be managed by dose spacing because induction persists after the inducer is stopped. Clinically important examples include interactions with selected antiarrhythmics, anticoagulants, anticonvulsants, immunosuppressants, rifamycins, statins, sedatives, and herbal products [328, 329, 330, 331].The overall regimen should be optimized jointly for antiviral potency, genetic barrier, exposure at the infection site, adherence feasibility, and interaction burden; these factors are interdependent because an unrecognized interaction or poorly tolerated regimen can reduce exposure and create conditions that favor resistance [344, 345].

### 4.3 Host‐Targeted Therapies

Host‐targeted antivirals (HTAs) are agents that modulate host cell factors essential for viral replication or pathogenesis, rather than directly attacking viral proteins. By targeting the host, these therapies offer two main potential advantages: a broader spectrum of activity since many viruses rely on similar host pathways and a higher barrier to viral resistance [292, 347]. Because the virus cannot easily escape by mutating a host dependency factor, HTAs can retain efficacy even as the virus evolves [94]. Indeed, in cell culture and animal studies, viruses show delayed or no resistance development to host‐targeted inhibitors (e.g., inhibitors of cellular kinases or α‐glucosidase) compared with classic DAAs [291]. Clinically, certain host‐directed approaches (e.g., cyclophilin inhibitors) demonstrated slower emergence of resistance in hepatitis C patients than seen with DAAs.

#### 4.3.1 Targeting Host Factors Required for Viral Replication

Viruses rely on numerous host proteins and pathways to complete their life cycles, and recent high‐throughput genomic and proteomic studies have identified several host dependency factors that can be therapeutically targeted (Figure 5). Cyclophilin inhibitors are a prominent example: cyclophilin A, a host peptidyl‐prolyl isomerase involved in protein folding, is exploited by HIV and HCV, and nonimmunosuppressive cyclosporine derivatives such as alisporivir (Debio‐025) and SCY‐635 have shown broad antiviral activity by disrupting these interactions, with alisporivir reducing HCV viremia in clinical trials before a Phase III study was halted because of rare pancreatitis [348, 349, 350, 351, 352]. Host ER α‐glucosidase inhibitors such as celgosivir interfere with N‐linked glycosylation and have shown broad in vitro activity against flaviviruses, paramyxoviruses, and other viruses, although clinical benefit in dengue has been limited [353]. Heat shock protein inhibitors targeting HSP70 and HSP90 can impair replication of flaviviruses, rhabdoviruses, and coronaviruses, and in Zika and chikungunya models they reduced viral titers and inflammation [354, 355]. Kinase inhibitors also show promise: EGFR/ErbB signaling supports entry of HCV, CMV, and some coronaviruses, and approved inhibitors such as gefitinib and lapatinib suppressed HCV and SARS‐CoV‐2 in preclinical studies, while p38 MAP kinase inhibition reduced both dengue and Ebola replication and hyperinflammation [356, 357, 358, 359, 360, 361]. Viruses also hijack lipid metabolism, making cholesterol and fatty‐acid pathway modulators potential antivirals, although clinical utility remains uncertain.

**FIGURE 5:**
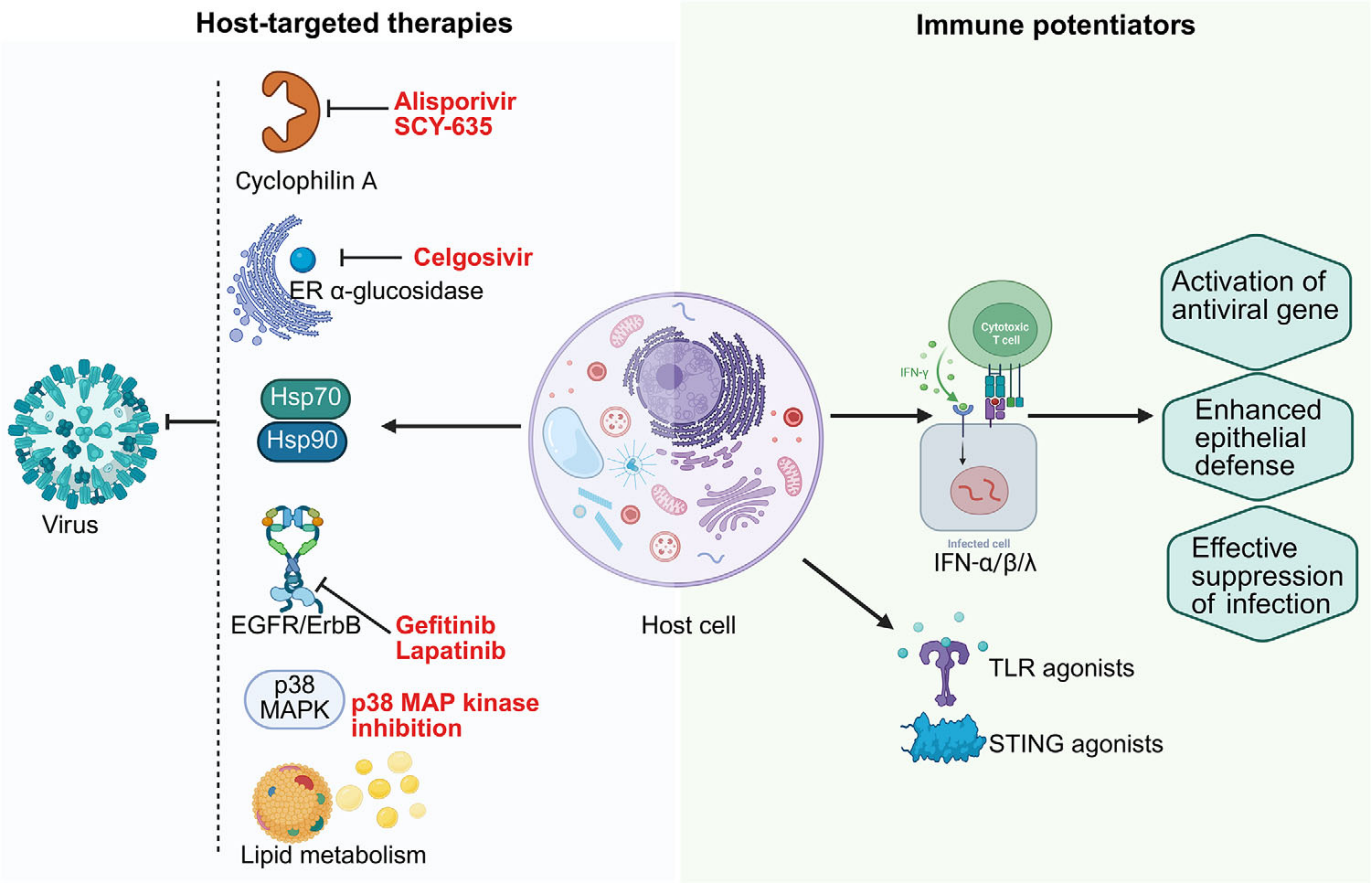
Host‐targeted therapies and immune potentiators with antiviral activity. Schematic representation of antiviral strategies that act on host pathways rather than directly on viral components. Host‐targeted therapies include inhibitors of cyclophilin A (alisporivir, SCY‐635), ER α‐glucosidase (celgosivir), Hsp70/Hsp90 chaperones, EGFR/ErbB signaling (gefitinib, lapatinib), p38 MAP kinase signaling, and lipid metabolism. Immune potentiators promote antiviral defense by enhancing innate and adaptive responses, including activation of antiviral genes, epithelial defense, and infection suppression through IFN‐α/β/λ signaling, TLR agonists, and STING agonists.

#### 4.3.2 Immune Potentiators

Another host‐directed strategy is to potentiate antiviral immunity by enhancing the host response to infection. Type I IFNs (IFN‐α/β) have long been used as antiviral agents, with pegylated IFN‐α once forming the backbone of hepatitis therapy and still used in some hepatitis B regimens, although systemic toxicity limits its use [362, 363]. IFN lambda (IFN‐λ) is a more targeted cytokine that acts mainly on epithelial cells through the IL‐28R receptor and may therefore provide antiviral benefit with fewer adverse effects; in a Phase III COVID‐19 trial, a single dose of pegylated IFN‐λ reduced hospitalization or death by about 50% and remained active across variants and in vaccinated individuals [364]. Because IFNs stimulate multiple host antiviral pathways, viral escape is less likely than with mAbs. Other immune potentiators, including TLR and STING agonists, are also under investigation.

HTAs can be limited by toxicity because they act on normal cellular functions, making a nontoxic antiviral dose difficult to achieve. For example, dasatinib, a Src/Abl kinase inhibitor, inhibits multiple viruses in vitro but caused immunosuppression at antiviral doses in animal models, undermining protection [365]. Although careful dosing and therapeutic windows can make these agents feasible, short treatment courses may be safer. In addition, mechanisms are often incompletely understood, as with nitazoxanide, and species differences can limit translation, exemplified by remdesivir's success in monkeys but failure in human Ebola [366].

### 4.4 Immunomodulators

Immunomodulators act by reshaping the host response to infection rather than directly targeting the virus, making them particularly useful when viral disease is driven by either excessive inflammation or immune exhaustion [367]. In severe acute infections, the major goal is to suppress harmful hyperinflammation while preserving enough antiviral activity to control the pathogen. Corticosteroids remain the best‐established example (Figure 6). In COVID‐19, the RECOVERY trial showed that dexamethasone (6 mg daily) reduced 28‐day mortality in hospitalized patients requiring oxygen or mechanical ventilation, and later studies confirmed that steroids improve survival in severe COVID‐19 lung disease, especially in patients with advanced respiratory support needs [368, 369, 370]. Their benefit reflects attenuation of cytokine storm and ARDS, whereas use in mild disease or before inflammatory escalation may impair viral clearance and worsen outcomes. This timing‐dependent effect has also been recognized in other viral pneumonias, reinforcing that steroids are most appropriate once hyperinflammation becomes clinically evident.

**FIGURE 6:**
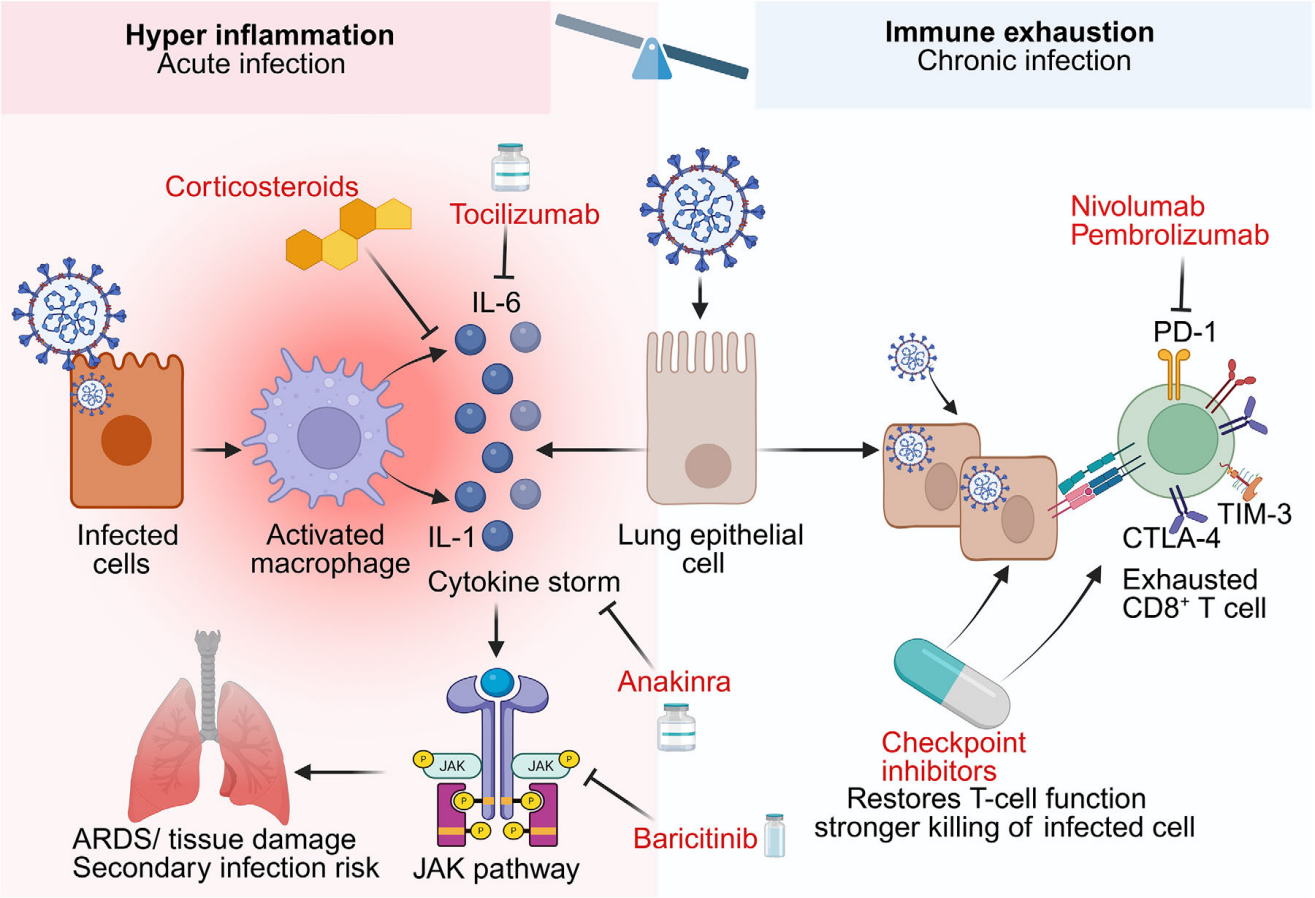
Immunomodulatory therapies for virus‐associated hyperinflammation and immune exhaustion. Schematic illustration of host immune responses during acute and chronic infection and the therapeutic agents used to modulate them. In acute infection, infected cells trigger macrophage activation and cytokine release, leading to hyperinflammation, cytokine storm, and downstream tissue damage. Corticosteroids, tocilizumab, anakinra, and baricitinib are shown as representative anti‐inflammatory or cytokine‐targeted therapies that attenuate this response, including inhibition of IL‐6, IL‐1, and JAK signaling. In chronic infection, persistent antigen exposure contributes to T‐cell exhaustion, characterized by impaired effector function and increased expression of immune checkpoint receptors such as PD‐1, CTLA‐4, and TIM‐3. Checkpoint inhibitors, including nivolumab and pembrolizumab, are depicted as agents that restore T‐cell activity and enhance killing of infected cells.

More targeted anti‐inflammatory agents have also been repurposed. IL‐6 pathway blockade with tocilizumab improved survival and recovery in hospitalized COVID‐19 patients with systemic inflammation and respiratory failure, and added benefit was observed when combined with steroids, including a modest absolute mortality reduction and shorter hospitalization [371, 372]. Similarly, the IL‐1 receptor antagonist anakinra has shown benefit in selected COVID‐19 patients with hyperferritinemia or macrophage‐activation features [373, 374, 375]. JAK inhibitors provide another host‐directed strategy by blocking intracellular signaling from multiple cytokines. Baricitinib, a JAK1/2 inhibitor, accelerated recovery when combined with remdesivir and reduced mortality on top of standard care, including steroids [376, 377]; meta‐analytic data also support a mortality benefit in COVID‐19. These drugs likely work by dampening broad inflammatory cascades, and baricitinib may also interfere with viral entry. However, JAK inhibitors and steroids together can increase susceptibility to secondary infections, so careful monitoring is essential.

At the opposite end of the spectrum, checkpoint inhibitors aim to reinvigorate exhausted T cells in chronic viral infections such as HIV, HBV, and HCV, where persistent antigen exposure drives PD‐1, CTLA‐4, TIM‐3, and related inhibitory pathways [378, 379, 380]. PD‐1 blockade with agents such as nivolumab and pembrolizumab has occasionally been associated with viral suppression or functional hepatitis cure in cancer patients with chronic HBV or HCV, but checkpoint inhibitors can also trigger HBV reactivation or hepatitis flares [381, 382, 383]. Early HBV trials of nivolumab showed transient boosts in HBV‐specific T‐cell responses and occasional HBsAg reductions [384], and ongoing studies are testing checkpoint blockade with antivirals or therapeutic vaccines. In HIV, similar strategies are being explored to support kick‐and‐kill approaches. Yet these interventions remain experimental because they can provoke autoimmune toxicity, immune reconstitution inflammatory syndrome, or hepatitis flares, especially in HBV where sudden immune activation can damage infected hepatocytes.

### 4.5 RNA‐Based and Gene‐Editing Antivirals

Advances in molecular medicine have enabled antiviral strategies that act at the genetic level, including siRNAs, antisense oligonucleotides (ASOs), and CRISPR‐based gene editing. siRNAs are ∼21‐nucleotide double‐stranded RNAs that use the RNAi/RISC machinery to cleave complementary viral mRNAs [385, 386], whereas ASOs are single‐stranded oligonucleotides that bind viral RNA and trigger RNase H‐mediated degradation or block translation [387]. Because unmodified nucleic acids are unstable and poorly taken up by cells, these molecules are extensively chemically optimized: ASOs often use gapmer designs with modified wings (e.g., 2′‐O‐methyl, 2′‐MOE, or locked nucleic acids) flanking a DNA gap, and siRNAs commonly contain 2′‐F or 2′‐O‐methyl substitutions plus phosphorothioate linkages to increase stability and reduce immunogenicity [388, 389, 390]. Targeting ligands such as GalNAc [391] can further direct oligonucleotides to hepatocytes. Delivery remains the main hurdle, but Lipid nanoparticles (LNPs) and inhaled formulations are expanding the reach of RNA antivirals. Clinically, this platform has already produced important examples: fomivirsen was the first antiviral ASO approved, for CMV retinitis; bepirovirsen, an HBV ASO, produced HBsAg clearance and undetectable HBV DNA in about 9–10% of chronic HBV patients after 24 weeks in Phase IIb, with higher responses in those starting with lower HBsAg, and is now in Phase III [392, 393, 394]. siRNA candidates such as JNJ‐3989 have also shown substantial HBV antigen suppression in combination regimens, while ALN‐RSV01 provided proof‐of‐concept antiviral activity against RSV, and experimental siRNAs against SARS‐CoV‐2 showed potent knockdown in animal models [395, 396]. Miravirsen, an ASO against host miR‐122, further demonstrated that host‐directed antisense therapy can reduce HCV, although DAAs ultimately superseded it [397, 398].

CRISPR‐based antivirals extend this genetic strategy by directly cutting viral DNA or RNA. Cas9 can target DNA viruses or integrated proviruses, making it attractive for HBV cccDNA, HPV, and HIV proviral DNA; in humanized mice, AAV‐delivered Cas9 plus long‐acting ART eliminated HIV in some animals, although translation to humans remains difficult [399]. Cas13, by contrast, targets RNA and is therefore well suited to RNA viruses such as influenza, coronaviruses, and HIV during active replication; the PAC‐MAN approach showed that pooled Cas13 guides could degrade conserved coronavirus sequences, including SARS‐CoV‐2, in human lung cells [400, 401]. The biggest barriers are delivery, off‐target effects, and immunogenicity. AAV vectors face payload limits and immune issues, while LNPs carrying Cas mRNA and guides offer a promising nonviral route and have already reached the clinic for noninfectious disease. Safety concerns remain significant: Cas9 can cut unintended genomic sites, Cas13 may cause collateral RNA cleavage, and bacterial Cas proteins may trigger pre‐existing immunity [402, 403, 404]. Thus, transient delivery, improved guide design, and safer Cas variants will be essential for future antiviral translation.

### 4.6 Cellular Therapies

Cellular therapies use transfused or engineered immune cells to eliminate virus‐infected cells, and they are especially valuable when patients cannot mount effective antiviral immunity, such as after hematopoietic stem cell transplantation or in chronic infection. Adoptive transfer of VST cells has been most successful in transplant medicine, where CMV, EBV, adenovirus, BK virus, and related infections are major causes of morbidity [405, 406]. Donor‐derived CMV‐specific T cells can control refractory CMV, while EBV‐specific T cells can eradicate posttransplant lymphoproliferative disease, and CMV‐specific infusions can also provide durable immune surveillance against relapse [407, 408, 409, 410]. Current platforms include selected or expanded donor T cells, as well as multivirus VST products that recognize several pathogens at once, including CMV, EBV, adenovirus, BK, and HHV6, without causing graft‐versus‐host disease [411, 412, 413]. The development of “off‐the‐shelf” VST banks from partially HLA‐matched donors has further improved access, with third‐party products showing response rates of about 60–70% in severe viral infections and minimal toxicity [414, 415]. This approach is now expanding beyond transplantation, including refractory BK virus nephropathy and JC virus PML.

CAR T cells (CAR‐T) and CAR‐NK engineering extend cellular therapy to chronic viral disease. In HIV, CAR‐T cells have been designed to recognize HIV envelope on infected cells; early CD4‐based CARs were safe but weak, whereas newer constructs such as M10 CAR‐T show stronger antiviral activity in preclinical models and sustained control in humanized mice [416, 417, 418]. Clinical trials are now testing gene‐edited CAR‐T cells to reduce HIV reservoirs [417, 419, 420]. For HBV, CAR‐T and TCR‐engineered T cells can target HBsAg or HBV peptide–MHC complexes and reduce viral markers, but liver toxicity remains a major concern [421, 422]. CAR‐NK cells are also under study because they may avoid graft‐versus‐host disease while retaining innate killing capacity [423, 424]. Safety remains central. Viral T‐cell therapies can cause inflammation or off‐target tissue damage, so suicide switches such as inducible Caspase‐9 and transient mRNA‐based CAR expression are being developed. Therapeutic vaccines are generally safer but often need combination with checkpoint blockade, such as PD‐1 inhibition, to overcome immune tolerance.

## 5 Challenges, Limitations, Emerging Trends, and Future Perspectives

Despite major advances in vaccines, mAbs, DAAs, host‐targeted agents, and genetic and cellular therapies, durable control of viral diseases remains limited by interconnected biological, pharmacological, translational, and implementation barriers. Rapid viral evolution can erode vaccine effectiveness, enable antibody escape, and select drug‐resistant variants, whereas latency, persistent viral genomes, tissue reservoirs, and immune exhaustion prevent complete eradication of many chronic infections. At the same time, toxicity, restricted tissue penetration, drug–drug interactions, complex manufacturing, and unequal global access frequently limit the real‐world impact of otherwise effective interventions. Addressing these challenges will require integrated strategies that combine rational target selection, improved delivery, resistance surveillance, adaptive clinical evaluation, and equitable deployment.

### 5.1 Viral Evolution, Immune Escape, and Persistent Reservoirs

Antigenic drift, recombination, reassortment, and selection under immune or therapeutic pressure remain major obstacles to durable viral control. These processes can reduce recognition by vaccine‐induced antibodies, compromise monoclonal–antibody binding, and generate resistance to DAAs. The vulnerability is particularly pronounced for agents directed against a single variable epitope or viral protein with a relatively low genetic barrier to resistance. Influenza and SARS‐CoV‐2 illustrate the need for repeated vaccine updating because of antigenic evolution, whereas HIV, HBV, and herpesviruses demonstrate the difficulty of achieving curative treatment in the presence of persistent viral reservoirs (Table 2). Future interventions should therefore prioritize conserved and functionally constrained targets, combine agents with nonoverlapping mechanisms, and account for the effects of resistance substitutions on viral fitness. bnAb combinations, multivalent or mosaic vaccines, and antiviral regimens that target several stages of the viral life cycle are likely to provide greater breadth and durability than single‐component approaches [94, 192, 196, 197, 198, 199, 340, 341, 342, 343, 344, 345].

**TABLE 2:** State of the art for major human viral diseases.

Disease	Current status/burden	Prevention/vaccines	Therapeutics	Major recent advances	Key unmet needs/limitations
HIV	∼40 million people living with HIV worldwide; no cure to date [1, 425].	Daily oral PrEP with tenofovir–emtricitabine reduces infection risk; long‐acting injectable PrEP (cabotegravir) is more effective and reduced incidence by about two‐thirds versus oral PrEP [426, 427, 428]. No licensed HIV vaccine yet. Passive prevention with bnAbs remains experimental; VRC01 protected against susceptible strains in AMP trials but not overall infection [228, 429].	Modern ART suppresses viral replication and prevents transmission. Long‐acting injectable ART (cabotegravir/rilpivirine) allows monthly or bimonthly dosing. Lenacapavir, a twice‐yearly capsid inhibitor, is approved for multidrug‐resistant HIV [430, 431].	Long‐acting PrEP, long‐acting ART, and new drug classes have transformed prevention and treatment.	No vaccine, no broadly scalable cure, persistent latent reservoirs, and need for curative strategies such as gene therapy, shock‐and‐kill, and immunotherapy.
HBV	∼296 million chronic infections and ∼820,000 deaths annually, mainly from cirrhosis and liver cancer [432].	Highly effective prophylactic HBV vaccination has been part of infant immunization for decades and is one of the most successful viral vaccines [433, 434].	Tenofovir, entecavir, and pegylated interferon suppress replication but rarely achieve functional cure; treatment is often lifelong because viral rebound is common after stopping [434, 435, 436].	RNA interference therapies and capsid assembly modulators are advancing; some siRNA‐based therapies markedly reduce viral antigens, and combination approaches have achieved HBsAg clearance in selected patient.	No reliable curative regimen, low functional cure rates, and need for safe finite therapy that induces durable HBsAg loss.
HCV	∼58 million people infected globally and ∼290,000 deaths per year, mainly from cirrhosis and liver cancer [437].	No vaccine available. Prevention relies on screening and risk reduction [438].	Direct‐acting antivirals (DAAs) cure >95% of chronic infections with 8–12 weeks of oral therapy. Common agents include sofosbuvir, ledipasvir velpatasvir, and pibrentasvir [439, 440, 441, 442],	HCV has become a curable infection for most patients, including those with advanced liver disease; elimination programs in some countries are making major progress.	Major barriers are poor access, undiagnosed infection, reinfection in high‐risk groups, and no licensed vaccine due to high genetic diversity.
HPV	Causative agent of nearly all cervical cancers and a substantial fraction of other anogenital and oropharyngeal cancers [443].	Prophylactic bivalent, quadrivalent, and nonavalent HPV vaccines targeting high‐risk types, especially 16 and 18, are highly effective [444].	No antiviral therapy clears HPV infection; management of lesions relies on screening, excision, ablation, or surgery [445].	Real‐world vaccination has sharply reduced HPV infection, precancerous lesions, and cervical cancer; countries with high coverage show ∼87–90% reduction in cervical cancer among women vaccinated in early adolescence. Therapeutic vaccine VGX‐3100 induced regression of CIN2/3 lesions in ∼49.5% of recipients in a Phase IIb trial.	Need for wider vaccine coverage, one‐dose strategies in some settings, vaccination of boys, and nonsurgical treatment of persistent infection or established lesions.
Influenza	Causes ∼1 billion infections and 290,000–650,000 respiratory deaths annually [446].	Seasonal inactivated or live‐attenuated vaccines are updated annually, but effectiveness is only moderate (∼40–60%) and can be reduced by antigenic drift. Universal vaccine candidates are being developed against conserved targets such as HA stalk and M2e [447].	Neuraminidase inhibitors remain important; baloxavir marboxil, a cap‐dependent endonuclease inhibitor, offers single‐dose oral therapy and faster viral clearance than oseltamivir, but resistance can emerge [448].	High‐dose and adjuvanted vaccines improve protection in older adults; universal vaccine platforms and broadly neutralizing antibodies are in development.	Annual reformulation, strain mismatch, resistance to antivirals, and the lack of a truly universal, durable vaccine remain key gaps.
SARS‐CoV‐2 (COVID‐19)	Pandemic has become endemic with continued variant emergence and immune escape [449, 450].	Highly efficacious mRNA vaccines were developed rapidly; variant‐adapted boosters, including Omicron‐targeted formulations, are now used to maintain protection [451, 452].	Paxlovid (nirmatrelvir/ritonavir) is first‐line outpatient therapy; molnupiravir and remdesivir are additional options, and dexamethasone/IL‐6 inhibitors are standard for severe disease [296, 370, 453, 454].	The rapid adaptability of mRNA vaccines is a major strength; pan‐coronavirus and intranasal mucosal vaccines are in development.	Ongoing variant evolution, waning immunity, loss of monoclonal antibody efficacy, and the need for broader, longer‐lasting vaccines and prophylaxis for immunocompromised individuals.
RSV	Major cause of bronchiolitis and pneumonia in infants and illness in older adults [455].	Breakthrough prevention includes nirsevimab for infants, maternal RSV vaccination for newborn protection, and RSVpref vaccines for older adults. Nirsevimab provides season‐long passive protection after one dose [456, 457, 458].	No approved routine antiviral therapy; supportive care remains standard. Ribavirin is rarely used because benefit is limited and toxicity is a concern [459].	2023 brought key approvals: nirsevimab for infants, maternal vaccine protection for newborns, and RSVpref vaccines for adults over 60 with ∼80–90% efficacy against severe RSV disease.	Need for affordable global implementation, improved therapeutics for severe disease, and stronger uptake of new preventive tools.
Ebola and other filoviruses	Ebola virus disease was historically highly fatal, but prevention and treatment have improved substantially [460].	rVSV‐ZEBOV (Ervebo) is a highly effective live‐attenuated Ebola vaccine used in ring vaccination; it showed more than 97% efficacy after 10 days in trials and was approved in 2019 [260, 461, 462, 463].	REGN‐EB3 (Inmazeb) and mAb114/ansuvimab (Ebanga) are approved antibody therapies that significantly reduce mortality when given early [234, 464].	Ebola is now both preventable and treatable in outbreak settings, marking a major shift in outbreak management.	No licensed vaccine or therapy for Marburg or Sudan ebolavirus; pan‐filovirus countermeasures are still lacking.
Dengue, Zika, chikungunya	Important mosquito‐borne diseases with complex immunology and, for dengue, major global burden [465].	Dengvaxia is effective mainly in seropositive individuals but may increase severe dengue risk in seronegatives because of ADE; TAK‐003 shows broader protection and was approved in several regions. No approved vaccine for Zika yet. Chikungunya vaccine development is advancing, with VLA1553 the leading candidate [466, 467, 468, 469].	No antiviral treatment exists for Zika or chikungunya; dengue management is supportive [470].	TAK‐003 offers a more practical dengue vaccine strategy; chikungunya VLP and live‐attenuated candidates show strong immunogenicity.	ADE, serostatus‐dependent vaccine risk, cross‐reactive flavivirus immunity, lack of Zika vaccine, and no specific antivirals for Zika/chikungunya.
CMV, HSV, and VZV	Ubiquitous herpesviruses; CMV and HSV still lack licensed vaccines, while VZV has effective vaccines [471].	For VZV, live‐attenuated varicella vaccine and recombinant zoster vaccine (Shingrix) are effective; Shingrix shows ∼90% efficacy against shingles in adults >50. No licensed CMV or HSV vaccine yet [472, 473].	CMV: letermovir for prophylaxis and maribavir for resistant/refractory infection have transformed transplant management. HSV: acyclovir, valacyclovir, and famciclovir remain standard; pritelivir is a promising new helicase‐primase inhibitor. VZV: acyclovir and related drugs remain the mainstay [474, 475, 476, 477].	Letermovir and maribavir have improved CMV prevention and treatment; Shingrix is a major subunit vaccine success.	Need for CMV vaccine, HSV vaccine, and better strategies to eliminate latent HSV or prevent congenital CMV.
Emerging zoonoses (Nipah, Hendra, Lassa, etc.)	Sporadic but often severe outbreaks; major concern for epidemic preparedness [478].	No licensed human vaccines yet for Nipah or Lassa. Candidate vaccines are advancing, including Nipah subunit, mRNA, and adenoviral vaccines; Hendra prevention in horses has reduced spillover. Lassa vaccine candidates are in Phase I–II [479, 480, 481].	No approved specific therapies for Nipah or Lassa. m102.4 monoclonal antibody has been used compassionately for Nipah/Hendra exposures; ribavirin remains the traditional therapy for Lassa, though evidence is limited [482, 483].	Research platforms and outbreak readiness have improved; mAb stockpiling and trial infrastructure are being developed for future outbreaks.	Lack of licensed countermeasures, reliance on supportive care and public health measures, and the need to move candidates from trials to approval.

*Abbreviations*: ADE, antibody‐dependent enhancement; ART, antiretroviral therapy; bnAbs, broadly neutralizing antibodies; CMV, cytomegalovirus; COVID‐19, coronavirus disease 2019; DAAs, direct‐acting antivirals; HA, hemagglutinin; HBV, hepatitis B virus; HCV, hepatitis C virus; HIV, human immunodeficiency virus; HPV, human papillomavirus; HSV, herpes simplex virus; M2e, matrix protein 2 ectodomain; mAbs, monoclonal antibodies; PrEP, pre‐exposure prophylaxis; RNAi, RNA interference; RSV, respiratory syncytial virus; SARS‐CoV‐2, severe acute respiratory syndrome coronavirus 2; siRNA, small interfering RNA; VZV, varicella‐zoster virus; WHO, World Health Organization.

Persistent viral genomes represent a distinct challenge that cannot be addressed by conventional replication inhibitors alone. Integrated HIV proviruses, HBV covalently closed circular DNA, and latent herpesvirus episomes can remain transcriptionally silent in long‐lived cells and reactivate after treatment withdrawal [484, 485]. Anatomical sanctuaries, including the central nervous system and lymphoid follicles, may further restrict drug penetration and immune surveillance. Curative strategies will consequently require combinations that suppress active replication while exposing, permanently silencing, editing, or eliminating reservoir cells. Therapeutic vaccines, bnAbs, latency‐modifying agents, engineered T cells, and gene‐editing technologies offer complementary approaches, but their ability to reduce clinically relevant reservoirs remains to be demonstrated consistently.

### 5.2 Pharmacological, Safety, and Delivery Limitations

The clinical effectiveness of antiviral agents depends on achieving sustained drug concentrations at infected tissues without unacceptable toxicity. Many treatments have narrow therapeutic windows, limited oral bioavailability, or inadequate penetration into immune‐privileged compartments. PK boosting can improve exposure but may create extensive drug–drug interactions, as exemplified by ritonavir‐containing regimens [328, 329, 330, 331]. Long‐acting formulations can improve adherence, yet prolonged subtherapeutic pharmacological tails may select resistance when scheduled doses are missed. Better tissue‐distribution models, therapeutic drug monitoring, and formulations capable of maintaining protective concentrations at mucosal and reservoir sites are therefore needed.

Host‐targeted therapies may offer broad‐spectrum activity and higher barriers to viral resistance, but inhibition of essential cellular pathways can produce dose‐limiting toxicity [354, 355, 356, 357, 358, 359, 365]. Similarly, immunomodulators require careful timing: insufficient suppression may fail to control immunopathology, whereas excessive or premature inhibition can impair viral clearance and increase susceptibility to secondary infection [367, 368, 369, 371, 372]. Immune‐checkpoint inhibitors and adoptive cellular therapies can restore antiviral effector function but may cause hepatitis, systemic inflammation, cytokine‐mediated toxicity, or off‐target tissue damage [378, 379, 380, 381, 382, 383]. Biomarker‐guided patient selection, tunable or transient therapeutic platforms, and incorporated safety mechanisms such as suicide switches will be important for improving their therapeutic index.

Delivery remains the principal obstacle for RNA‐ and gene‐editing antivirals. Oligonucleotides and CRISPR components must reach the appropriate infected cells, escape intracellular degradation, and achieve sufficient activity without inducing off‐target editing, collateral RNA cleavage, or immune responses against delivery vectors or bacterial nucleases [385, 387, 388, 389, 390, 402, 403, 404]. Lipid nanoparticles, tissue‐specific ligands, inhaled formulations, transient messenger RNA delivery, and smaller or engineered nuclease systems may expand the range of targetable organs. However, long‐term safety, manufacturability, repeat dosing, and regulatory evaluation must be resolved before these technologies can be widely applied to viral disease.

### 5.3 Translational, Regulatory, and Global Implementation Barriers

Promising antiviral activity in cell culture or animal models frequently fails to translate into clinical benefit because experimental systems do not fully reproduce human viral diversity, immunity, comorbidities, PK, or tissue‐specific infection. This limitation is especially important for host‐targeted agents and immunomodulators, whose effects may differ markedly between species or disease stages. Preclinical development should therefore incorporate physiologically relevant organoid systems, humanized animal models, diverse viral isolates, and pharmacological exposure measurements that more closely reflect intended clinical use.

Clinical evaluation during emerging outbreaks is complicated by unpredictable case numbers, rapidly changing standards of care, heterogeneous disease severity, and delays in trial activation. Adaptive platform trials and master protocols can compare several candidates within a shared infrastructure, discontinue ineffective interventions, and prioritize promising combinations more rapidly than conventional sequential trials. Preapproved protocols, harmonized clinical endpoints, validated correlates of protection, international data‐sharing mechanisms, and ready‐to‐activate trial networks should therefore form part of preparedness planning rather than being created only after an outbreak begins.

Access and affordability remain equally important limitations. mAbs, long‐acting injectables, gene therapies, and individualized cellular products may be clinically effective but remain inaccessible in many low‐resource settings because of high cost, specialized manufacturing, injection‐based delivery, cold‐chain dependence, and limited diagnostic infrastructure. Technology transfer, regional manufacturing, thermostable formulations, simplified dosing, needle‐free delivery, and transparent procurement mechanisms will be required to prevent innovation from widening existing global health inequalities. Resistance testing and genomic surveillance must also be expanded beyond well‐resourced regions so that treatment guidelines and vaccine updates reflect globally circulating variants rather than geographically restricted datasets.

### 5.4 Emerging Trends and Future Priorities

Future antiviral development is likely to shift from pathogen‐specific, reactive approaches toward adaptable platform technologies with broad or rapidly reprogrammable activity (Table 3). Structure‐guided immunogen design, mosaic and nanoparticle antigen display, self‐amplifying RNA, and conserved‐epitope targeting may support more universal vaccines against antigenically variable viruses. Mucosal vaccination is particularly promising for respiratory and enteric infections because it can induce secretory immunoglobulin A and tissue‐resident memory responses directly at viral entry sites. Thermostable, oral, inhaled, and microarray‐patch formulations may further improve acceptance and global distribution.

**TABLE 3:** Emerging therapies against viral infections.

Strategy	Core concept/mechanism	Representative examples	Key findings/current status	Main advantages	Key limitations/future needs	References
RNA‐based therapies (siRNA/ASO)	Small interfering RNAs and antisense oligonucleotides bind viral transcripts and promote degradation or block translation, thereby silencing viral or host genes needed for replication.	Fomivirsen (first US FDA‐approved oligonucleotide drug for CMV retinitis); bepirovirsen (HBV ASO); AB‐729, VIR‐2218, JNJ‐3989, RG6346, TQA3038 (HBV siRNAs); ALN‐RSV01 (RSV); TKM‐130803 (Ebola).	Bepirovirsen produced sustained HBsAg and HBV DNA loss in ∼9–10% of chronic HBV patients in Phase IIb; AB‐729 and VIR‐2218 produced durable antigen suppression; ALN‐RSV01 reduced RSV infection in a human challenge study; TKM‐130803 provided safety/dosing information but did not improve survival in advanced Ebola.	Highly sequence‐specific; can suppress viral antigens and genomes; can be adapted rapidly to new targets; potentially useful for chronic infections and acute outbreaks.	Delivery remains the major barrier; hepatocyte targeting has advanced through GalNAc conjugation and LNPs, but extrahepatic delivery is still difficult. Long‐term safety, dosing, and breadth of application remain under study.	[392, 393, 394, 395, 486, 487, 488]
Gene editing (CRISPR)	CRISPR–Cas systems can disrupt viral genomes or host dependency factors, either ex vivo in edited cells or in vivo in infected tissues.	CCR5‐edited HSPCs for HIV resistance; EBT‐101 (in vivo CRISPR for HIV provirus excision); preclinical CRISPR for HBV and herpesviruses; topical CRISPR/Cas9 targeting HPV16/18 E6/E7 in cervical lesions.	CCR5‐edited cells engrafted safely in an HIV‐positive patient but were insufficient to control viremia after ART interruption; EBT‐101 was safe in Phase I/II but did not prevent HIV rebound off ART; HBV and HPV CRISPR studies are still early or preclinical.	Offers the possibility of permanent viral inactivation or elimination; may enable functional cures rather than chronic suppression.	Delivery to latent reservoirs, editing efficiency, specificity, and off‐target risk remain major hurdles. Ex vivo editing is safer and more controllable, while in vivo editing could reach inaccessible reservoirs.	[399, 419, 421, 422]
Nanomedicine	Nanoparticles and related structures are used for targeted drug delivery, vaccine presentation, and antiviral surface engineering.	LNPs for nucleic acid delivery; antibody‐decorated liposomes; nanoparticle inhaled remdesivir derivatives; mosaic‐8 RBD nanoparticle for pan‐sarbecovirus vaccination; Novavax NVX‐CoV2373; silver/copper nanoparticle antiviral coatings; microneedle‐linked nanoparticle systems.	Mosaic RBD nanoparticles induced broadly neutralizing responses in animals and entered first‐in‐human trials; Novavax showed ∼90% efficacy in trials; antiviral coatings can inactivate viruses rapidly on contact; many targeted delivery systems remain preclinical.	Improves tissue targeting, antigen display, and delivery efficiency; can reduce toxicity; can also support self‐sterilizing surfaces and needle‐free vaccination.	Many targeted nano‐delivery approaches are still preclinical; manufacturing, standardization, and safety remain important translational issues.	[170, 489, 490, 491, 492]
Artificial intelligence and computational design	AI/ML are used to design antigens, predict viral evolution, discover antivirals, and anticipate resistance.	EVE‐Vax; computationally optimized broadly reactive antigens (COBRA); AI‐guided drug screening for influenza, Ebola, SARS‐CoV‐2; resistance prediction tools for HIV/HBV/HCV.	AI‐generated SARS‐CoV‐2 spike variants predicted future immune‐escape mutations; COBRA influenza antigens induced broad antibody responses; AI has accelerated hit discovery and resistance forecasting.	Rapidly narrows design space; helps anticipate viral evolution; accelerates vaccine and drug discovery; useful for universal vaccine and broad‐spectrum antiviral development.	No AI‐designed vaccine is yet licensed; predictions still require experimental validation; performance depends on training data quality and biological complexity.	[493, 494, 495]
Broad‐spectrum and pan‐family antiviral strategies	Targets conserved viral epitopes, conserved viral enzymes, or host factors used by multiple viruses to achieve cross‐family activity.	Universal influenza vaccine approaches (HA stem, M2e, T‐cell vaccines such as MVA‐NP+M1); HIV bnAbs (VRC01, 3BNC117, etc.); host‐targeting agents such as maraviroc, miravirsen, interferons, camostat/nafamostat; broad nucleosides (ribavirin, favipiravir, remdesivir, molnupiravir); protease inhibitors such as nirmatrelvir.	Stem‐directed influenza bnAbs and HIV bnAb cocktails have shown promising preclinical and early clinical activity; broad nucleoside analogs and host‐targeting drugs have provided urgent outbreak responses; combination approaches improve breadth and reduce resistance.	Useful for emerging pathogens and pandemic preparedness; resistance is harder to develop when conserved viral or host targets are used; can serve as stockpiled countermeasures.	Often less potent than highly specific agents; host‐targeting drugs may cause toxicity; some broad agents face resistance or limited clinical efficacy, so combination strategies are often preferable.	[496, 497, 498, 499, 500]
Mucosal and needle‐free immunization	Vaccines delivered intranasally, orally, or via microneedle patches aim to induce local mucosal IgA and tissue‐resident T cells, or improve acceptance and logistics.	Intranasal influenza vaccine (LAIV/FluMist); intranasal COVID‐19 candidates such as dNS1‐RBD, BBV154, and aerosolized adenoviral vaccines; oral polio vaccine (OPV), oral rotavirus vaccines; microneedle influenza patches and experimental polio/COVID patches.	dNS1‐RBD showed protective efficacy in Phase III against Omicron; oral vaccines are highly effective for gut pathogens and reduce transmission; influenza microneedle patches were as immunogenic as IM vaccination and preferred by participants.	Better match for mucosal pathogens; may reduce transmission more effectively than intramuscular vaccines; needle‐free formats improve acceptance, simplify administration, and may reduce waste/cold‐chain dependence.	Intranasal/oral vaccines can have variable immunogenicity and formulation challenges; achieving consistent mucosal protection and large‐scale deployment remains difficult.	[501, 502, 503, 504, 505]
Combination and platform approaches	Uses modular vaccine/drug platforms and multiagent combinations to speed response and improve efficacy.	mRNA platforms; adenoviral and VSV vector platforms; combination antivirals and immunomodulators; heterologous prime‐boost regimens; platform manufacturing “vaccine libraries.”	The COVID‐19 response validated plug‐and‐play mRNA manufacturing and rapid variant updates; combination therapy improved outcomes in some infections; platform vaccines can be adapted quickly to new pathogens.	Dramatically shortens development timelines; supports rapid outbreak response; combination strategies can improve potency and reduce escape.	Requires regulatory flexibility, manufacturing readiness, and coordinated global infrastructure; platform success depends on robust prior investment and preapproved frameworks.	[277, 506, 507, 508, 509, 510]

*Abbreviations*: AI, artificial intelligence; ART, antiretroviral therapy; ASO, antisense oligonucleotide; bnAbs, broadly neutralizing antibodies; Cas9, CRISPR‐associated protein 9; CMV, cytomegalovirus; COBRA, computationally optimized broadly reactive antigen; CRISPR, clustered regularly interspaced short palindromic repeats; E6/E7, early viral oncoproteins 6 and 7; US FDA, US Food and Drug Administration; GalNAc, N‐acetylgalactosamine; HA, hemagglutinin; HBsAg, hepatitis B surface antigen; HBV, hepatitis B virus; HCV, hepatitis C virus; HIV, human immunodeficiency virus; HPV, human papillomavirus; HSPCs, hematopoietic stem/progenitor cells; IM, intramuscular; LAIV, live attenuated influenza vaccine; LNPs, lipid nanoparticles; M2e, matrix protein 2 ectodomain; ML, machine learning; mRNA, messenger RNA; MVA, modified vaccinia Ankara; OPV, oral polio vaccine; RBD, receptor‐binding domain; RSV, respiratory syncytial virus; SARS‐CoV‐2, severe acute respiratory syndrome coronavirus 2; siRNA, small interfering RNA; VSV, vesicular stomatitis virus.

Broad‐spectrum and pan‐family antivirals are also important preparedness priorities. Targeting conserved polymerase, protease, capsid, or fusion mechanisms may yield agents that can be deployed against related viruses before pathogen‐specific drugs become available. Selective targeting of shared host‐dependency factors could further extend antiviral breadth, provided that essential cellular functions are preserved. Future regimens will probably combine viral and host‐directed agents to increase potency, reduce resistance, and modulate damaging inflammation simultaneously.

Multimodal immunotherapy represents another important direction. Therapeutic vaccines may be combined with checkpoint modulation, bnAbs, latency‐targeting agents, or adoptive VST cells to overcome immune exhaustion and improve clearance of persistently infected cells. Such combinations should be developed rationally according to viral burden, reservoir location, antigen expression, and the functional state of host immunity rather than by empirically adding immunotherapies with overlapping toxicity.

Artificial intelligence, structural modeling, high‐throughput screening, viral genomics, proteomics, and CRISPR‐based functional screens are expected to accelerate antigen selection, drug discovery, resistance prediction, and identification of host‐dependency factors. Their greatest value will arise when computational predictions are integrated with experimental validation, clinical datasets, and real‐time genomic surveillance. Surveillance systems should link the detection of antigenic or resistance‐associated variants to predefined regulatory pathways for updating vaccines, antibodies, diagnostics, and treatment recommendations.

Finally, preparedness for emerging zoonoses requires a One Health framework integrating human, animal, and environmental surveillance. Prototype vaccines, antibody libraries, broad‐spectrum compounds, manufacturing templates, and clinical protocols should be developed in advance for priority viral families. Collectively, these approaches could transform antiviral development from a largely reactive process into a coordinated system capable of anticipating viral emergence, rapidly adapting interventions, and delivering them equitably across populations.

## 6 Conclusion

The expanding range of prophylactic and therapeutic interventions has transformed the management of several viral diseases, demonstrating that viral entry, replication, immune recognition, and host dependency pathways can all be exploited clinically. Vaccines and passive immunization remain central to prevention, while DAAs have enabled durable suppression or cure in infections such as HIV and HCV. Host‐targeted agents, immunomodulators, therapeutic vaccines, RNA therapeutics, gene editing, and cellular therapies further broaden the available intervention landscape, particularly for persistent infections and emerging pathogens. Nevertheless, antigenic evolution, antiviral resistance, latent reservoirs, tissue‐specific drug delivery, immune‐mediated toxicity, and unequal access continue to limit long‐term disease control. Future progress will depend on integrating broadly protective vaccines, high‐barrier combination therapies, precision immunomodulation, adaptable delivery platforms, and genomic surveillance within coordinated global preparedness systems. Such an integrated approach will be essential not only for improving outcomes against established viral diseases but also for responding rapidly and equitably to future viral threats.

The evidence across modalities also indicates that prevention and treatment should be designed as complementary layers rather than competing alternatives. Vaccines and long‐acting antibodies can reduce infection or severe disease at the population level, whereas rapidly deployable small molecules, combination regimens, and immune‐based therapies are required for breakthrough, chronic, or immunocompromised cases. No single platform is likely to overcome the combined problems of antigenic drift, within‐host evolution, reservoirs, and heterogeneous host immunity. Product and regimen design should therefore emphasize conserved targets, high barriers to resistance, adequate exposure in tissue reservoirs, biomarkers that demonstrate target engagement, and delivery formats that support adherence across diverse clinical settings.

The most important translational priority is to connect discovery with an adaptive evidence system. Structure‐guided and artificial‐intelligence‐assisted design, functional genomics, and high‐throughput screening can accelerate candidate identification, but clinical development must incorporate representative populations, rigorous PK and PD assessment, prospective resistance monitoring, and endpoints that measure durable clinical benefit. Platform manufacturing, prototype vaccines and antibodies for priority viral families, and pan‐family antiviral libraries should be established before outbreaks. Real‐time genomic and phenotypic surveillance should then trigger predefined pathways for updating diagnostics, vaccines, antibodies, and treatment recommendations, thereby shortening the interval between viral change and an effective countermeasure.

Finally, scientific efficacy will have limited impact without equitable implementation. Affordability, heat‐stable or simplified formulations, decentralized diagnostics, scalable manufacturing, technology transfer, pharmacovigilance, and antiviral stewardship should be treated as design requirements from the outset. A One Health perspective that links human, animal, and environmental surveillance is equally important for identifying zoonotic threats before sustained transmission occurs. In conclusion, long‐term preparedness against viral threats requires broadly protective vaccines and preventive measures, antiviral treatments that maintain effective drug concentrations and limit resistance, targeted control of harmful immune responses, real‐time surveillance, and delivery systems that provide all populations with rapid and equitable access to prevention and treatment.

## Author Contributions

Sawar Khan and Ayesha Nisar: conceptualization, literature search, data curation, and Writing – original draft. Xiaoxu Li, Hao Yu, Abdul Qadeer, Sardar Azhar Mehmood, Imane Lamraoui, Sijie Liu, Lipeng Zhu, and Xu Deng: literature search, Writing – review and editing, and visualization. Bo Kong, Kejun Zhong, and Zanxian Xia: conceptualization, supervision, Writing – review and editing, and funding acquisition. All authors have read and agreed to the published version of the manuscript.

## Funding

This work was supported by Beijing Life Science Academy (BLSA) (2024100CB0170 and 2024200CD0020), the National Natural Science Foundation of China (32571465 and U21A20384), the Science and Technology Innovation Program of Hunan Province (2024WZ9001), and Open Project Program of the State Key Laboratory of Proteomics (SKLP‐O201805).

## Ethics Statement

The authors have nothing to report.

## Conflicts of Interest

The authors declare no conflicts of interest.

## Data Availability

The authors have nothing to report.

## References

[1] GBD , “Global, Regional, and National Burden of HIV/AIDS, 1990–2021, and Forecasts to 2050, for 204 Countries and Territories: The Global Burden of Disease Study 2021,” The Lancet HIV 11, no. 12 (2024):e807–e822. 10.1016/S2352-3018(24)00212-1 PMC11612058 39608393

[2] A. D. Iuliano , K. M. Roguski , H. H. Chang , et al., “Estimates of Global Seasonal Influenza‐Associated Respiratory Mortality: A Modelling Study,” The Lancet 391, no. 10127 (2018): 1285–1300. 10.1016/S0140-6736(17)33293-2 PMC5935243 29248255

[3] N. Veracruz , R. G. Gish , R. Cheung , A. S. Chitnis , and R. J. Wong , “Global Trends and the Impact of Chronic Hepatitis B and C on Disability‐Adjusted Life Years,” Liver International 42, no. 10 (2022): 2145–2153. 10.1111/liv.15347 35753064

[4] Y. Li , X. Wang , D. M. Blau , et al., “Global, Regional, and National Disease Burden Estimates of Acute Lower Respiratory Infections due to Respiratory Syncytial Virus in Children Younger Than 5 Years in 2019: A Systematic Analysis,” The Lancet 399, no. 10340 (2022): 2047–2064. 10.1016/S0140-6736(22)00478-0 PMC7613574 35598608

[5] S. Bhatt , P. W. Gething , O. J. Brady , et al., “The Global Distribution and Burden of Dengue,” Nature 496, no. 7446 (2013): 504–507. 10.1038/nature12060 PMC3651993 23563266

[6] J. R. Romero , “Palivizumab Prophylaxis of Respiratory Syncytial Virus Disease From 1998 to 2002: Results From Four Years of Palivizumab Usage,” The Pediatric Infectious Disease Journal 22, no. 2 Suppl (2003): S46–S54. 10.1097/01.inf.0000053885.34703.84 12671452

[7] S. Mulangu , L. E. Dodd , R. T. Davey , et al., “A Randomized, Controlled Trial of Ebola Virus Disease Therapeutics,” New England Journal of Medicine 381, no. 24 (2019): 2293–2303. 10.1056/NEJMoa1910993 PMC10680050 31774950

[8] E. H. Tam , Y. Peng , M. X. Y. Cheah , C. Yan , and T. Xiao , “Neutralizing Antibodies to Block Viral Entry and for Identification of Entry Inhibitors,” Antiviral Research 224 (2024): 105834. 10.1016/j.antiviral.2024.105834 38369246

[9] H. Lu , Y. Liu , Y. Song , et al., “Neutralizing Antibodies vs. Viruses: Interacting Mechanisms and Escape Tactics,” Microorganisms 13, no. 9 (2025): 2199. 10.3390/microorganisms13092199 PMC12472874 41011531

[10] Y. Liu , Q. Cao , S. Li , Z. Li , and X. Wang , “Advances in Antiviral Agents: A Concise Review,” Animals and Zoonoses 2, no. 3 (2026): 349–355, In Press.

[11] M. Lakadamyali , M. J. Rust , and X. Zhuang , “Endocytosis of Influenza Viruses,” Microbes and Infection 6, no. 10 (2004): 929–936. 10.1016/j.micinf.2004.05.002 PMC2715838 15310470

[12] N. M. Bouvier and P. Palese , “The Biology of Influenza Viruses,” Vaccine 26 Suppl 4, no. Suppl 4 (2008): D49–D53. 10.1016/j.vaccine.2008.07.039 PMC3074182 19230160

[13] C. B. Jackson , M. Farzan , B. Chen , and H. Choe , “Mechanisms of SARS‐CoV‐2 Entry Into Cells,” Nature Reviews Molecular Cell Biology 23, no. 1 (2022): 3–20. 10.1038/s41580-021-00418-x PMC8491763 34611326

[14] M. Laureti , D. Narayanan , J. Rodriguez‐Andres , J. K. Fazakerley , and L. Kedzierski , “Flavivirus Receptors: Diversity, Identity, and Cell Entry,” Frontiers in Immunology 9 (2018): 2180. 10.3389/fimmu.2018.02180 PMC6168832 30319635

[15] C. B. Wilen , J. C. Tilton , and R. W. Doms , “HIV: Cell Binding and Entry,” Cold Spring Harbor Perspectives in Medicine 2, no. 8 (2012): a006866. 10.1101/cshperspect.a006866 PMC3405824 22908191

[16] D. S. Dimitrov , “Virus Entry: Molecular Mechanisms and Biomedical Applications,” Nature Reviews Microbiology 2, no. 2 (2004): 109–122. 10.1038/nrmicro817 PMC7097642 15043007

[17] S. Duffy , “Why Are RNA Virus Mutation Rates So Damn High?,” PLoS Biology 16, no. 8 (2018): e3000003. 10.1371/journal.pbio.3000003 PMC6107253 30102691

[18] S. Payne , “Chapter 10 ‐ Introduction to RNA Viruses,” in Viruses: From Understanding to Investigation, ed. S. Payne (Academic Press, 2017).

[19] C. Ferrer‐Orta , D. Ferrero , and N. Verdaguer , “RNA‐Dependent RNA Polymerases of Picornaviruses: From the Structure to Regulatory Mechanisms,” Viruses 7, no. 8 (2015): 4438–4460. 10.3390/v7082829 PMC4576190 26258787

[20] A. Herschhorn and A. Hizi , “Retroviral Reverse Transcriptases,” Cellular and Molecular Life Sciences 67, no. 16 (2010): 2717–2747. 10.1007/s00018-010-0346-2 PMC11115783 20358252

[21] A. Chameettachal , F. Mustafa , and T. A. Rizvi , “Understanding Retroviral Life Cycle and Its Genomic RNA Packaging,” Journal of Molecular Biology 435, no. 3 (2023): 167924. 10.1016/j.jmb.2022.167924 36535429

[22] U. Hofer , “Fooling the Coronavirus Proofreading Machinery,” Nature Reviews Microbiology 11, no. 10 (2013): 662–663. 10.1038/nrmicro3125 PMC7097040 24018385

[23] A. S. Lauring , J. Frydman , and R. Andino , “The Role of Mutational Robustness in RNA Virus Evolution,” Nature Reviews Microbiology 11, no. 5 (2013): 327–336. 10.1038/nrmicro3003 PMC3981611 23524517

[24] E. C. Smith , H. Blanc , M. Vignuzzi , and M. R. Denison , “Coronaviruses Lacking Exoribonuclease Activity Are Susceptible to Lethal Mutagenesis: Evidence for Proofreading and Potential Therapeutics,” PLoS Pathogens 9, no. 8 (2013): e1003565. 10.1371/journal.ppat.1003565 PMC3744431 23966862

[25] A. J. Cann , “Chapter 4 ‐ Replication,” in Principles of Molecular Virology, 5th ed, ed. A. J. Cann (Academic Press, 2012).

[26] V. Mas and J. A. Melero , “Entry of Enveloped Viruses Into Host Cells: Membrane Fusion,” Sub‐Cellular Biochemistry 105 (2024): 567–592. 10.1007/978-3-031-65187-8_16 39738958

[27] W. Zheng and Y. J. Tao , “Structure and Assembly of the Influenza a Virus Ribonucleoprotein Complex,” FEBS Letters 587, no. 8 (2013): 1206–1214. 10.1016/j.febslet.2013.02.048 23499938

[28] W. K. Dawson , M. Lazniewski , and D. Plewczynski , “RNA Structure Interactions and Ribonucleoprotein Processes of the Influenza a Virus,” Briefings in Functional Genomics 17, no. 6 (2018): 402–414. 10.1093/bfgp/elx028 PMC6252904 29040388

[29] A. J. Te Velthuis and E. Fodor , “Influenza Virus RNA Polymerase: Insights Into the Mechanisms of Viral RNA Synthesis,” Nature Reviews Microbiology 14, no. 8 (2016): 479–493. 10.1038/nrmicro.2016.87 PMC4966622 27396566

[30] M. Wachsmuth‐Melm , S. Peterl , A. O'Riain , et al., “Visualizing Influenza a Virus Assembly by in Situ Cryo‐Electron Tomography,” Nature Communications 16, no. 1 (2025): 9394. 10.1038/s41467-025-65117-z PMC12550032 41130956

[31] S. Sotcheff and A. Routh , “Understanding Flavivirus Capsid Protein Functions: The Tip of the Iceberg,” Pathogens 9, no. 1 (2020): 42. 10.3390/pathogens9010042 PMC7168633 31948047

[32] L. A. Byk and A. V. Gamarnik , “Properties and Functions of the Dengue Virus Capsid Protein,” Annual Review of Virology 3, no. 1 (2016): 263–281. 10.1146/annurev-virology-110615-042334 PMC5417333 27501261

[33] G. Gerold , J. Bruening , B. Weigel , and T. Pietschmann , “Protein Interactions During the Flavivirus and Hepacivirus Life Cycle,” Molecular & Cellular Proteomics 16, no. 4 (2017): S75–S91. 10.1074/mcp.R116.065649 PMC5393392 28077444

[34] Z. A. Bornholdt , T. Noda , D. M. Abelson , et al., “Structural Rearrangement of Ebola Virus Vp40 Begets Multiple Functions in the Virus Life Cycle,” Cell 154, no. 4 (2013): 763–774. 10.1016/j.cell.2013.07.015 PMC4138722 23953110

[35] S. P. Soni and R. V. Stahelin , “The Ebola Virus Matrix Protein Vp40 Selectively Induces Vesiculation From Phosphatidylserine‐Enriched Membranes,” Journal of Biological Chemistry 289, no. 48 (2014): 33590–33597. 10.1074/jbc.M114.586396 PMC4246110 25315776

[36] A.‐D. Werner , M. Schauflinger , M. J. Norris , et al., “The C‐Terminus of Sudan Ebolavirus Vp40 Contains a Functionally Important Cxnc Motif, a Target for Redox Modifications,” Structure 31, no. 9 (2023): 1038–1051.e7. 10.1016/j.str.2023.06.004 37392738

[37] A. J. Battisti , G. Meng , D. C. Winkler , et al., “Structure and Assembly of a Paramyxovirus Matrix Protein,” Proceedings of the National Academy of Sciences of the United States of America 109, no. 35 (2012): 13996–14000. 10.1073/pnas.1210275109 PMC3435216 22891297

[38] N. M. Bell and A. M. L. Lever , “HIV Gag Polyprotein: Processing and Early Viral Particle Assembly,” Trends in Microbiology 21, no. 3 (2013): 136–144. 10.1016/j.tim.2012.11.006 23266279

[39] S. Mattei , A. Tan , B. Glass , B. Müller , H. G. Kräusslich , and J. A. G. Briggs , “High‐Resolution Structures of HIV‐1 Gag Cleavage Mutants Determine Structural Switch for Virus Maturation,” Proceedings of the National Academy of Sciences of the United States of America 115, no. 40 (2018): E9401–E9410. 10.1073/pnas.1811237115 PMC6176557 30217893

[40] B. He , B. Wilson , S.‐H. Chen , et al., “Molecular Engineering of Virus Tropism,” International Journal of Molecular Sciences 25, no. 20 (2024): 11094. 10.3390/ijms252011094 PMC11508178 39456875

[41] S. W. Bird and K. Kirkegaard , “Escape of Non‐Enveloped Virus From Intact Cells,” Virology 479–480 (2015): 444–449. 10.1016/j.virol.2015.03.044 PMC4440412 25890822

[42] I. A. Owusu , O. Quaye , K. D. Passalacqua , and C. E. Wobus , “Egress of Non‐Enveloped Enteric RNA Viruses,” Journal of General Virology 102, no. 3 (2021): 001557. 10.1099/jgv.0.001557 PMC8515858 33560198

[43] J. Klingler , H. Anton , E. Réal , et al., “How HIV‐1 Gag Manipulates Its Host Cell Proteins: A Focus on Interactors of the Nucleocapsid Domain,” Viruses 12, no. 8 (2020): 888. 10.3390/v12080888 PMC7471995 32823718

[44] E. O. Freed , “HIV‐1 Assembly, Release and Maturation,” Nature Reviews Microbiology 13, no. 8 (2015): 484–496. 10.1038/nrmicro3490 PMC6936268 26119571

[45] A. York , “A Deadly Escape,” Nature Reviews Microbiology 21, no. 6 (2023): 345. 10.1038/s41579-023-00897-z 37059878

[46] N. Cifuentes‐Munoz , F. El Najjar , and R. E. Dutch , “Viral Cell‐to‐Cell Spread: Conventional and Non‐Conventional Ways,” Advances in Virus Research 108 (2020): 85–125. 10.1016/bs.aivir.2020.09.002 PMC7522014 33837723

[47] X. Yu , Y. Zhu , G. Yin , Y. Wang , X. Shi , and G. Cheng , “Exploiting Hosts and Vectors: Viral Strategies for Facilitating Transmission,” EMBO Reports 25, no. 8 (2024): 3187–3201. 10.1038/s44319-024-00214-6 PMC11315993 39048750

[48] J. Buchrieser , J. Dufloo , M. Hubert , et al., “Syncytia Formation by SARS‐CoV‐2‐Infected Cells,” The EMBO Journal 39, no. 23 (2020): e106267. 10.15252/embj.2020106267 PMC7646020 33051876

[49] K. V. Tieu , M. Espey , A. Narayanan , R. L. Heise , F. Alem , and D. E. Conway , “SARS‐CoV‐2 S‐Protein Expression Drives Syncytia Formation in Endothelial Cells,” Scientific Reports 15, no. 1 (2025): 3549. 10.1038/s41598-025-86242-1 PMC11775288 39875448

[50] C. Jolly , I. Mitar , and Q. J. Sattentau , “Adhesion Molecule Interactions Facilitate Human Immunodeficiency Virus Type 1‐Induced Virological Synapse Formation Between T Cells,” Journal of Virology 81, no. 24 (2007): 13916–13921. 10.1128/JVI.01585-07 PMC2168851 17913807

[51] K. M. Law , N. Satija , A. M. Esposito , and B. K. Chen , “Cell‐to‐Cell Spread of HIV and Viral Pathogenesis,” Advances in Virus Research 95 (2016): 43–85. 10.1016/bs.aivir.2016.03.001 PMC12989982 27112280

[52] L. M. Agosto , P. D. Uchil , and W. Mothes , “HIV Cell‐to‐Cell Transmission: Effects on Pathogenesis and Antiretroviral Therapy,” Trends in microbiology 23, no. 5 (2015): 289–295. 10.1016/j.tim.2015.02.003 PMC4417442 25766144

[53] R. Bello‐Morales , S. Andreu , and J. A. López‐Guerrero , “The Role of Herpes Simplex Virus Type 1 Infection in Demyelination of the Central Nervous System,” International Journal of Molecular Sciences 21, no. 14 (2020): 5026. 10.3390/ijms21145026 PMC7404202 32708697

[54] W. Lv , Z. Li , S. Wang , J. He , and L. Zhang , “A Role for Tunneling Nanotubes in Virus Spread,” Frontiers in Microbiology 15 (2024): 1356415. 10.3389/fmicb.2024.1356415 PMC10904554 38435698

[55] J. D. Londino , A. Lazrak , J. F. Collawn , Z. Bebok , K. S. Harrod , and S. Matalon , “Influenza Virus Infection Alters Ion Channel Function of Airway and Alveolar Cells: Mechanisms and Physiological Sequelae,” American Journal of Physiology‐Lung Cellular and Molecular Physiology 313, no. 5 (2017): L845–L858. 10.1152/ajplung.00244.2017 PMC5792181 28775098

[56] S. M. Karst and S. A. Tibbetts , “Recent Advances in Understanding Norovirus Pathogenesis,” Journal of Medical Virology 88, no. 11 (2016): 1837–1843. 10.1002/jmv.24559 PMC5203933 27110852

[57] K. Y. Green , S. S. Kaufman , B. M. Nagata , et al., “Human Norovirus Targets Enteroendocrine Epithelial Cells in the Small Intestine,” Nature Communications 11, no. 1 (2020): 2759. 10.1038/s41467-020-16491-3 PMC7265440 32488028

[58] T. Shimizu , “RNA Recognition in Toll‐Like Receptor Signaling,” Current Opinion in Structural Biology 88 (2024): 102913. 10.1016/j.sbi.2024.102913 39168045

[59] J. Rehwinkel and M. U. Gack , “Rig‐I‐Like Receptors: Their Regulation and Roles in RNA Sensing,” Nature Reviews Immunology 20, no. 9 (2020): 537–551. 10.1038/s41577-020-0288-3 PMC7094958 32203325

[60] X. Luan , L. Wang , G. Song , and W. Zhou , “Innate Immune Responses to RNA: Sensing and Signaling,” Frontiers in Immunology 15 (2024): 1287940. 10.3389/fimmu.2024.1287940 PMC10854198 38343534

[61] M. van Huizen and M. U. Gack , “The Rig‐I‐Like Receptor Family of Immune Proteins,” Molecular Cell 85, no. 20 (2025): 3793–3806. 10.1016/j.molcel.2025.09.008 PMC12616489 41106369

[62] Q. Chen , L. Sun , and Z. J. Chen , “Regulation and Function of the cGAS–STING Pathway of Cytosolic DNA Sensing,” Nature Immunology 17, no. 10 (2016): 1142–1149. 10.1038/ni.3558 27648547

[63] K. Zhang , Q. Huang , X. Li , et al., “The Cgas‐Sting Pathway in Viral Infections: A Promising Link Between Inflammation, Oxidative Stress and Autophagy,” Frontiers in Immunology 15 (2024): 1352479. 10.3389/fimmu.2024.1352479 PMC10902852 38426093

[64] J. W. Schoggins and C. M. Rice , “Interferon‐Stimulated Genes and Their Antiviral Effector Functions,” Current Opinion in Virology 1, no. 6 (2011): 519–525. 10.1016/j.coviro.2011.10.008 PMC3274382 22328912

[65] M. Zannikou , E. N. Fish , and L. C. Platanias , “Signaling by Type I Interferons in Immune Cells: Disease Consequences,” Cancers 16, no. 8 (2024): 1600. 10.3390/cancers16081600 PMC11049350 38672681

[66] D. Corti and A. Lanzavecchia , “Broadly Neutralizing Antiviral Antibodies,” Annual Review of Immunology 31 (2013): 705–742. 10.1146/annurev-immunol-032712-095916 23330954

[67] T. Swigut , L. Alexander , J. Morgan , et al., “Impact of Nef‐Mediated Downregulation of Major Histocompatibility Complex Class I on Immune Response to Simian Immunodeficiency Virus,” Journal of Virology 78, no. 23 (2004): 13335–13344. 10.1128/JVI.78.23.13335-13344.2004 PMC525019 15542684

[68] E. J. Wherry and R. Ahmed , “Memory Cd8 T‐Cell Differentiation During Viral Infection,” Journal of Virology 78, no. 11 (2004): 5535–5545. 10.1128/JVI.78.11.5535-5545.2004 PMC415833 15140950

[69] M. A. Cox and A. J. Zajac , “Shaping Successful and Unsuccessful CD8 T Cell Responses Following Infection,” Journal of Biomedicine and Biotechnology 2010 (2010): 1–12. 10.1155/2010/159152 PMC2850140 20379363

[70] J. Kervevan and L. A. Chakrabarti , “Role of Cd4+ T Cells in the Control of Viral Infections: Recent Advances and Open Questions,” International Journal of Molecular Sciences 22, no. 2 (2021): 523. 10.3390/ijms22020523 PMC7825705 33430234

[71] J. Treanor , “Influenza Vaccine — Outmaneuvering Antigenic Shift and Drift,” New England Journal of Medicine 350, no. 3 (2004): 218–220. 10.1056/NEJMp038238 14724300

[72] K. Wagh , B. H. Hahn , and B. Korber , “Hitting the Sweet Spot: Exploiting HIV‐1 Glycan Shield for Induction of Broadly Neutralizing Antibodies,” Current Opinion in HIV and AIDS 15, no. 5 (2020): 267–274. 10.1097/COH.0000000000000639 PMC7877895 32675574

[73] Y. Watanabe , T. A. Bowden , I. A. Wilson , and M. Crispin , “Exploitation of Glycosylation in Enveloped Virus Pathobiology,” Biochimica et Biophysica Acta (BBA)—General Subjects 1863, no. 10 (2019): 1480–1497. 10.1016/j.bbagen.2019.05.012 PMC6686077 31121217

[74] O. C. Grant , D. Montgomery , K. Ito , and R. J. Woods , “Analysis of the SARS‐CoV‐2 Spike Protein Glycan Shield Reveals Implications for Immune Recognition,” Scientific reports 10, no. 1 (2020): 14991. 10.1038/s41598-020-71748-7 PMC7490396 32929138

[75] R. M. van der Sluis , R. E. Jeeninga , and B. Berkhout , “Establishment and Molecular Mechanisms of HIV‐1 Latency in T Cells,” Current Opinion in Virology 3, no. 6 (2013): 700–706. 10.1016/j.coviro.2013.07.006 23953324

[76] M. P. Nicoll , J. T. Proença , and S. Efstathiou , “The Molecular Basis of Herpes Simplex Virus Latency,” FEMS Microbiology Reviews 36, no. 3 (2012): 684–705. 10.1111/j.1574-6976.2011.00320.x PMC3492847 22150699

[77] M. P. Nicoll , W. Hann , M. Shivkumar , et al., “The Hsv‐1 Latency‐Associated Transcript Functions to Repress Latent Phase Lytic Gene Expression and Suppress Virus Reactivation From Latently Infected Neurons,” PLOS Pathogens 12, no. 4 (2016): e1005539. 10.1371/journal.ppat.1005539 PMC4824392 27055281

[78] B. Roizman and R. J. Whitley , “An Inquiry Into the Molecular Basis of HSV Latency and Reactivation,” Annual Review of Microbiology 67 (2013): 355–374. 10.1146/annurev-micro-092412-155654 24024635

[79] K. Busman‐Sahay , C. E. Starke , M. D. Nekorchuk , and J. D. Estes , “Eliminating HIV Reservoirs for a Cure: The Issue Is in the Tissue,” Current Opinion in HIV and AIDS 16, no. 4 (2021): 200–208. 10.1097/COH.0000000000000688 PMC8171814 34039843

[80] H. H. Hirsch , P. Kardas , D. Kranz , and C. Leboeuf , “The Human JC Polyomavirus (JCPyV): Virological Background and Clinical Implications,” Apmis 121, no. 8 (2013): 685–727. 10.1111/apm.12128 23781977

[81] Y. Shen , L. Zhang , X. Xue , and S. Feng , “Molecular Mechanisms and Targets Underlying Immune Evasion by Human Cytomegalovirus,” Journal of Microbiology, Immunology and Infection 59, no. 2 (2026): 141–151. 10.1016/j.jmii.2025.06.005 40653424

[82] A. Halenius , S. Hauka , L. Dölken , et al., “Human Cytomegalovirus Disrupts the Major Histocompatibility Complex Class I Peptide‐Loading Complex and Inhibits Tapasin Gene Transcription,” Journal of Virology 85, no. 7 (2011): 3473–3485. 10.1128/JVI.01923-10 PMC3067861 21248040

[83] K. L. Collins , B. K. Chen , S. A. Kalams , B. D. Walker , and D. Baltimore , “HIV‐1 Nef Protein Protects Infected Primary Cells Against Killing by Cytotoxic T Lymphocytes,” Nature 391, no. 6665 (1998): 397–401. 10.1038/34929 9450757

[84] S. Jochum , A. Moosmann , S. Lang , W. Hammerschmidt , and R. Zeidler , “The Ebv Immunoevasins VIL‐10 and BNLF2A Protect Newly Infected B Cells From Immune Recognition and Elimination,” PLoS Pathogens 8, no. 5 (2012): e1002704. 10.1371/journal.ppat.1002704 PMC3355093 22615564

[85] J. M. Silva , C. E. C. Alves , and G. S. Pontes , “Epstein‐Barr Virus: The Mastermind of Immune Chaos,” Frontiers in Immunology 15 (2024): 1297994. 10.3389/fimmu.2024.1297994 PMC10879370 38384471

[86] W. J. Moss and D. E. Griffin , “What's Going on With Measles?,” Journal of Virology 98, no. 8 (2024): e0075824. 10.1128/jvi.00758-24 PMC11334507 39041786

[87] R. M. Cox , J. D. Wolf , N. A. Lieberman , et al., “Therapeutic Mitigation of Measles‐Like Immune Amnesia and Exacerbated Disease After Prior Respiratory Virus Infections in Ferrets,” Nature Communications 15, no. 1 (2024): 1189. 10.1038/s41467-024-45418-5 PMC10853234 38331906

[88] B. Hernaez and A. Alcamí , “Virus‐Encoded Cytokine and Chemokine Decoy Receptors,” Current Opinion in Immunology 66 (2020): 50–56. 10.1016/j.coi.2020.04.008 32408109

[89] F. J. Alvarez‐de Miranda , I. Alonso‐Sánchez , A. Alcamí , and B. Hernaez , “TNF Decoy Receptors Encoded by Poxviruses,” Pathogens 10, no. 8 (2021): 1065. 10.3390/pathogens10081065 PMC8401223 34451529

[90] Z. Al Rumaih , M. J. Tuazon Kels , E. Ng , et al., “Poxvirus‐Encoded Tnf Receptor Homolog Dampens Inflammation and Protects From Uncontrolled Lung Pathology During Respiratory Infection,” Proceedings of the National Academy of Sciences of the United States of America 117, no. 43 (2020): 26885–26894. 10.1073/pnas.2004688117 PMC7604498 33046647

[91] S. M. Pontejo , P. M. Murphy , and J. E. Pease , “Chemokine Subversion by Human Herpesviruses,” Journal of Innate Immunity 10, no. 5–6 (2018): 465–478. 10.1159/000492161 PMC6289880 30165356

[92] J. Zhao , S. He , A. Minassian , J. Li , and P. Feng , “Recent Advances on Viral Manipulation of NF‐ΚB Signaling Pathway,” Current Opinion in Virology 15 (2015): 103–111. 10.1016/j.coviro.2015.08.013 PMC4688235 26385424

[93] A. L. McNees , C. T. Garnett , and L. R. Gooding , “The Adenovirus E3 Rid Complex Protects Some Cultured Human T and B Lymphocytes From Fas‐Induced Apoptosis,” Journal of Virology 76, no. 19 (2002): 9716–9723. 10.1128/JVI.76.19.9716-9723.2002 PMC136506 12208950

[94] D. Z. H. Aw , D. X. Zhang , and M. Vignuzzi , “Strategies and Efforts in Circumventing the Emergence of Antiviral Resistance Against Conventional Antivirals,” NPJ Antimicrobials and Resistance 3, no. 1 (2025): 54. 10.1038/s44259-025-00125-z PMC12149321 40490516

[95] J. Han , J. Perez , A. Schafer , et al., “Influenza Virus: Small Molecule Therapeutics and Mechanisms of Antiviral Resistance,” Current Medicinal Chemistry 25, no. 38 (2018): 5115–5127. 10.2174/0929867324666170920165926 PMC8735713 28933281

[96] L. Strasfeld and S. Chou , “Antiviral Drug Resistance: Mechanisms and Clinical Implications,” Infectious Disease Clinics of North America 24, no. 2 (2010): 413–437. 10.1016/j.idc.2010.01.001 PMC2871161 20466277

[97] S. N. Zvornicanin , A. M. Shaqra , J. Flynn , et al., “Molecular Mechanisms of Drug Resistance and Compensation in SARS‐CoV‐2 Main Protease: The Interplay Between E166 and L50,” MBio 16, no. 5 (2025): e0406824. 10.1128/mbio.04068-24 PMC12077200 40183562

[98] S. Iketani , H. Mohri , B. Culbertson , et al., “Multiple Pathways for SARS‐CoV‐2 Resistance to Nirmatrelvir,” Nature 613, no. 7944 (2023): 558–564. 10.1038/s41586-022-05514-2 PMC9849135 36351451

[99] G. Neilsen , S. Lan , R. L. Slack , et al., “Strategy to Overcome a Nirmatrelvir Resistance Mechanism in the SARS‐CoV‐2 NSP5 Protease,” Science Advances 11, no. 23 (2025): eadv8875. 10.1126/sciadv.adv8875 PMC12143351 40479048

[100] A. Weibull Wärnberg , R. Ceña‐Diez , A. Sönnerborg , and M. A. A. van de Klundert , “Natural Occurrence of Drug Resistance Mutations to the HIV‐1 Capsid Inhibitor Lenacapavir,” HIV Medicine 26, no. 11 (2025): 1881–1886. 10.1111/hiv.70092 PMC12579888 41070397

[101] N. Margot , L. Vanderveen , V. Naik , et al., “Phenotypic Resistance to Lenacapavir and Monotherapy Efficacy in a Proof‐of‐Concept Clinical Study,” Journal of Antimicrobial Chemotherapy 77, no. 4 (2022): 989–995. 10.1093/jac/dkab503 35028668

[102] D. Omoding , N. Musinguzi , Y. Boum , et al., “Subtypes A1 and D, and Recombinant HIV‐1 Natural Polymorphisms Associated With Lenacapavir Drug Resistance in Uganda,” Journal of Antimicrobial Chemotherapy 80, no. 4 (2025): 955–961. 10.1093/jac/dkaf018 PMC11962371 39881519

[103] D. Hansen , M. R. Hendricks , S. Chang , et al., “Impact of HIV‐1 Capsid Polymorphisms on Viral Infectivity and Susceptibility to Lenacapavir,” MBio 16, no. 5 (2025): e0018725. 10.1128/mbio.00187-25 PMC12077089 40243329

[104] N. Pennetzdorfer , V. Naik , S. Demirdjian , et al., “Lenacapavir Treatment–Emergent HIV‐1 Capsid Resistance Mutations Are Frequently Associated With Replication Defects,” Science Translational Medicine 18, no. 831 (2026): eaea0947. 10.1126/scitranslmed.aea0947 41499523

[105] L. Briganti , A. S. Annamalai , S. M. Bester , et al., “Structural and Mechanistic Bases for Resistance of the M66i Capsid Variant to Lenacapavir,” MBio 16, no. 5 (2025): e0361324. 10.1128/mbio.03613-24 PMC12077090 40231850

[106] G. van Zyl , M. Prochazka , H. A. Schmidt , et al., “Lenacapavir‐Associated Drug Resistance: Implications for Scaling up Long‐Acting HIV Pre‐Exposure Prophylaxis,” Lancet HIV 12, no. 10 (2025): e732–e736. 10.1016/S2352-3018(25)00128-6 PMC12435527 40543515

[107] C. R. Boyle , T. Doan , E. Rios‐Guzman , et al., “Intra‐Host Evolution of SARS‐CoV‐2 During Persistent Infection of Pediatric COVID‐19 Patients,” Viruses 17, no. 10 (2025): 1313. 10.3390/v17101313 PMC12567731 41157585

[108] R. Lorenzo‐Redondo , A. M. de Sant'Anna Carvalho , J. F. Hultquist , and E. A. Ozer , “SARS‐CoV‐2 Genomics and Impact on Clinical Care for COVID‐19,” Journal of Antimicrobial Chemotherapy 78, no. Supplement_2 (2023): ii25–ii36. 10.1093/jac/dkad309 PMC10667012 37995357

[109] R. R. Patel , K. W. Hoover , A. Lale , J. Cabrales , K. M. Byrd , and A. P. Kourtis , “Clinical Recommendation for the Use of Injectable Lenacapavir as HIV Preexposure Prophylaxis — United States, 2025,” MMWR Morbidity and Mortality Weekly Report 74, no. 35 (2025): 541–549. 10.15585/mmwr.mm7435a1 PMC12445877 40966169

[110] C. M. Mink , “Chapter 7 ‐ Poliomyelitis (Polio) and Polioviruses,” in Netter's Infectious Diseases, ed. E. C. Jong and D. L. Stevens (W.B. Saunders, 2012).

[111] J. O. Amimo , S. A. Raev , J. Chepngeno , et al., “Rotavirus Interactions With Host Intestinal Epithelial Cells,” Frontiers in Immunology 12 (2021): 793841. 10.3389/fimmu.2021.793841 PMC8727603 35003114

[112] C. C. Chio , J. C. Chien , H. W. Chan , and H. I. Huang , “Overview of the Trending Enteric Viruses and Their Pathogenesis in Intestinal Epithelial Cell Infection,” Biomedicines 12, no. 12 (2024): 2773. 10.3390/biomedicines12122773 PMC11672972 39767680

[113] A. Coelho Ferraz , M. Bueno da Silva Menegatto , R. Lameira Souza Lima , et al., “Yellow Fever Virus Infection in Human Hepatocyte Cells Triggers an Imbalance in Redox Homeostasis With Increased Reactive Oxygen Species Production, Oxidative Stress, and Decreased Antioxidant Enzymes,” Free Radical Biology and Medicine 213 (2024): 266–273. 10.1016/j.freeradbiomed.2024.01.042 PMC10911966 38278309

[114] X. Lahaye , A. Vidy , C. Pomier , et al., “Functional Characterization of Negri Bodies (NBs) in Rabies Virus‐Infected Cells: Evidence That NBs Are Sites of Viral Transcription and Replication,” Journal of Virology 83, no. 16 (2009): 7948–7958. 10.1128/JVI.00554-09 PMC2715764 19494013

[115] J. M. Bigalke and E. E. Heldwein , “Nuclear Exodus: Herpesviruses Lead the Way,” Annual Review of Virology 3, no. 1 (2016): 387–409. 10.1146/annurev-virology-110615-042215 PMC5065387 27482898

[116] M. Maric , A. C. Haugo , W. Dauer , D. Johnson , and R. J. Roller , “Nuclear Envelope Breakdown Induced by Herpes Simplex Virus Type 1 Involves the Activity of Viral Fusion Proteins,” Virology 460–461 (2014): 128–137. 10.1016/j.virol.2014.05.010 PMC4101023 25010278

[117] Q. Kieser , R. S. Noyce , M. Shenouda , Y. J. Lin , and D. H. Evans , “Cytoplasmic Factories, Virus Assembly, and DNA Replication Kinetics Collectively Constrain the Formation of Poxvirus Recombinants,” PLoS ONE 15, no. 1 (2020): e0228028. 10.1371/journal.pone.0228028 PMC6964908 31945138

[118] N. Meade , S. DiGiuseppe , and D. Walsh , “Translational Control During Poxvirus Infection,” WIREs RNA 10, no. 2 (2019): e1515. 10.1002/wrna.1515 PMC6375802 30381906

[119] A. A. Asseri , “Respiratory Syncytial Virus: A Narrative Review of Updates and Recent Advances in Epidemiology, Pathogenesis, Diagnosis, Management and Prevention,” Journal of clinical medicine 14, no. 11 (2025): 3880. 10.3390/jcm14113880 PMC12156131 40507642

[120] L. Galati , M. V. Chiantore , M. Marinaro , and P. Di Bonito , “Human Oncogenic Viruses: Characteristics and Prevention Strategies—Lessons Learned From Human Papillomaviruses,” Viruses 16, no. 3 (2024): 416. 10.3390/v16030416 PMC10974567 38543781

[121] Y. Zhang , K. Qiu , J. Ren , Y. Zhao , and P. Cheng , “Roles of Human Papillomavirus in Cancers: Oncogenic Mechanisms and Clinical Use,” Signal Transduction and Targeted Therapy 10, no. 1 (2025): 44. 10.1038/s41392-024-02083-w PMC11760352 39856040

[122] D. I. Obanya , L. M. Wootton , and E. L. Morgan , “Advances in Understanding the Mechanisms of the Human Papillomavirus Oncoproteins,” Biochemical Society Transactions 53, no. 3 (2025): 565–577. 10.1042/BST20253041 PMC12224896 40380881

[123] D. E. Griffin , “Recovery From Viral Encephalomyelitis: Immune‐Mediated Noncytolytic Virus Clearance From Neurons,” Immunologic Research 47, no. 1 (2010): 123–133. 10.1007/s12026-009-8143-4 PMC2891389 20087684

[124] S. K. Sharma , N. Saini , and Y. Chwla , “Hepatitis B Virus: Inactive Carriers,” Virology Journal 2 (2005): 82. 10.1186/1743-422X-2-82 PMC1253537 16191199

[125] A. Khanam , J. V. Chua , and S. Kottilil , “Immunopathology of Chronic Hepatitis B Infection: Role of Innate and Adaptive Immune Response in Disease Progression,” International Journal of Molecular Sciences 22, no. 11 (2021): 5497. 10.3390/ijms22115497 PMC8197097 34071064

[126] P. Mehta , D. F. McAuley , M. Brown , E. Sanchez , R. S. Tattersall , and J. J. Manson , “COVID‐19: Consider Cytokine Storm Syndromes and Immunosuppression,” The Lancet 395, no. 10229 (2020): 1033–1034. 10.1016/S0140-6736(20)30628-0 PMC7270045 32192578

[127] M. Soy , G. Keser , and P. Atagündüz , “Pathogenesis and Treatment of Cytokine Storm in COVID‐19,” Turkish Journal of Biology 45, no. 4 (2021): 372–389. 10.3906/biy-2105-37 PMC8573840 34803441

[128] S. B. Halstead , “Dengue Antibody‐Dependent Enhancement: Knowns and Unknowns,” Microbiology Spectrum 2, no. 6 (2014), AID–0022–2014. 10.1128/microbiolspec.AID-0022-2014 26104444

[129] D. G. Aynekulu Mersha , I. van der Sterren , L. P. M. van Leeuwen , et al., “The Role of Antibody‐Dependent Enhancement in Dengue Vaccination,” Tropical Diseases, Travel Medicine and Vaccines 10, no. 1 (2024): 22. 10.1186/s40794-024-00231-2 PMC11529159 39482727

[130] J. Lin , “Virus‐Related Liver Cirrhosis: Molecular Basis and Therapeutic Options,” World Journal of Gastroenterology 20, no. 21 (2014): 6457. 10.3748/wjg.v20.i21.6457 PMC4047331 24914367

[131] B. Rehermann , “Pathogenesis of Chronic Viral Hepatitis: Differential Roles of T Cells and NK Cells,” Nature Medicine 19, no. 7 (2013): 859–868. 10.1038/nm.3251 PMC4482132 23836236

[132] J. Yao , J. Guo , and Y. Xie , “Hepatitis B Virus Induced Cirrhosis and Hepatocarcinoma: Pathogenesis and Therapeutics,” Exploration of Digestive Diseases 4 (2025): 100565.

[133] C. Gan , Y. Yuan , H. Shen , et al., “Liver Diseases: Epidemiology, Causes, Trends and Predictions,” Signal Transduction and Targeted Therapy 10, no. 1 (2025): 33. 10.1038/s41392-024-02072-z PMC11794951 39904973

[134] B. K. Gajjela and M. M. Zhou , “Calming the Cytokine Storm of COVID‐19 Through Inhibition of JAK2/STAT3 Signaling,” Drug Discovery Today 27, no. 2 (2022): 390–400. 10.1016/j.drudis.2021.10.016 PMC8553370 34743903

[135] L. G. Wang and L. Wang , “Current Strategies in Treating Cytokine Release Syndrome Triggered by Coronavirus SARS‐CoV‐2,” ImmunoTargets and Therapy 11 (2022): 23–35. 10.2147/ITT.S360151 PMC9124488 35611161

[136] A. Mahadevan , M. S. Suja , R. S. Mani , and S. K. Shankar , “Perspectives in Diagnosis and Treatment of Rabies Viral Encephalitis: Insights From Pathogenesis,” Neurotherapeutics 13, no. 3 (2016): 477–492. 10.1007/s13311-016-0452-4 PMC4965414 27324391

[137] A. N. van den Pol , “Polio, Still Lurking in the Shadows,” The Journal of Neuroscience: The Official Journal of the Society for Neuroscience 33, no. 3 (2013): 855–862. 10.1523/JNEUROSCI.2861-12.2013 PMC3633445 23325224

[138] S. Ohka , C. Nihei , M. Yamazaki , and A. Nomoto , “Poliovirus Trafficking Toward Central Nervous System via Human Poliovirus Receptor‐Dependent and ‐Independent Pathway,” Frontiers in Microbiology 3 (2012): 147. 10.3389/fmicb.2012.00147 PMC3328850 22529845

[139] B. Mlynarczyk‐Bonikowska and L. Rudnicka , “HPV Infections—Classification, Pathogenesis, and Potential New Therapies,” International Journal of Molecular Sciences 25, no. 14 (2024): 7616. 10.3390/ijms25147616 PMC11277246 39062859

[140] B. M. Moni , Y. Sakurai , and J. Yasuda , “Ebola Virus Gp Activates Endothelial Cells via Host Cytoskeletal Signaling Factors,” Viruses 14, no. 1 (2022): 142. 10.3390/v14010142 PMC8781776 35062347

[141] C. Muñoz‐Fontela and A. K. McElroy , “Ebola Virus Disease in Humans: Pathophysiology and Immunity,” Current Topics in Microbiology and Immunology 411 (2017): 141–169. 10.1007/82_2017_11 PMC7122202 28653186

[142] F. Y. Tso , G. Kang , E. H. Kwon , et al., “Brain Is a Potential Sanctuary for Subtype C HIV‐1 Irrespective of Art Treatment Outcome,” PLoS ONE 13, no. 7 (2018): e0201325. 10.1371/journal.pone.0201325 PMC6057662 30040863

[143] A. Mihalić , J. Železnjak , B. Lisnić , S. Jonjić , V. Juranić Lisnić , and I. Brizić , “Immune Surveillance of Cytomegalovirus in Tissues,” Cellular & Molecular Immunology 21, no. 9 (2024): 959–981. 10.1038/s41423-024-01186-2 PMC11364667 39134803

[144] J. de Melo Silva , R. Pinheiro‐Silva , A. Dhyani , and G. S. Pontes , “Cytomegalovirus and Epstein‐Barr Infections: Prevalence and Impact on Patients With Hematological Diseases,” BioMed Research International 2020 (2020): 1627824. 10.1155/2020/1627824 PMC7605947 33163531

[145] A. Y. Chen , W. Guan , S. Lou , Z. Liu , S. Kleiboeker , and J. Qiu , “Role of Erythropoietin Receptor Signaling in Parvovirus B19 Replication in Human Erythroid Progenitor Cells,” Journal of Virology 84, no. 23 (2010): 12385–12396. 10.1128/JVI.01229-10 PMC2976398 20861249

[146] Y. Luo , S. Lou , X. Deng , et al., “Parvovirus B19 Infection of Human Primary Erythroid Progenitor Cells Triggers ATR‐CHK1 Signaling, Which Promotes B19 Virus Replication,” Journal of Virology 85, no. 16 (2011): 8046–8055. 10.1128/JVI.00831-11 PMC3147961 21680529

[147] J. G. Shantha , I. Crozier , and S. Yeh , “An Update on Ocular Complications of Ebola Virus Disease,” Current Opinion in Ophthalmology 28, no. 6 (2017): 600–606. 10.1097/ICU.0000000000000426 PMC5988239 28872492

[148] S. Gupta and S. Pellett , “Recent Developments in Vaccine Design: From Live Vaccines to Recombinant Toxin Vaccines,” Toxins 15, no. 9 (2023): 563. 10.3390/toxins15090563 PMC10536331 37755989

[149] M. Ghattas , G. Dwivedi , M. Lavertu , and M. G. Alameh , “Vaccine Technologies and Platforms for Infectious Diseases: Current Progress, Challenges, and Opportunities,” Vaccines 9, no. 12 (2021): 1490. 10.3390/vaccines9121490 PMC8708925 34960236

[150] M. N. Sithole , M. R. Khan , H. A. Mohammed , R. A. Khan , K. Naik , and Y. E. Choonara , “A Systematic Review on Vaccine Developmental Approaches: Evaluating Efficacy, and Addressing Challenges of Infectious Diseases in the Post‐COVID‐19 Era,” Virus Research 367 (2026): 199720. 10.1016/j.virusres.2026.199720 PMC13092616 41932444

[151] C. R. Wolfe , J. Cohen , K. Mahoney , et al., “Safety and Efficacy of Pemivibart, a Long‐Acting Monoclonal Antibody, for Prevention of Symptomatic Covid‐19: Interim Results From a Phase 3 Randomized Clinical Trial (Canopy),” Clinical Infectious Diseases 81, no. 3 (2025): 439–450. 10.1093/cid/ciaf265 PMC12497959 40410927

[152] V. Soriano , A. Treviño , C. de Mendoza , et al., “Pre‐Exposure Prophylaxis for Viral Infections Other Than HIV,” Le Infezioni in Medicina 30, no. 3 (2022): 362–371. 10.53854/liim-3003-5 PMC9448312 36148176

[153] H. Chi , M. Pepper , and P. G. Thomas , “Principles and Therapeutic Applications of Adaptive Immunity,” Cell 187, no. 9 (2024): 2052–2078. 10.1016/j.cell.2024.03.037 PMC11177542 38670065

[154] S. M. Kang and R. W. Compans , “Host Responses From Innate to Adaptive Immunity After Vaccination: Molecular and Cellular Events,” Molecules and Cells 27, no. 1 (2009): 5–14. 10.1007/s10059-009-0015-1 PMC6280669 19214429

[155] B. Pulendran and R. Ahmed , “Immunological Mechanisms of Vaccination,” Nature Immunology 12, no. 6 (2011): 509–517. 10.1038/ni.2039 PMC3253344 21739679

[156] S. Crotty , “Immunological Memory to Vaccines,” Immunity 59, no. 4 (2026): 813–832. 10.1016/j.immuni.2026.02.019 PMC13124080 41985440

[157] I. N. Weerarathna , E. S. Doelakeh , L. Kiwanuka , P. Kumar , and S. Arora , “Prophylactic and Therapeutic Vaccine Development: Advancements and Challenges,” Molecular Biomedicine 5, no. 1 (2024): 57. 10.1186/s43556-024-00222-x PMC11554974 39527305

[158] A. J. Pollard and E. M. Bijker , “A Guide to Vaccinology: From Basic Principles to New Developments,” Nature Reviews Immunology 21, no. 2 (2021): 83–100. 10.1038/s41577-020-00479-7 PMC7754704 33353987

[159] Y. Hou , M. Chen , Y. Bian , X. Zheng , R. Tong , and X. Sun , “Advanced Subunit Vaccine Delivery Technologies: From Vaccine Cascade Obstacles to Design Strategies,” Acta Pharmaceutica Sinica B 13, no. 8 (2023): 3321–3338. 10.1016/j.apsb.2023.01.006 PMC10465871 37655334

[160] J. Tang , M. A. Amin , and J. L. Campian , “Past, Present, and Future of Viral Vector Vaccine Platforms: A Comprehensive Review,” Vaccines 13, no. 5 (2025): 524. 10.3390/vaccines13050524 PMC12115715 40432133

[161] C. Wang and F. Yuan , “A Comprehensive Comparison of DNA and RNA Vaccines,” Advanced Drug Delivery Reviews 210 (2024): 115340. 10.1016/j.addr.2024.115340 PMC11181159 38810703

[162] I. Papadatou and A. Michos , “Advances in Biotechnology and the Development of Novel Human Vaccines,” Vaccines 13, no. 9 (2025): 989. 10.3390/vaccines13090989 PMC12474200 41012191

[163] R. Rappuoli , M. Pizza , G. Del Giudice , and E. De Gregorio , “Vaccines, New Opportunities for a New Society,” Proceedings of the National Academy of Sciences of the United States of America 111, no. 34 (2014): 12288–12293. 10.1073/pnas.1402981111 PMC4151714 25136130

[164] P. A. van der Ley , A. Zariri , E. van Riet , D. Oosterhoff , and C. P. Kruiswijk , “An Intranasal Omv‐Based Vaccine Induces High Mucosal and Systemic Protecting Immunity Against a SARS‐CoV‐2 Infection,” Frontiers in Immunology 12 (2021): 781280. 10.3389/fimmu.2021.781280 PMC8721663 34987509

[165] T. Gupalova , G. Leontieva , T. Kramskaya , K. Grabovskaya , E. Kuleshevich , and A. Suvorov , “Development of Experimental Pneumococcal Vaccine for Mucosal Immunization,” PLoS ONE 14, no. 6 (2019): e0218679. 10.1371/journal.pone.0218679 PMC6599147 31251760

[166] E. C. Lavelle and R. W. Ward , “Mucosal Vaccines — Fortifying the Frontiers,” Nature Reviews Immunology 22, no. 4 (2022): 236–250. 10.1038/s41577-021-00583-2 PMC8312369 34312520

[167] S. Kumar , R. Sunagar , and E. J. Gosselin , “Preclinical Efficacy of a Trivalent Human Fcγri‐Targeted Adjuvant‐Free Subunit Mucosal Vaccine Against Pulmonary Pneumococcal Infection,” Vaccines 8, no. 2 (2020): 193. 10.3390/vaccines8020193 PMC7349865 32340134

[168] K. S. Corbett , D. K. Edwards , S. R. Leist , et al., “SARS‐CoV‐2 mRNA Vaccine Design Enabled by Prototype Pathogen Preparedness,” Nature 586, no. 7830 (2020): 567–571. 10.1038/s41586-020-2622-0 PMC7581537 32756549

[169] N. A. Khan , H. Al‐Thani , and A. El‐Menyar , “The Emergence of New SARS‐CoV‐2 Variant (Omicron) and Increasing Calls for COVID‐19 Vaccine Boosters‐The Debate Continues,” Travel Medicine and Infectious Disease 45 (2022): 102246. 10.1016/j.tmaid.2021.102246 PMC8687713 34942376

[170] P. T. Heath , E. P. Galiza , D. N. Baxter , et al., “Safety and Efficacy of Nvx‐Cov2373 COVID‐19 Vaccine,” New England Journal of Medicine 385, no. 13 (2021): 1172–1183. 10.1056/NEJMoa2107659 PMC8262625 34192426

[171] M. N. Vu , H. G. Kelly , S. J. Kent , and A. K. Wheatley , “Current and Future Nanoparticle Vaccines for Covid‐19,” EBioMedicine 74 (2021): 103699. 10.1016/j.ebiom.2021.103699 PMC8602808 34801965

[172] Z. Ghaemmaghamian , R. Zarghami , G. Walker , E. O'Reilly , and A. Ziaee , “Stabilizing Vaccines via Drying: Quality by Design Considerations,” Advanced Drug Delivery Reviews 187 (2022): 114313. 10.1016/j.addr.2022.114313 35597307

[173] D. Hosangadi , E. K. Martin , M. Watson , R. Bruns , and N. Connell , “Supporting Use of Thermostable Vaccines During Public Health Emergencies: Considerations and Recommendations for the Future,” Vaccine 39, no. 48 (2021): 6972–6974. 10.1016/j.vaccine.2021.10.065 PMC8575557 34763948

[174] Y. Wan , V. Gupta , C. Bird , et al., “Formulation Development and Improved Stability of a Combination Measles and Rubella Live‐Viral Vaccine Dried for Use in the Nanopatch^TM^ Microneedle Delivery System,” Human Vaccines & Immunotherapeutics 17, no. 8 (2021): 2501–2516. 10.1080/21645515.2021.1887692 PMC8475600 33957843

[175] S. A. Plotkin , “Recent Updates on Correlates of Vaccine‐Induced Protection,” Frontiers in Immunology 13 (2022): 1081107. 10.3389/fimmu.2022.1081107 PMC9912984 36776392

[176] S. K. Davis , F. Jia , Q. G. Wright , et al., “Defining Correlates of Protection for Mammalian Livestock Vaccines Against High‐Priority Viral Diseases,” Frontiers in Immunology 15 (2024): 1397780. 10.3389/fimmu.2024.1397780 PMC11294087 39100679

[177] M. Sadarangani , A. Marchant , and T. R. Kollmann , “Immunological Mechanisms of Vaccine‐Induced Protection Against Covid‐19 in Humans,” Nature Reviews Immunology 21, no. 8 (2021): 475–484. 10.1038/s41577-021-00578-z PMC8246128 34211186

[178] P. B. Gilbert , D. C. Montefiori , A. B. McDermott , et al., “Immune Correlates Analysis of the mRNA‐1273 Covid‐19 Vaccine Efficacy Clinical Trial,” Science 375, no. 6576 (2022): 43–50. 10.1126/science.abm3425 PMC9017870 34812653

[179] D. S. Khoury , D. Cromer , A. Reynaldi , et al., “Neutralizing Antibody Levels Are Highly Predictive of Immune Protection From Symptomatic SARS‐CoV‐2 Infection,” Nature Medicine 27, no. 7 (2021): 1205–1211. 10.1038/s41591-021-01377-8 34002089

[180] K. Sun , J. N. Bhiman , S. Tempia , et al., “SARS‐CoV‐2 Correlates of Protection From Infection Against Variants of Concern,” Nature Medicine 30, no. 10 (2024): 2805–2812. 10.1038/s41591-024-03131-2 PMC11533127 39060660

[181] T. Koch , S. C. Mellinghoff , P. Shamsrizi , M. M. Addo , and C. Dahlke , “Correlates of Vaccine‐Induced Protection Against SARS‐CoV‐2,” Vaccines 9, no. 3 (2021): 238. 10.3390/vaccines9030238 PMC8035658 33801831

[182] M. Federico , “Virus‐Induced CD8+ T‐Cell Immunity and Its Exploitation to Contain the SARS‐CoV‐2 Pandemic,” Vaccines 9, no. 8 (2021): 922. 10.3390/vaccines9080922 PMC8402519 34452047

[183] S. Sakabe , B. M. Sullivan , J. N. Hartnett , et al., “Analysis of CD8(+) T Cell Response During the 2013–2016 Ebola Epidemic in West Africa,” Proceedings of the National Academy of Sciences of the United States of America 115, no. 32 (2018): E7578–E7586. 10.1073/pnas.1806200115 PMC6094108 30038008

[184] X. Zhang , J. Zhang , S. Chen , et al., “Progress and Challenges in the Clinical Evaluation of Immune Responses to Respiratory Mucosal Vaccines,” Expert Review of Vaccines 23, no. 1 (2024): 362–370. 10.1080/14760584.2024.2326094 38444382

[185] S. Younas , S. Farooq , S. Sahu , R. P. Mwita , and Ö. Özdemir , “Next‐Generation Mucosal Vaccines for Respiratory Viruses: Immunological Correlates, Platform Design and Clinical Translation,” World Journal of Virology 15, no. 1 (2026): 116939. 10.5501/wjv.v15.i1.116939 PMC13010934 41884458

[186] P. B. Gilbert , Y. Fong , N. S. Hejazi , et al., “Four Statistical Frameworks for Assessing an Immune Correlate of Protection (Surrogate Endpoint) From a Randomized, Controlled, Vaccine Efficacy Trial,” Vaccine 42, no. 9 (2024): 2181–2190. 10.1016/j.vaccine.2024.02.071 PMC10999339 38458870

[187] S. Feng , D. J. Phillips , T. White , et al., “Correlates of Protection Against Symptomatic and Asymptomatic SARS‐CoV‐2 Infection,” Nature Medicine 27, no. 11 (2021): 2032–2040. 10.1038/s41591-021-01540-1 PMC8604724 34588689

[188] S. Urdy , M. Hanke , A. I. Toledo , et al., “Multi‐Strain Modeling of Influenza Vaccine Effectiveness in Older Adults and Its Dependence on Antigenic Distance,” Scientific Reports 14, no. 1 (2024): 27190. 10.1038/s41598-024-72716-1 PMC11549341 39516205

[189] J. W. Yewdell , “Antigenic Drift: Understanding Covid‐19,” Immunity 54, no. 12 (2021): 2681–2687. 10.1016/j.immuni.2021.11.016 PMC8669911 34910934

[190] E. Fossum , A. Rohringer , T. Aune , K. M. Rydland , K. Bragstad , and O. Hungnes , “Antigenic Drift and Immunity Gap Explain Reduction in Protective Responses Against Influenza A(H1N1)Pdm09 and A(H3N2) Viruses During the COVID‐19 Pandemic: A Cross‐Sectional Study of Human Sera Collected in 2019, 2021, 2022, and 2023,” Virology Journal 21, no. 1 (2024): 57. 10.1186/s12985-024-02326-w PMC10916265 38448981

[191] D. P. Maurer , M. Vu , and A. G. Schmidt , “Antigenic Drift Expands Influenza Viral Escape Pathways From Recalled Humoral Immunity,” Immunity 58, no. 3 (2025): 716–727.e6. 10.1016/j.immuni.2025.02.006 PMC11906258 40023162

[192] R. Steventon , L. Stolle , and C. P. Thompson , “How Broadly Neutralising Antibodies Are Redefining Immunity to Influenza,” Antibodies 14, no. 1 (2025): 4. 10.3390/antib14010004 PMC11755579 39846612

[193] K. E. C. Ainslie , M. Haber , and W. A. Orenstein , “Challenges in Estimating Influenza Vaccine Effectiveness,” Expert Review of Vaccines 18, no. 6 (2019): 615–628. 10.1080/14760584.2019.1622419 PMC6594904 31116070

[194] W. Dejnirattisai , R. H. Shaw , P. Supasa , et al., “Reduced Neutralisation of SARS‐CoV‐2 Omicron B.1.1.529 Variant by Post‐Immunisation Serum,” The Lancet 399, no. 10321 (2022): 234–236. 10.1016/S0140-6736(21)02844-0 PMC8687667 34942101

[195] H. Ke , M. R. Chang , and W. A. Marasco , “Immune Evasion of SARS‐CoV‐2 Omicron Subvariants,” Vaccines 10, no. 9 (2022): 1545. 10.3390/vaccines10091545 PMC9501521 36146623

[196] S. M. King , S. P. Bryan , S. P. Hilchey , J. Wang , and M. S. Zand , “First Impressions Matter: Immune Imprinting and Antibody Cross‐Reactivity in Influenza and SARS‐CoV‐2,” Pathogens 12, no. 2 (2023): 169. 10.3390/pathogens12020169 PMC9967769 36839441

[197] M. Maltseva , A. Keeshan , C. Cooper , and M. A. Langlois , “Immune Imprinting: The Persisting Influence of the First Antigenic Encounter With Rapidly Evolving Viruses,” Human Vaccines & Immunotherapeutics 20, no. 1 (2024): 2384192. 10.1080/21645515.2024.2384192 PMC11328881 39149872

[198] S. M. Reincke , H. Prüss , I. A. Wilson , and J. Kreye , “Antigenic Imprinting in SARS‐CoV‐2,” Clinical and Translational Medicine 12, no. 7 (2022): e923. 10.1002/ctm2.923 PMC9270639 35808842

[199] W. Yang , K. Jin , W. Li , et al., “Mapping Immune Imprinting Zones Enables Predictive Vaccination Optimization,” Cell reports 45, no. 4 (2026): 117271. 10.1016/j.celrep.2026.117271 41999605

[200] N. E. MacDonald , “Vaccine Hesitancy: Definition, Scope and Determinants,” Vaccine 33, no. 34 (2015): 4161–4164. 10.1016/j.vaccine.2015.04.036 25896383

[201] G. H. Shah and T. H. Nguyen , “Vaccine Hesitancy Through a Global Lens: Cross‐Cultural Evidence From a Special Issue,” Vaccines 13, no. 5 (2025): 529. 10.3390/vaccines13050529 PMC12115658 40432138

[202] P. M. Galagali , A. A. Kinikar , and V. S. Kumar , “Vaccine Hesitancy: Obstacles and Challenges,” Current Pediatrics Reports 10, no. 4 (2022): 241–248. 10.1007/s40124-022-00278-9 PMC9546747 36245801

[203] R. Biswas , A. Roy , T. Kayal , et al., “Waning Immunity and the Future of Booster Vaccination Strategies in Global Vaccine Programs Post COVID‐19,” Human Vaccines & Immunotherapeutics 22, no. 1 (2026): 2626088. 10.1080/21645515.2026.2626088 PMC12893694 41656902

[204] B. Resch , “Product Review on the Monoclonal Antibody Palivizumab for Prevention of Respiratory Syncytial Virus Infection,” Human Vaccines & Immunotherapeutics 13, no. 9 (2017): 2138–2149. 10.1080/21645515.2017.1337614 PMC5612471 28605249

[205] E. Bizot , V. Portet Sulla , C. Vauloup‐Fellous , M.‐A. Rameix‐Welti , V. Gajdos , and N. Seddiki , “Prophylactic Monoclonal Antibodies Against Respiratory Syncytial Virus in Early Life: An in‐Depth Review of Mechanisms of Action, Failure Factors, and Future Perspectives,” Pediatric Allergy and Immunology 36, no. 12 (2025): e70257. 10.1111/pai.70257 PMC12679812 41347702

[206] C. Wegzyn , L. K. Toh , G. Notario , et al., “Safety and Effectiveness of Palivizumab in Children at High Risk of Serious Disease due to Respiratory Syncytial Virus Infection: A Systematic Review,” Infectious Diseases and Therapy 3, no. 2 (2014): 133–158. 10.1007/s40121-014-0046-6 PMC4269625 25297809

[207] P. P. Wu and F. R. Ding , “Administration of Nirsevimab for RSV Prophylaxis in Infants: A Comprehensive Review,” Vaccines 13, no. 5 (2025): 470. 10.3390/vaccines13050470 PMC12115584 40432081

[208] N. O. Elhassan , M. E. Sorbero , C. B. Hall , T. P. Stevens , and A. W. Dick , “Cost‐Effectiveness Analysis of Palivizumab in Premature Infants Without Chronic Lung Disease,” Archives of Pediatrics & Adolescent Medicine 160, no. 10 (2006): 1070. 10.1001/archpedi.160.10.1070 17018467

[209] Q. Zhu , J. S. McLellan , N. L. Kallewaard , et al., “A Highly Potent Extended Half‐Life Antibody as a Potential RSV Vaccine Surrogate for all Infants,” Science Translational Medicine 9, no. 388 (2017): eaaj1928. 10.1126/scitranslmed.aaj1928 28469033

[210] M. P. Griffin , Y. Yuan , T. Takas , et al., “Single‐Dose Nirsevimab for Prevention of RSV in Preterm Infants,” New England Journal of Medicine 383, no. 5 (2020): 415–425. 10.1056/NEJMoa1913556 32726528

[211] K. Kelleher , N. Subramaniam , and S. B. Drysdale , “The Recent Landscape of RSV Vaccine Research,” Therapeutic Advances in Vaccines and Immunotherapy 13 (2025): 25151355241310601. 10.1177/25151355241310601 PMC11724408 39802673

[212] M. Sánchez Luna , M. L. Couce Pico , and B. Fernández Colomer , “Recommendations of the Spanish Society of Neonatology for the Prevention of Severe Respiratory Syncytial Virus Infections With Nirsevimab, for the 2024–2025 Season,” Anales de Pediatra (English Edition) 101, no. 5 (2024): 364–366. 10.1016/j.anpede.2024.09.010 39455352

[213] J. M. Jones , K. E. Fleming‐Dutra , M. M. Prill , et al., “Use of Nirsevimab for the Prevention of Respiratory Syncytial Virus Disease Among Infants and Young Children: Recommendations of the Advisory Committee on Immunization Practices — United States, 2023,” MMWR Morbidity and Mortality Weekly Report 72, no. 34 (2023): 920–925. 10.15585/mmwr.mm7234a4 PMC10468217 37616235

[214] M. S. Askari , K. Oliver , D. Benkel , et al., “Notes From the Field: Rollout of Nirsevimab to Protect Infants and Young Children During the Respiratory Syncytial Virus Season — New York City, 2023–2024,” MMWR Morbidity and Mortality Weekly Report 73, no. 48 (2024): 1107–1109. 10.15585/mmwr.mm7348a4 PMC11620337 39636800

[215] D. Focosi and A. Casadevall , “A Critical Analysis of the Use of Cilgavimab Plus Tixagevimab Monoclonal Antibody Cocktail (Evusheld™) for COVID‐19 Prophylaxis and Treatment,” Viruses 14, no. 9 (2022): 1999. 10.3390/v14091999 PMC9505619 36146805

[216] S. Ko , S. Park , M. H. Sohn , et al., “An Fc Variant With Two Mutations Confers Prolonged Serum Half‐Life and Enhanced Effector Functions on Igg Antibodies,” Experimental & Molecular Medicine 54, no. 11 (2022): 1850–1861. 10.1038/s12276-022-00870-5 PMC9628495 36319752

[217] Y.‐M. Loo , P. M. McTamney , R. H. Arends , et al., “The SARS‐CoV‐2 Monoclonal Antibody Combination, AZD7442, Is Protective in Nonhuman Primates and Has an Extended Half‐Life in Humans,” Science Translational Medicine 14, no. 635 (2022): eabl8124. 10.1126/scitranslmed.abl8124 PMC8939769 35076282

[218] K. Akinosoglou , E.‐A. Rigopoulos , G. Kaiafa , et al., “Tixagevimab/Cilgavimab in SARS‐CoV‐2 Prophylaxis and Therapy: A Comprehensive Review of Clinical Experience,” Viruses 15, no. 1 (2022): 118. 10.3390/v15010118 PMC9866621 36680160

[219] E. Schiaroli , A. Gidari , G. Brachelente , et al., “Impaired Neutralizing Antibody Efficacy of Tixagevimab‐Cilgavimab 150+150 mg as Pre‐Exposure Prophylaxis Against Omicron BA.5. A Real‐World Experience in Booster Vaccinated Immunocompromised Patients,” Journal of Clinical Virology 168 (2023): 105584. 10.1016/j.jcv.2023.105584 37778220

[220] A. Zhang , H. D. Stacey , M. R. D'Agostino , Y. Tugg , A. Marzok , and M. S. Miller , “Beyond Neutralization: Fc‐Dependent Antibody Effector Functions in SARS‐CoV‐2 Infection,” Nature Reviews Immunology 23, no. 6 (2023): 381–396. 10.1038/s41577-022-00813-1 PMC9761659 36536068

[221] M. J. Levin , A. Ustianowski , S. De Wit , et al., “Intramuscular AZD7442 (Tixagevimab–Cilgavimab) for Prevention of COVID‐19,” New England Journal of Medicine 386, no. 23 (2022): 2188–2200. 10.1056/NEJMoa2116620 PMC9069994 35443106

[222] A. Razzaq , C. Disoma , S. Iqbal , et al., “Genomic Epidemiology and Evolutionary Dynamics of the Omicron Variant of SARS‐CoV‐2 During the Fifth Wave of Covid‐19 in Pakistan,” Frontiers in Cellular and Infection Microbiology 14 (2024): 1484637. 10.3389/fcimb.2024.1484637 PMC11534695 39502171

[223] Y. Fan , X. Li , L. Zhang , S. Wan , L. Zhang , and F. Zhou , “SARS‐CoV‐2 Omicron Variant: Recent Progress and Future Perspectives,” Signal Transduction and Targeted Therapy 7, no. 1 (2022): 141. 10.1038/s41392-022-00997-x PMC9047469 35484110

[224] J. Yang , S. Hyeon , J. Y. Baek , et al., “Loss of Neutralizing Activity of Tixagevimab/Cilgavimab (Evusheld™) Against Omicron Bn.1, a Dominant Circulating Strain Following BA.5 During the Seventh Domestic Outbreak in Korea in Early 2023,” Journal of Korean Medical Science 38, no. 27 (2023): e205. 10.3346/jkms.2023.38.e205 PMC10332950 37431539

[225] M. Sun , Y. Li , Z. Yuan , et al., “Vrc01 Antibody Protects Against Vaginal and Rectal Transmission of Human Immunodeficiency Virus 1 in HU‐BLT Mice,” Archives of Virology 161, no. 9 (2016): 2449–2455. 10.1007/s00705-016-2942-4 PMC4988922 27343044

[226] M. P. Lemos , R. D. Astronomo , Y. Huang , et al., “Enhanced and Sustained Biodistribution of HIV‐1 Neutralizing Antibody VRC01LS in Human Genital and Rectal Mucosa,” Nature Communications 15, no. 1 (2024): 10332. 10.1038/s41467-024-54580-9 PMC11604655 39609400

[227] V. V. A. Mallajosyula , M. Citron , F. Ferrara , et al., “Influenza Hemagglutinin Stem‐Fragment Immunogen Elicits Broadly Neutralizing Antibodies and Confers Heterologous Protection,” Proceedings of the National Academy of Sciences of the United States of America 111, no. 25 (2014): E2514–E2523. 10.1073/pnas.1402766111 PMC4078824 24927560

[228] L. Corey , P. B. Gilbert , M. Juraska , et al., “Two Randomized Trials of Neutralizing Antibodies to Prevent HIV‐1 Acquisition,” New England Journal of Medicine 384, no. 11 (2021): 1003–1014. 10.1056/NEJMoa2031738 PMC8189692 33730454

[229] G. Pantaleo , B. Correia , C. Fenwick , V. S. Joo , and L. Perez , “Antibodies to Combat Viral Infections: Development Strategies and Progress,” Nature Reviews Drug Discovery 21, no. 9 (2022): 676–696. 10.1038/s41573-022-00495-3 PMC9207876 35725925

[230] P. Y. Atanasov , M. G. Moneva‐Sakelarieva , Y. A. Kobakova , et al., “Monoclonal Antibodies: Their Place in Applied Medicine and Their Role in the Newest Infectious Disease—History, Present, and Future. Literature Review,” PHARMACIA 72 (2025): 1–18.

[231] E. M. Kikwango , P. Z. Akilimali , and N. T. Tran , “Impact of Most Promising Ebola Therapies on Survival: A Secondary Analysis During the Tenth Outbreak in the Democratic Republic of Congo,” Virology Journal 22, no. 1 (2025): 144. 10.1186/s12985-025-02766-y PMC12079879 40375337

[232] P. L. Iversen , C. D. Kane , X. Zeng , et al., “Recent Successes in Therapeutics for Ebola Virus Disease: No Time for Complacency,” The Lancet Infectious Diseases 20, no. 9 (2020): e231–e237. 10.1016/S1473-3099(20)30282-6 PMC7302789 32563280

[233] A. Markham , “Regn‐Eb3: First Approval,” Drugs 81, no. 1 (2021): 175–178. 10.1007/s40265-020-01452-3 PMC7799152 33432551

[234] E. Taki , R. Ghanavati , T. Navidifar , S. Dashtbin , M. Heidary , and M. Moghadamnia , “Ebanga™: The Most Recent FDA‐Approved Drug for Treating Ebola,” Frontiers in Pharmacology 14 (2023): 1083429. 10.3389/fphar.2023.1083429 PMC10032372 36969842

[235] E. E. H. Tran , E. A. Nelson , P. Bonagiri , et al., “Mapping of Ebolavirus Neutralization by Monoclonal Antibodies in the ZMapp Cocktail Using Cryo‐Electron Tomography and Studies of Cellular Entry,” Journal of Virology 90, no. 17 (2016): 7618–7627. 10.1128/JVI.00406-16 PMC4988163 27279622

[236] V. Rayaprolu , B. O. Fulton , A. Rafique , et al., “Structure of the Inmazeb Cocktail and Resistance to Ebola Virus Escape,” Cell Host & Microbe 31, no. 2 (2023): 260–272.e7. 10.1016/j.chom.2023.01.002 PMC10375381 36708708

[237] M. Dougan , A. Nirula , M. Azizad , et al., “Bamlanivimab Plus Etesevimab in Mild or Moderate COVID‐19,” New England Journal of Medicine 385, no. 15 (2021): 1382–1392. 10.1056/NEJMoa2102685 PMC8314785 34260849

[238] R. L. Gottlieb , A. Nirula , P. Chen , et al., “Effect of Bamlanivimab as Monotherapy or in Combination With Etesevimab on Viral Load in Patients With Mild to Moderate COVID‐19,” Jama 325, no. 7 (2021): 632. 10.1001/jama.2021.0202 PMC7821080 33475701

[239] E. D. Deeks , “Casirivimab/Imdevimab: First Approval,” Drugs 81, no. 17 (2021): 2047–2055. 10.1007/s40265-021-01620-z PMC8556815 34716907

[240] Y. A. Heo , “Sotrovimab: First Approval,” Drugs 82, no. 4 (2022): 477–484. 10.1007/s40265-022-01690-7 PMC8919156 35286623

[241] G. A. Herman , M. P. O'Brien , E. Forleo‐Neto , et al., “Efficacy and Safety of a Single Dose of Casirivimab and Imdevimab for the Prevention of COVID‐19 Over an 8‐Month Period: A Randomised, Double‐Blind, Placebo‐Controlled Trial,” The Lancet Infectious Diseases 22, no. 10 (2022): 1444–1454. 10.1016/S1473-3099(22)00416-9 PMC9255947 35803290

[242] F. Isa , A. M. Gonzalez Ortiz , J. Meyer , et al., “Effect of Timing of Casirivimab and Imdevimab Administration Relative to mRNA‐1273 Covid‐19 Vaccination on Vaccine‐Induced SARS‐CoV‐2 Neutralising Antibody Responses: A Prospective, Open‐Label, Phase 2, Randomised Controlled Trial,” The Lancet Infectious Diseases 25, no. 1 (2025): 52–67. 10.1016/S1473-3099(24)00421-3 39236733

[243] Q. Chen , W. Yin , Z. Zhu , and C. Wang , “The Clinical Advantages of Anti‐Rabies Monoclonal Antibodies in Post‐Exposure Prophylaxis—Worldwide, 2016–2025,” China CDC Weekly 7, no. 39 (2025): 1258–1262. 10.46234/ccdcw2025.212 PMC12518957 41098348

[244] A. D. Kaye , D. M. Perilloux , E. Field , et al., “Rabies Vaccine for Prophylaxis and Treatment of Rabies: A Narrative Review,” Cureus 16, no. 6 (2024): e62429. 10.7759/cureus.62429 PMC11248466 39011185

[245] R. Welliver , A. S. Monto , O. Carewicz , et al., “Effectiveness of Oseltamivir in Preventing Influenza in Household Contacts: A Randomized Controlled Trial,” Jama 285, no. 6 (2001): 748. 10.1001/jama.285.6.748 11176912

[246] R. J. Jackson , K. L. Cooper , P. Tappenden , et al., “Oseltamivir, Zanamivir and Amantadine in the Prevention of Influenza: A Systematic Review,” Journal of Infection 62, no. 1 (2011): 14–25. 10.1016/j.jinf.2010.10.003 20950645

[247] K. Fukao , Y. Ando , T. Noshi , et al., “Baloxavir Marboxil, a Novel Cap‐Dependent Endonuclease Inhibitor Potently Suppresses Influenza Virus Replication and Represents Therapeutic Effects in Both Immunocompetent and Immunocompromised Mouse Models,” PLoS ONE 14, no. 5 (2019): e0217307. 10.1371/journal.pone.0217307 PMC6527232 31107922

[248] F. G. Hayden , N. Sugaya , N. Hirotsu , et al., “Baloxavir Marboxil for Uncomplicated Influenza in Adults and Adolescents,” New England Journal of Medicine 379, no. 10 (2018): 913–923. 10.1056/NEJMoa1716197 30184455

[249] A. S. Monto , K. Kuhlbusch , C. Bernasconi , et al., “Efficacy of Baloxavir Treatment in Preventing Transmission of Influenza,” New England Journal of Medicine 392, no. 16 (2025): 1582–1593. 10.1056/NEJMoa2413156 40267424

[250] M. G. Ison , S. Portsmouth , Y. Yoshida , et al., “Early Treatment With Baloxavir Marboxil in High‐Risk Adolescent and Adult Outpatients With Uncomplicated Influenza (Capstone‐2): A Randomised, Placebo‐Controlled, Phase 3 Trial,” The Lancet Infectious Diseases 20, no. 10 (2020): 1204–1214. 10.1016/S1473-3099(20)30004-9 32526195

[251] J. M. Baeten , D. Donnell , N. R. Mugo , et al., “Single‐Agent Tenofovir Versus Combination Emtricitabine Plus Tenofovir for Pre‐Exposure Prophylaxis for HIV‐1 Acquisition: An Update of Data From a Randomised, Double‐Blind, Phase 3 Trial,” The Lancet Infectious Diseases 14, no. 11 (2014): 1055–1064. 10.1016/S1473-3099(14)70937-5 PMC4252589 25300863

[252] M. Mascolini , “Tenofovir Prep Reduces HIV Transmission Risk by up to 99%: Adherence Critical to Protection From HIV in Partners Prep Trial of TDF and TDF/FTC,” in Conference Reports for 19th Conference on Retroviruses and Opportunistic Infections, Seattle, WA (2012), https://www.Natap.Org/2012/Croi/Croi_10.Htm#:~:Text=among%20people%20randomized%20to%20take,%2c%20ahmed%20k%2c%20et%20al, March 5–8.

[253] D. T. Dimitrov , B. R. Mâsse , and D. Donnell , “Prep Adherence Patterns Strongly Affect Individual HIV Risk and Observed Efficacy in Randomized Clinical Trials,” JAIDS Journal of Acquired Immune Deficiency Syndromes 72, no. 4 (2016): 444–451. 10.1097/QAI.0000000000000993 PMC4925182 26990823

[254] E. Mahase , “HIV Prevention Injection Is More Effective Than Daily Pill, Trials Show,” BMJ (Clinical Research Ed) 371 (2020): m4433. 10.1136/bmj.m4433 33184095

[255] S. Delany‐Moretlwe , J. P. Hughes , P. Bock , et al., “Cabotegravir for the Prevention of HIV‐1 in Women: Results From Hptn 084, a Phase 3, Randomised Clinical Trial,” The Lancet 399, no. 10337 (2022): 1779–1789. 10.1016/S0140-6736(22)00538-4 PMC9077443 35378077

[256] O. Müller , G. Lu , P. Meissner , L. von Seidlein , A. Jahn , and O. Razum , “Achievements and Obstacles in the Late Phase of the Global Polio Eradication Initiative,” Deutsches Arzteblatt International 122, no. 14 (2025): 393–398. 10.3238/arztebl.m2025.0079 PMC12571390 40407715

[257] S. Bahl , S. Khanal , L. Sangal , S. Tabassum , K. Ungchusak , and J. Andrus , “Measles and Rubella Elimination: Protecting Children Through Immunization in South‐East Asia Region (SEAR),” The Lancet Regional Health—Southeast Asia 18 (2023): 100303. 10.1016/j.lansea.2023.100303 PMC10667283 38028171

[258] N. E. Dean and I. M. Longini , “The Ring Vaccination Trial Design for the Estimation of Vaccine Efficacy and Effectiveness During Infectious Disease Outbreaks,” Clinical Trials 19, no. 4 (2022): 402–406. 10.1177/17407745211073594 PMC9300768 35057647

[259] N. Rupani , M. E. Ngole , J. A. Lee , et al., “Effect of Recombinant Vesicular Stomatitis Virus–Zaire Ebola Virus Vaccination on Ebola Virus Disease Illness and Death, Democratic Republic of the Congo,” Emerging Infectious Diseases 28, no. 6 (2022): 1180–1188. 10.3201/eid2806.212223 PMC9155898 35608607

[260] A. M. Henao‐Restrepo , A. Camacho , I. M. Longini , et al., “Efficacy and Effectiveness of an RVSV‐Vectored Vaccine in Preventing Ebola Virus Disease: Final Results From the Guinea Ring Vaccination, Open‐Label, Cluster‐Randomised Trial (Ebola Ça Suffit!),” The Lancet 389, no. 10068 (2017): 505–518. 10.1016/S0140-6736(16)32621-6 PMC5364328 28017403

[261] J.‐J. Muyembe , H. Pan , R. Peto , et al., “Ebola Outbreak Response in the DRC With RVSV‐ZEBOV‐GP Ring Vaccination,” New England Journal of Medicine 391, no. 24 (2024): 2327–2336. 10.1056/NEJMoa1904387 39693543

[262] C. Indriani , W. Tantowijoyo , E. Rancès , et al., “Reduced Dengue Incidence Following Deployments of Wolbachia‐Infected Aedes Aegypti in Yogyakarta, Indonesia: A Quasi‐Experimental Trial Using Controlled Interrupted Time Series Analysis,” Gates Open Research 4 (2020): 50. 10.12688/gatesopenres.13122.1 PMC7403856 32803130

[263] A. Utarini , C. Indriani , R. A. Ahmad , et al., “Efficacy of Wolbachia‐Infected Mosquito Deployments for the Control of Dengue,” New England Journal of Medicine 384, no. 23 (2021): 2177–2186. 10.1056/NEJMoa2030243 PMC8103655 34107180

[264] M. Li , T. Yang , M. Bui , et al., “Suppressing Mosquito Populations With Precision Guided Sterile Males,” Nature Communications 12, no. 1 (2021): 5374. 10.1038/s41467-021-25421-w PMC8433431 34508072

[265] Z. N. Adelman and Z. Tu , “Control of Mosquito‐Borne Infectious Diseases: Sex and Gene Drive,” Trends in Parasitology 32, no. 3 (2016): 219–229. 10.1016/j.pt.2015.12.003 PMC4767671 26897660

[266] T. Tharmalingam , X. Han , A. Wozniak , and L. Saward , “Polyclonal Hyper Immunoglobulin: A Proven Treatment and Prophylaxis Platform for Passive Immunization to Address Existing and Emerging Diseases,” Human Vaccines & Immunotherapeutics 18, no. 2 (2022): 1886560. 10.1080/21645515.2021.1886560 PMC9090292 34010089

[267] M. C. Tunis , M. I. Salvadori , V. Dubey , and O. Baclic , “Updated Naci Recommendations for Measles Post‐Exposure Prophylaxis,” Canada Communicable Disease Report = Releve Des Maladies Transmissibles Au Canada 44, no. 9 (2018): 226–230. 10.14745/ccdr.v44i09a07 PMC6449113 31015814

[268] S. A. Pergam , J. A. Englund , M. Kamboj , et al., “Preventing Measles in Immunosuppressed Cancer and Hematopoietic Cell Transplantation Patients: A Position Statement by the American Society for Transplantation and Cellular Therapy,” Biology of Blood and Marrow Transplantation 25, no. 11 (2019): e321–e330. 10.1016/j.bbmt.2019.07.034 31394271

[269] N. P. Nelson , T. V. Murphy , and B. J. McMahon , “Hepatitis a Vaccination for Post‐Exposure Prophylaxis in Persons Aged 40 Years and Older,” Vaccine 32, no. 25 (2014): 2939. 10.1016/j.vaccine.2014.01.086 PMC4643264 24530928

[270] D. Shouval , “Immunization Against Hepatitis A,” Cold Spring Harbor Perspectives in Medicine 9, no. 2 (2019): a031682. 10.1101/cshperspect.a031682 PMC6360863 29661808

[271] A. O. Kamphorst and R. Ahmed , “Cd4 T‐Cell Immunotherapy for Chronic Viral Infections and Cancer,” Immunotherapy 5, no. 9 (2013): 975–987. 10.2217/imt.13.91 PMC3864579 23998732

[272] R. Wahid , M. J. Cannon , and M. Chow , “Virus‐Specific CD4^+^ and CD8^+^ Cytotoxic T‐Cell Responses and Long‐Term T‐Cell Memory in Individuals Vaccinated Against Polio,” Journal of Virology 79, no. 10 (2005): 5988–5995. 10.1128/JVI.79.10.5988-5995.2005 PMC1091702 15857985

[273] C. Tøndel , S. Jenum , K. Tonby , et al., “SARS‐CoV‐2 T‐Cell Vaccine Vb10.2210 Induces Broad T‐Cell Responses in a Phase 1/2 Open‐Label Clinical Trial,” Vaccine 75 (2026): 128290. 10.1016/j.vaccine.2026.128290 41628524

[274] Z. Li , Y. Zhang , Y. Zheng , H. Wang , C. Xu , and Q. He , “Therapeutic Vaccines for Chronic Viral Infections: From Immune Modulation to Clinical Translation,” Vaccines 14, no. 6 (2026): 507. 10.3390/vaccines14060507 PMC13308487 42347630

[275] T. Hofer , M. Rossi , S. Carboni , et al., “Heterologous Prime‐Boost Vaccination With a Peptide‐Based Vaccine and Viral Vector Reshapes Dendritic Cell, CD4+ and CD8+ T Cell Phenotypes to Improve the Antitumor Therapeutic Effect,” Cancers 13, no. 23 (2021): 6107. 10.3390/cancers13236107 PMC8656755 34885215

[276] Z. Xu , A. Patel , N. J. Tursi , et al., “Harnessing Recent Advances in Synthetic DNA and Electroporation Technologies for Rapid Vaccine Development Against COVID‐19 and Other Emerging Infectious Diseases,” Frontiers in Medical Technology 2 (2020): 571030. 10.3389/fmedt.2020.571030 PMC8757735 35047878

[277] B. Sapkota , B. Saud , R. Shrestha , et al., “Heterologous Prime‐Boost Strategies for Covid‐19 Vaccines,” Journal of Travel Medicine 29, no. 3 (2022): taab191. 10.1093/jtm/taab191 PMC8754745 34918097

[278] T. Liu , H. Wang , Y. Zhao , Y. X. Wang , X. Xing , and P. Gao , “Drug Development for Chronic Hepatitis B Functional Cure: Recent Progress,” World Journal of Hepatology 17, no. 4 (2025): 105797. 10.4254/wjh.v17.i4.105797 PMC12038417 40308829

[279] H. Ma , T. H. Lim , A. Leerapun , et al., “Therapeutic Vaccine BRII‐179 Restores HBV‐Specific Immune Responses in Patients With Chronic HBV in a Phase IB/IIA Study,” JHEP Reports 3, no. 6 (2021): 100361. 10.1016/j.jhepr.2021.100361 PMC8502773 34661089

[280] A. Gaggar , C. Coeshott , D. Apelian , et al., “Safety, Tolerability and Immunogenicity of Gs‐4774, a Hepatitis B Virus‐Specific Therapeutic Vaccine, in Healthy Subjects: A Randomized Study,” Vaccine 32, no. 39 (2014): 4925–4931. 10.1016/j.vaccine.2014.07.027 25045824

[281] A. S. Lok , C. Q. Pan , S.‐H. B. Han , et al., “Randomized Phase II Study of GS‐4774 as a Therapeutic Vaccine in Virally Suppressed Patients With Chronic Hepatitis B,” Journal of Hepatology 65, no. 3 (2016): 509–516. 10.1016/j.jhep.2016.05.016 27210427

[282] Z. Chen and B. Julg , “Therapeutic Vaccines for the Treatment of HIV,” Translational Research 223 (2020): 61–75. 10.1016/j.trsl.2020.04.008 PMC8188575 32438074

[283] B. Mothe and C. Brander , “Prospects for Therapeutic T‐Cell Vaccine Strategies for HIV Cure,” Current Opinion in HIV and AIDS 20, no. 5 (2025): 463–471. 10.1097/COH.0000000000000965 PMC12337920 40638102

[284] B. Ma , B. Maraj , N. P. Tran , et al., “Emerging Human Papillomavirus Vaccines,” Expert Opinion on Emerging Drugs 17, no. 4 (2012): 469–492. 10.1517/14728214.2012.744393 PMC3786409 23163511

[285] C. L. Trimble , M. P. Morrow , K. A. Kraynyak , et al., “Safety, Efficacy, and Immunogenicity of VGX‐3100, a Therapeutic Synthetic DNA Vaccine Targeting Human Papillomavirus 16 and 18 E6 and E7 Proteins for Cervical Intraepithelial Neoplasia 2/3: A Randomised, Double‐Blind, Placebo‐Controlled Phase 2b Trial,” The Lancet 386, no. 10008 (2015): 2078–2088. 10.1016/S0140-6736(15)00239-1 PMC4888059 26386540

[286] J. M. Collins , J. M. Redman , and J. L. Gulley , “Combining Vaccines and Immune Checkpoint Inhibitors to Prime, Expand, and Facilitate Effective Tumor Immunotherapy,” Expert Review of Vaccines 17, no. 8 (2018): 697–705. 10.1080/14760584.2018.1506332 PMC8262093 30058393

[287] C. R. Maldini , G. I. Ellis , and J. L. Riley , “Car T Cells for Infection, Autoimmunity and Allotransplantation,” Nature Reviews Immunology 18, no. 10 (2018): 605–616. 10.1038/s41577-018-0042-2 PMC6505691 30046149

[288] C. Berger , C. J. Turtle , M. C. Jensen , and S. R. Riddell , “Adoptive Transfer of Virus‐Specific and Tumor‐Specific T Cell Immunity,” Current Opinion in Immunology 21, no. 2 (2009): 224–232. 10.1016/j.coi.2009.02.010 PMC2720155 19304470

[289] J. Lee , E. Ahn , H. T. Kissick , and R. Ahmed , “Reinvigorating Exhausted T Cells by Blockade of the PD‐1 Pathway,” Forum on Immunopathological Disease and Therapeutics 6, no. 1–2 (2015): 7–17. 10.1615/ForumImmunDisTher.2015014188 PMC5341794 28286692

[290] K. A. Hofmeyer , H. Jeon , and X. Zang , “The Pd‐1/Pd‐L1 (B7‐H1) Pathway in Chronic Infection‐Induced Cytotoxic T Lymphocyte Exhaustion,” Journal of Biomedicine and Biotechnology 2011 (2011): 451694. 10.1155/2011/451694 PMC3180079 21960736

[291] M. Karim , C. W. Lo , and S. Einav , “Preparing for the Next Viral Threat With Broad‐Spectrum Antivirals,” Journal of Clinical Investigation 133, no. 11 (2023): e170236. 10.1172/JCI170236 PMC10232003 37259914

[292] N. Matsunaga , “Emerging Host‐Targeted Antivirals for Respiratory Viruses: Antivirals Targeting Virus Elements Versus Host Mechanisms,” Next Research 5 (2026): 101311.

[293] S. Barik , “Inhibition of Viral RNA‐Dependent RNA Polymerases by Nucleoside Inhibitors: An Illustration of the Unity and Diversity of Mechanisms,” International Journal of Molecular Sciences 23, no. 20 (2022): 12649. 10.3390/ijms232012649 PMC9604226 36293509

[294] T. Xu and L. Zhang , “Current Understanding of Nucleoside Analogs Inhibiting the SARS‐CoV‐2 RNA‐Dependent RNA Polymerase,” Computational and Structural Biotechnology Journal 21 (2023): 4385–4394. 10.1016/j.csbj.2023.09.001 PMC10498173 37711189

[295] S. H. P. Moghadam , A. Sarkoohi , Z. Navidi , B. Amani , B. Amani , and S. Khorramnia , “Comparing the Effectiveness and Safety of Remdesivir and Molnupiravir in COVID‐19: A Systematic Review and Meta‐Analysis,” Immunity, Inflammation and Disease 13, no. 10 (2025): e70289. 10.1002/iid3.70289 PMC12531351 41103058

[296] L. Vangeel , W. Chiu , S. De Jonghe , et al., “Remdesivir, Molnupiravir and Nirmatrelvir Remain Active Against SARS‐CoV‐2 Omicron and Other Variants of Concern,” Antiviral Research 198 (2022): 105252. 10.1016/j.antiviral.2022.105252 PMC8785409 35085683

[297] S. Masyeni , M. Iqhrammullah , A. Frediansyah , et al., “Molnupiravir: A Lethal Mutagenic Drug Against Rapidly Mutating Severe Acute Respiratory Syndrome Coronavirus 2—A Narrative Review,” Journal of Medical Virology 94, no. 7 (2022): 3006–3016. 10.1002/jmv.27730 PMC9088670 35315098

[298] M. G. Santoro and E. Carafoli , “Remdesivir: From Ebola to COVID‐19,” Biochemical and Biophysical Research Communications 538 (2021): 145–150. 10.1016/j.bbrc.2020.11.043 PMC7836944 33388129

[299] C. J. Gordon , E. P. Tchesnokov , E. Woolner , et al., “Remdesivir Is a Direct‐Acting Antiviral That Inhibits RNA‐Dependent RNA Polymerase From Severe Acute Respiratory Syndrome Coronavirus 2 With High Potency,” Journal of Biological Chemistry 295, no. 20 (2020): 6785–6797. 10.1074/jbc.RA120.013679 PMC7242698 32284326

[300] B. Sama , B. Selisko , C. Falcou , et al., “The Effects of Remdesivir's Functional Groups on Its Antiviral Potency and Resistance Against the SARS‐CoV‐2 Polymerase,” Antiviral Research 232 (2024): 106034. 10.1016/j.antiviral.2024.106034 39510431

[301] F. G. Hayden and N. Shindo , “Influenza Virus Polymerase Inhibitors in Clinical Development,” Current Opinion in Infectious Diseases 32, no. 2 (2019): 176–186. 10.1097/QCO.0000000000000532 PMC6416007 30724789

[302] E. Takashita , “Influenza Polymerase Inhibitors: Mechanisms of Action and Resistance,” Cold Spring Harbor Perspectives in Medicine 11, no. 5 (2021): a038687. 10.1101/cshperspect.a038687 PMC8091960 32122918

[303] S. Coffey and P. A. Volberding , “Chapter 11 ‐ Overview of Antiretroviral Therapy,” in Sande's HIV/AIDS Medicine, ed. P. A. Volberding , W. C. Greene , J. M. A. Lange , J. E. Gallant , and N. Sewankambo (W.B. Saunders, 2012).

[304] A. Chandwani and J. Shuter , “Lopinavir/Ritonavir in the Treatment of HIV‐1 Infection: A Review,” Therapeutics and Clinical Risk Management 4, no. 5 (2008): 1023–1033. 10.2147/tcrm.s3285 PMC2621403 19209283

[305] P. de Leuw and C. Stephan , “Protease Inhibitors for the Treatment of Hepatitis C Virus Infection,” GMS Infectious Diseases 5 (2017): Doc08. 10.3205/id000034 PMC6301719 30671330

[306] F. Poordad , S. Pol , A. Asatryan , et al., “Glecaprevir/Pibrentasvir in Patients With Hepatitis C Virus Genotype 1 or 4 and Past Direct‐Acting Antiviral Treatment Failure,” Hepatology 67, no. 4 (2018): 1253–1260. 10.1002/hep.29671 PMC5901397 29152781

[307] A. Abutaleb , S. Kottilil , and E. Wilson , “Glecaprevir/Pibrentasvir Expands Reach While Reducing Cost and Duration of Hepatitis C Virus Therapy,” Hepatology International 12, no. 3 (2018): 214–222. 10.1007/s12072-018-9873-y PMC6436099 29845496

[308] H. P. Shin , “Sofosbuvir–Velpatasvir and Sofosbuvir–Velpatasvir–Voxilaprevir: Novel Treatment Options for Naïve and Previously Treated Hepatitis C Infection,” The Korean Journal of Internal Medicine 38, no. 4 (2023): 454–455. 10.3904/kjim.2023.248 PMC10338249 37424499

[309] D. Focosi , S. McConnell , S. Shoham , A. Casadevall , F. Maggi , and G. Antonelli , “Nirmatrelvir and Covid‐19: Development, Pharmacokinetics, Clinical Efficacy, Resistance, Relapse, and Pharmacoeconomics,” International Journal of Antimicrobial Agents 61, no. 2 (2023): 106708. 10.1016/j.ijantimicag.2022.106708 PMC9806926 36603694

[310] S. M. R. Hashemian , A. Sheida , M. Taghizadieh , et al., “Paxlovid (Nirmatrelvir/Ritonavir): A New Approach to COVID‐19 Therapy?,” Biomedicine & Pharmacotherapy 162 (2023): 114367. 10.1016/j.biopha.2023.114367 PMC9899776 37018987

[311] N. H. C. Loos , J. H. Beijnen , and A. H. Schinkel , “The Inhibitory and Inducing Effects of Ritonavir on Hepatic and Intestinal CYP3A and Other Drug‐Handling Proteins,” Biomedicine & Pharmacotherapy 162 (2023): 114636. 10.1016/j.biopha.2023.114636 PMC10065864 37004323

[312] X. Zhu , Y. Zhu , S. Ye , et al., “Improved Pharmacological and Structural Properties of HIV Fusion Inhibitor AP3 Over Enfuvirtide: Highlighting Advantages of Artificial Peptide Strategy,” Scientific Reports 5, no. 1 (2015): 13028. 10.1038/srep13028 PMC4541410 26286358

[313] M. R. Salvaggio and J. W. Gnann , “Chapter 153 ‐ Drugs for Herpesvirus Infections,” in Infectious Diseases, 4th ed, ed. J. Cohen , W. G. Powderly , and S. M. Opal (Elsevier, 2017).

[314] B. T. Mukuhlani and R. K. AssarÉ , “Efficacy and Safety of Long‐Acting Lenacapavir for HIV Treatment and Prevention: A Systematic Review of Randomized Trials,” International Journal of Infectious Diseases 167 (2026): 108580. 10.1016/j.ijid.2026.108580 41871735

[315] C. Zhang , B. Li , J. Li , H. Zhang , and Y. Wu , “New Advances in Anti‐HIV‐1 Strategies Targeting the Assembly and Stability of Capsid Protein,” International Journal of Molecular Sciences 26, no. 12 (2025): 5819. 10.3390/ijms26125819 PMC12193103 40565280

[316] M. S. McKellar , “Lenacapavir: A First‐in‐Class Capsid Inhibitor for HIV Treatment and Prevention,” Current Opinion in Infectious Diseases 38, no. 3 (2025): 208–213. 10.1097/QCO.0000000000001113 40276818

[317] A. Picardi , U. V. Gentilucci , E. M. Zardi , D. D'Avola , A. Amoroso , and A. Afeltra , “The Role of Ribavirin in the Combination Therapy of Hepatitis C Virus Infection,” Current Pharmaceutical Design 10, no. 17 (2004): 2081–2092. 10.2174/1381612043384330 15279547

[318] S. Beaucourt and M. Vignuzzi , “Ribavirin: A Drug Active Against Many Viruses With Multiple Effects on Virus Replication and Propagation. Molecular Basis of Ribavirin Resistance,” Current Opinion in Virology 8 (2014): 10–15. 10.1016/j.coviro.2014.04.011 PMC7102760 24846716

[319] Y. Furuta , T. Komeno , and T. Nakamura , “Favipiravir (T‐705), a Broad Spectrum Inhibitor of Viral RNA Polymerase,” Proceedings of the Japan Academy, Series B 93, no. 7 (2017): 449–463. 10.2183/pjab.93.027 PMC5713175 28769016

[320] M. A. Hernán and J. Del Amo , “Drug Repurposing and Observational Studies: The Case of Antivirals for the Treatment of COVID‐19,” Annals of Internal Medicine 176, no. 4 (2023): 556–560. 10.7326/M22-3582 PMC10064276 36972545

[321] J. F. Rossignol , “Nitazoxanide: A First‐in‐Class Broad‐Spectrum Antiviral Agent,” Antiviral Research 110 (2014): 94–103. 10.1016/j.antiviral.2014.07.014 PMC7113776 25108173

[322] S. Chaput , J.‐S. Driouich , B. Pastorino , et al., “Preclinical Efficacy of a Favipiravir and Nitazoxanide Combination Against Rift Valley Fever Virus,” Antiviral Research 249 (2026): 106402. 10.1016/j.antiviral.2026.106402 41936880

[323] J. F. Rossignol , “Nitazoxanide, a New Drug Candidate for the Treatment of Middle East Respiratory Syndrome Coronavirus,” Journal of Infection and Public Health 9, no. 3 (2016): 227–230. 10.1016/j.jiph.2016.04.001 PMC7102735 27095301

[324] S. Piacentini , S. La Frazia , A. Riccio , et al., “Nitazoxanide Inhibits Paramyxovirus Replication by Targeting the Fusion Protein Folding: Role of Glycoprotein‐Specific Thiol Oxidoreductase Erp57,” Scientific Reports 8, no. 1 (2018): 10425. 10.1038/s41598-018-28172-9 PMC6041319 29992955

[325] J. Haffizulla , A. Hartman , M. Hoppers , et al., “Effect of Nitazoxanide in Adults and Adolescents With Acute Uncomplicated Influenza: A Double‐Blind, Randomised, Placebo‐Controlled, Phase 2b/3 Trial,” The Lancet Infectious Diseases 14, no. 7 (2014): 609–618. 10.1016/S1473-3099(14)70717-0 PMC7164783 24852376

[326] M. Abuelazm , A. Ghanem , A. K. Awad , et al., “The Effect of Nitazoxanide on the Clinical Outcomes in Patients With COVID‐19: A Systematic Review and Meta‐Analysis of Randomized Controlled Trials,” Clinical Drug Investigation 42, no. 12 (2022): 1031–1047. 10.1007/s40261-022-01213-y PMC9628625 36315350

[327] J. Han , M. K. Lee , Y. Jang , W. J. Cho , and M. Kim , “Repurposing of Cyclophilin a Inhibitors as Broad‐Spectrum Antiviral Agents,” Drug Discovery Today 27, no. 7 (2022): 1895–1912. 10.1016/j.drudis.2022.05.016 PMC9123807 35609743

[328] I. F. Sevrioukova , “Interaction of Cyp3a4 With the Inhibitor Cobicistat: Structural and Mechanistic Insights and Comparison With Ritonavir,” Archives of Biochemistry and Biophysics 758 (2024): 110071. 10.1016/j.abb.2024.110071 PMC11286144 38909836

[329] J. Gerhart , D. S. Cox , R. S. P. Singh , et al., “A Comprehensive Review of the Clinical Pharmacokinetics, Pharmacodynamics, and Drug Interactions of Nirmatrelvir/Ritonavir,” Clinical Pharmacokinetics 63, no. 1 (2024): 27–42. 10.1007/s40262-023-01339-y PMC10786959 38177893

[330] Y. Libiad , B. Franck , C. Tron , et al., “Managing High‐Risk Drug Interactions With Nirmatrelvir/Ritonavir: Experience From a Dedicated Pharmacology Advice Service,” Clinical and Translational Science 19, no. 3 (2026): e70443. 10.1111/cts.70443 PMC12953180 41772767

[331] S. Abraham , A. Nohria , T. G. Neilan , et al., “Cardiovascular Drug Interactions With Nirmatrelvir/Ritonavir in Patients with COVID‐19,” Journal of the American College of Cardiology 80, no. 20 (2022): 1912–1924. 10.1016/j.jacc.2022.08.800 PMC9580069 36243540

[332] S. Liabeuf , J. Berdougo‐Tritz , L. Augey , et al., “Drug Exposure in Chronic Kidney Disease: It Is Not Just About the Glomerular Filtration Rate,” Fundamental & Clinical Pharmacology 39, no. 4 (2025): 1–10. 10.1111/fcp.70037 PMC12274659 40682291

[333] E. J. Smolders , C. T. de Kanter , B. van Hoek , J. E. Arends , J. P. Drenth , and D. M. Burger , “Pharmacokinetics, Efficacy, and Safety of Hepatitis C Virus Drugs in Patients With Liver and/or Renal Impairment,” Drug Safety 39, no. 7 (2016): 589–611. 10.1007/s40264-016-0420-2 PMC4912979 27098247

[334] K. Chami , K. K. Khaleq , M. El Goubi , M. Moussaoui , and K. El Aidaoui , “Acyclovir‐Induced Nephrotoxicity: A Case Report,” Cureus 17, no. 8 (2025): e89597. 10.7759/cureus.89597 PMC12414248 40922834

[335] A. P. W. Lim , J. Sung , V. Ramburuth , and S. O. Oyibo , “Acyclovir‐Induced Nephrotoxicity and Neurotoxicity: A Report of Two Cases,” Cureus 16, no. 1 (2024): e52367. 10.7759/cureus.52367 PMC10792401 38234392

[336] N. C. Neverette , J. Winfield , N. Chaparro‐Cáceres , K. S. Ho , J. B. Dumond , and A. S. Devanathan , “Contemporary Antiretroviral Pharmacology in the Male Genital Tract: Implications for HIV Treatment and Prevention,” Antimicrobial Agents and Chemotherapy 70, no. 5 (2026): e0140125. 10.1128/aac.01401-25 PMC13117926 41870063

[337] L. R. Gray , M. Roche , J. K. Flynn , S. L. Wesselingh , P. R. Gorry , and M. J. Churchill , “Is the Central Nervous System a Reservoir of HIV‐1?,” Current Opinion in HIV and AIDS 9, no. 6 (2014): 552–558. 10.1097/COH.0000000000000108 PMC4215931 25203642

[338] T. J. Cory , T. W. Schacker , M. Stevenson , and C. V. Fletcher , “Overcoming Pharmacologic Sanctuaries,” Current Opinion in HIV and AIDS 8, no. 3 (2013): 190–195. 10.1097/COH.0b013e32835fc68a PMC3677586 23454865

[339] S. Baldelli , F. Borgonovo , A. Foppoli , A. Gori , D. Cattaneo , and M. Cerea , “The Potential Role of Therapeutic Drug Monitoring for Safe and Effective Anti‐Infective Therapy With Manipulated Dosage Forms,” Pharmaceutics 18, no. 2 (2026): 176. 10.3390/pharmaceutics18020176 PMC12943953 41754918

[340] M. Nooruzzaman , K. E. E. Johnson , R. Rani , et al., “Emergence of Transmissible SARS‐CoV‐2 Variants With Decreased Sensitivity to Antivirals in Immunocompromised Patients With Persistent Infections,” Nature Communications 15, no. 1 (2024): 7999. 10.1038/s41467-024-51924-3 PMC11411086 39294134

[341] T. Ichikawa , T. Tamura , N. Nao , et al., “Characterization of Remdesivir Resistance Mutations in COVID‐19 Patients With Various Immunosuppressive Diseases,” Antiviral Research 242 (2025): 106264. 10.1016/j.antiviral.2025.106264 40885288

[342] S. Akinbolade , D. Coughlan , R. Fairbairn , et al., “Combination Therapies for COVID‐19: An Overview of the Clinical Trials Landscape,” British Journal of Clinical Pharmacology 88, no. 4 (2022): 1590–1597. 10.1111/bcp.15089 PMC8652622 34558094

[343] Z. A. Shyr , Y. S. Cheng , D. C. Lo , and W. Zheng , “Drug Combination Therapy for Emerging Viral Diseases,” Drug Discovery Today 26, no. 10 (2021): 2367–2376. 10.1016/j.drudis.2021.05.008 PMC8139175 34023496

[344] A. G. Nelson , X. Zhang , U. Ganapathi , et al., “Drug Delivery Strategies and Systems for HIV/AIDS Pre‐Exposure Prophylaxis and Treatment,” Journal of Controlled Release 219 (2015): 669–680. 10.1016/j.jconrel.2015.08.042 PMC4879940 26315816

[345] O. Falade‐Nwulia and M. S. Sulkowski , “Hepatitis C Virus Treatment: Simplifying the Simple and Optimizing the Difficult,” The Journal of Infectious Diseases 222, no. Suppl 9 (2020): S745–S757. 10.1093/infdis/jiaa534 PMC8171802 33245350

[346] S. Kim , J. Chen , T. Cheng , et al., “PubChem 2025 Update,” Nucleic Acids Research 53, no. D1 (2025): D1516–D1525. 10.1093/nar/gkae1059 PMC11701573 39558165

[347] S. Pleschka , “Special Issue “Host Targeted Therapeutics Against Virus Infections”,” Viruses 16, no. 12 (2024): 1825. 10.3390/v16121825 PMC11680107 39772137

[348] D. Zhou , Q. Mei , J. Li , and H. He , “Cyclophilin a and Viral Infections,” Biochemical and Biophysical Research Communications 424, no. 4 (2012): 647–650. 10.1016/j.bbrc.2012.07.024 PMC7092870 22814107

[349] P. Nigro , G. Pompilio , and M. C. Capogrossi , “Cyclophilin A: A Key Player for Human Disease,” Cell Death & Disease 4, no. 10 (2013): e888–e888. 10.1038/cddis.2013.410 PMC3920964 24176846

[350] P. A. Gallay and K. Lin , “Profile of Alisporivir and Its Potential in the Treatment of Hepatitis C,” Drug Design, Development and Therapy 7 (2013): 105. 10.2147/DDDT.S30946 PMC3578503 23440335

[351] S. Hopkins , M. Bobardt , U. Chatterji , J. A. Garcia‐Rivera , P. Lim , and P. A. Gallay , “The Cyclophilin Inhibitor Scy‐635 Disrupts Hepatitis C Virus Ns5a‐Cyclophilin a Complexes,” Antimicrobial Agents and Chemotherapy 56, no. 7 (2012): 3888–3897. 10.1128/AAC.00693-12 PMC3393457 22585215

[352] L. Coelmont , X. Hanoulle , U. Chatterji , et al., “DEB025 (Alisporivir) Inhibits Hepatitis C Virus Replication by Preventing a Cyclophilin a Induced Cis‐Trans Isomerisation in Domain II of NS5A,” PLoS ONE 5, no. 10 (2010): e13687. 10.1371/journal.pone.0013687 PMC2965138 21060866

[353] J. G. Low , C. Sung , L. Wijaya , et al., “Efficacy and Safety of Celgosivir in Patients With Dengue Fever (Celaden): A Phase 1b, Randomised, Double‐Blind, Placebo‐Controlled, Proof‐of‐Concept Trial,” The Lancet Infectious Diseases 14, no. 8 (2014): 706–715. 10.1016/S1473-3099(14)70730-3 24877997

[354] A. Lubkowska , W. Pluta , A. Strońska , and A. Lalko , “Role of Heat Shock Proteins (Hsp70 and Hsp90) in Viral Infection,” International Journal of Molecular Sciences 22, no. 17 (2021): 9366. 10.3390/ijms22179366 PMC8430838 34502274

[355] S. Nam , Y. J. Ga , J.‐Y. Lee , et al., “Radicicol Inhibits Chikungunya Virus Replication by Targeting Nonstructural Protein 2,” Antimicrobial Agents and Chemotherapy 65, no. 7 (2021): e0013521. 10.1128/AAC.00135-21 PMC8373223 33903104

[356] E. Weisberg , A. Parent , P. L. Yang , et al., “Repurposing of Kinase Inhibitors for Treatment of COVID‐19,” Pharmaceutical Research 37, no. 9 (2020): 167. 10.1007/s11095-020-02851-7 PMC7417114 32778962

[357] M. H. Raymonda , J. H. Ciesla , M. Monaghan , et al., “Pharmacologic Profiling Reveals Lapatinib as a Novel Antiviral Against SARS‐CoV‐2 in Vitro,” Virology 566 (2022): 60–68. 10.1016/j.virol.2021.11.008 PMC8626825 34871905

[358] J. C. Johnson , O. Martinez , A. N. Honko , L. E. Hensley , G. G. Olinger , and C. F. Basler , “Pyridinyl Imidazole Inhibitors of P38 Map Kinase Impair Viral Entry and Reduce Cytokine Induction by Zaire Ebolavirus in Human Dendritic Cells,” Antiviral Research 107 (2014): 102–109. 10.1016/j.antiviral.2014.04.014 PMC4103912 24815087

[359] A. Nagila , J. Netsawang , A. Suttitheptumrong , et al., “Inhibition of p38MAPK and CD137 Signaling Reduce Dengue Virus‐Induced TNF‐Α Secretion and Apoptosis,” Virology Journal 10 (2013): 105. 10.1186/1743-422X-10-105 PMC3639879 23557259

[360] L. Zona , J. Lupberger , N. Sidahmed‐Adrar , et al., “HRAS Signal Transduction Promotes Hepatitis C Virus Cell Entry by Triggering Assembly of the Host Tetraspanin Receptor Complex,” Cell Host & Microbe 13, no. 3 (2013): 302–313. 10.1016/j.chom.2013.02.006 23498955

[361] S. Stindt , P. Cebula , U. Albrecht , et al., “Hepatitis C Virus Activates a Neuregulin‐Driven Circuit to Modify Surface Expression of Growth Factor Receptors of the ERBB Family,” PLoS ONE 11, no. 2 (2016): e0148711. 10.1371/journal.pone.0148711 PMC4757098 26886748

[362] F.‐C. Lin and H. A. Young , “Interferons: Success in Anti‐Viral Immunotherapy,” Cytokine & Growth Factor Reviews 25, no. 4 (2014): 369–376. 10.1016/j.cytogfr.2014.07.015 PMC4182113 25156421

[363] M. Enomoto , A. Tamori , Y. Murakami , and N. Kawada , “Interferon‐α/β for Treatment of Chronic Hepatitis C Infection in the Era of Direct‐Acting Antiviral Agents,” Hepatology Research 44, no. 4 (2014): 371–376. 10.1111/hepr.12289 24330005

[364] G. Reis , E. A. S. Moreira Silva , D. C. Medeiros Silva , et al., “Early Treatment With Pegylated Interferon Lambda for COVID‐19,” New England Journal of Medicine 388, no. 6 (2023): 518–528. 10.1056/NEJMoa2209760 PMC9933926 36780676

[365] S. Blake , T. P. Hughes , G. Mayrhofer , and A. B. Lyons , “The Src/Abl Kinase Inhibitor Dasatinib (BMS‐354825) Inhibits Function of Normal Human T‐Lymphocytes in Vitro,” Clinical Immunology 127, no. 3 (2008): 330–339. 10.1016/j.clim.2008.02.006 18395492

[366] V. C. Yan and F. L. Muller , “Why Remdesivir Failed: Preclinical Assumptions Overestimate the Clinical Efficacy of Remdesivir for COVID‐19 and Ebola,” Antimicrobial Agents and Chemotherapy 65, no. 10 (2021): e0111721. 10.1128/AAC.01117-21 PMC8448091 34252308

[367] M. Strzelec , J. Detka , P. Mieszczak , M. K. Sobocińska , and M. Majka , “Immunomodulation—A General Review of the Current State‐of‐the‐Art and New Therapeutic Strategies for Targeting the Immune System,” Frontiers in Immunology 14 (2023): 1127704. 10.3389/fimmu.2023.1127704 PMC10033545 36969193

[368] C. Remmington , N. A. Barrett , S. Agarwal , et al., “Steroid Exposure and Outcome in COVID‐19 Pneumonia,” BJA Open 5 (2023): 100128. 10.1016/j.bjao.2023.100128 PMC9886648 36744291

[369] A. Perner and B. Venkatesh , “Higher‐Dose Dexamethasone for Patients With COVID‐19 and Hypoxaemia?,” The Lancet 401, no. 10387 (2023): 1474–1476. 10.1016/S0140-6736(23)00587-1 PMC10097504 37060914

[370] P. Horby , W. S. Lim , et al., “Dexamethasone in Hospitalized Patients With COVID‐19,” New England Journal of Medicine 384, no. 8 (2021): 693–704. 10.1056/NEJMoa2021436 PMC7383595 32678530

[371] F. Angriman , B. L. Ferreyro , L. Burry , et al., “Interleukin‐6 Receptor Blockade in Patients With COVID‐19: Placing Clinical Trials Into Context,” The Lancet Respiratory Medicine 9, no. 6 (2021): 655–664. 10.1016/S2213-2600(21)00139-9 PMC8078877 33930329

[372] B. Ruiz‐Antorán , A. Sancho‐López , F. Torres , et al., “Combination of Tocilizumab and Steroids to Improve Mortality in Patients With Severe COVID‐19 Infection: A Spanish, Multicenter, Cohort Study,” Infectious Diseases and Therapy 10, no. 1 (2021): 347–362. 10.1007/s40121-020-00373-8 PMC7719057 33280066

[373] T. Huet , H. Beaussier , O. Voisin , et al., “Anakinra for Severe Forms of COVID‐19: A Cohort Study,” The Lancet Rheumatology 2, no. 7 (2020): e393–e400. 10.1016/S2665-9913(20)30164-8 PMC7259909 32835245

[374] E. J. Kooistra , N. J. B. Waalders , M. Kox , and P. Pickkers , “Effect of Anakinra in COVID‐19,” The Lancet Rheumatology 2, no. 9 (2020): e523–e524. 10.1016/S2665-9913(20)30235-6 PMC7380916 32838316

[375] M. Franzetti , A. Forastieri , N. Borsa , et al., “Il‐1 Receptor Antagonist Anakinra in the Treatment of COVID‐19 Acute Respiratory Distress Syndrome: A Retrospective, Observational Study,” The Journal of Immunology 206, no. 7 (2021): 1569–1575. 10.4049/jimmunol.2001126 PMC7980530 33547169

[376] R. Nagasawa , T. Niwa , E. Hagiwara , et al., “Safety and Efficacy of Combination Therapy of Remdesivir, Baricitinib, and High‐Dose Steroids in Patients Hospitalized With Moderate to Severe COVID‐19,” Internal Medicine 62, no. 21 (2023): 3125–3130. 10.2169/internalmedicine.0761-22 PMC10686726 37438142

[377] A. C. Kalil , T. F. Patterson , A. K. Mehta , et al., “Baricitinib Plus Remdesivir for Hospitalized Adults With COVID‐19,” New England Journal of Medicine 384, no. 9 (2021): 795–807. 10.1056/NEJMoa2031994 PMC7745180 33306283

[378] M. Marin , A. Ruiz , D. Ouchi , et al., “PD‐1 Blockade Enhances Functional Vaccine‐Induced HIV‐1 CD8+ T‐Cell Responses in PWH Receiving Early ART,” EBioMedicine 123 (2026): 106070. 10.1016/j.ebiom.2025.106070 PMC12752766 41370970

[379] J. M. Benito , C. Restrepo , J. García‐Foncillas , and N. Rallón , “Immune Checkpoint Inhibitors as Potential Therapy for Reverting T‐Cell Exhaustion and Reverting HIV Latency in People Living With HIV,” Frontiers in Immunology 14 (2023): 1270881. 10.3389/fimmu.2023.1270881 PMC10733458 38130714

[380] V. Barili , A. Vecchi , M. Rossi , et al., “Unraveling the Multifaceted Nature of CD8 T Cell Exhaustion Provides the Molecular Basis for Therapeutic T Cell Reconstitution in Chronic Hepatitis B and C,” Cells 10, no. 10 (2021): 2563. 10.3390/cells10102563 PMC8533840 34685543

[381] M. Su , T. Ye , W. Wu , Z. Shu , and Q. Xia , “Possibility of PD‐1/PD‐L1 Inhibitors for the Treatment of Patients With Chronic Hepatitis B Infection,” Digestive Diseases 42, no. 1 (2024): 53–60. 10.1159/000534535 PMC10836741 37820605

[382] E. A. Burns , I. N. Muhsen , K. Anand , et al., “Hepatitis B Virus Reactivation in Cancer Patients Treated With Immune Checkpoint Inhibitors,” Journal of Immunotherapy (Hagerstown, Md: 1997) 44, no. 3 (2021): 132–139. 10.1097/CJI.0000000000000358 PMC7946380 33480637

[383] B. Li , C. Yan , J. Zhu , et al., “Anti‐PD‐1/PD‐L1 Blockade Immunotherapy Employed in Treating Hepatitis B Virus Infection‐Related Advanced Hepatocellular Carcinoma: A Literature Review,” Frontiers in Immunology 11 (2020): 1037. 10.3389/fimmu.2020.01037 PMC7270402 32547550

[384] E. Gane , D. J. Verdon , A. E. Brooks , et al., “Anti‐Pd‐1 Blockade With Nivolumab With and Without Therapeutic Vaccination for Virally Suppressed Chronic Hepatitis B: A Pilot Study,” Journal of Hepatology 71, no. 5 (2019): 900–907. 10.1016/j.jhep.2019.06.028 31306680

[385] R. A. Rawlings , V. Krishnan , and N. G. Walter , “Viral RNAi Suppressor Reversibly Binds siRNA to Outcompete Dicer and RISC via Multiple Turnover,” Journal of Molecular Biology 408, no. 2 (2011): 262–276. 10.1016/j.jmb.2011.02.038 PMC3073027 21354178

[386] R. Zhou and T. M. Rana , “RNA‐Based Mechanisms Regulating Host–Virus Interactions,” Immunological Reviews 253, no. 1 (2013): 97–111. 10.1111/imr.12053 PMC3695692 23550641

[387] J. Scharner and I. Aznarez , “Clinical Applications of Single‐Stranded Oligonucleotides: Current Landscape of Approved and in‐Development Therapeutics,” Molecular Therapy 29, no. 2 (2021): 540–554. 10.1016/j.ymthe.2020.12.022 PMC7854307 33359792

[388] S. Chen , B. H. Poudel , and R. N. Veedu , “An LNA Gapmer Antisense Oligonucleotide Inhibitor of Human Nnmt,” Molecular Therapy Nucleic Acids 36, no. 4 (2025): 102761. 10.1016/j.omtn.2025.102761 PMC12661993 41323798

[389] B. Hu , L. Zhong , Y. Weng , et al., “Therapeutic siRNA: State of the Art,” Signal Transduction and Targeted Therapy 5, no. 1 (2020): 101. 10.1038/s41392-020-0207-x PMC7305320 32561705

[390] S. Y. Wu , X. Yang , K. M. Gharpure , et al., “2′‐OMe‐Phosphorodithioate‐Modified siRNAs Show Increased Loading Into the RISC Complex and Enhanced Anti‐Tumour Activity,” Nature Communications 5, no. 1 (2014): 3459. 10.1038/ncomms4459 PMC4112501 24619206

[391] S. Yuan , A. Nisar , C. Chen , et al., “Liver‐Targeted Degradation of Brd4 Reverses Hepatic Fibrosis and Enhances Metabolism in Murine Models,” Theranostics 15, no. 15 (2025): 7270–7290. 10.7150/thno.113852 PMC12315693 40756370

[392] B. Roehr , “Fomivirsen Approved for CMV Retinitis,” Journal of the International Association of Physicians in AIDS Care 4, no. 10 (1998): 14–16. 11365956

[393] M.‐F. Yuen , S.‐G. Lim , R. Plesniak , et al., “Efficacy and Safety of Bepirovirsen in Chronic Hepatitis B Infection,” New England Journal of Medicine 387, no. 21 (2022): 1957–1968. 10.1056/NEJMoa2210027 36346079

[394] J. Cremer , R. Elston , F. M. Campbell , et al., “B‐Clear Phase 2b Study Design: Establishing the Efficacy and Safety of Bepirovirsen in Patients With Chronic Hepatitis B Virus Infection,” Advances in Therapy 40, no. 9 (2023): 4101–4110. 10.1007/s12325-023-02531-z PMC10427703 37393402

[395] J. DeVincenzo , R. Lambkin‐Williams , T. Wilkinson , et al., “A Randomized, Double‐Blind, Placebo‐Controlled Study of an RNAi‐Based Therapy Directed Against Respiratory Syncytial Virus,” Proceedings of the National Academy of Sciences of the United States of America 107, no. 19 (2010): 8800–8805. 10.1073/pnas.0912186107 PMC2889365 20421463

[396] Y.‐C. Chang , C.‐F. Yang , Y.‐F. Chen , et al., “A siRNA Targets and Inhibits a Broad Range of SARS‐CoV‐2 Infections Including Delta Variant,” EMBO Molecular Medicine 14, no. 4 (2022): e15298. 10.15252/emmm.202115298 PMC8988202 35138028

[397] H. L. A. Janssen , H. W. Reesink , E. J. Lawitz , et al., “Treatment of HCV Infection by Targeting Microrna,” New England Journal of Medicine 368, no. 18 (2013): 1685–1694. 10.1056/NEJMoa1209026 23534542

[398] S. Ottosen , T. B. Parsley , L. Yang , et al., “In Vitro Antiviral Activity and Preclinical and Clinical Resistance Profile of Miravirsen, a Novel Anti‐Hepatitis C Virus Therapeutic Targeting the Human Factor miR‐122,” Antimicrobial Agents and Chemotherapy 59, no. 1 (2015): 599–608. 10.1128/AAC.04220-14 PMC4291405 25385103

[399] P. K. Dash , C. Chen , R. Kaminski , et al., “CRISPR Editing of Ccr5 and HIV‐1 Facilitates Viral Elimination in Antiretroviral Drug‐Suppressed Virus‐Infected Humanized Mice,” Proceedings of the National Academy of Sciences of the United States of America 120, no. 19 (2023): e2217887120. 10.1073/pnas.2217887120 PMC10175831 37126704

[400] T. R. Abbott , G. Dhamdhere , Y. Liu , et al., “Development of Crispr as an Antiviral Strategy to Combat SARS‐CoV‐2 and Influenza,” Cell 181, no. 4 (2020): 865–876.e12. 10.1016/j.cell.2020.04.020 PMC7189862 32353252

[401] X. Tan , J. Li , B. Cui , et al., “Crispr/Cas13‐Based Anti‐RNA Viral Approaches,” Genes 16, no. 8 (2025): 875. 10.3390/genes16080875 PMC12385312 40869923

[402] J. F. Bot , Z. Zhao , M. Li , D. Kammeron , P. Shang , and N. Geijsen , “Temporal Dynamics of Collateral RNA Cleavage by LbuCas13a in Human Cells,” Communications Biology 9, no. 1 (2026): 233. 10.1038/s42003-026-09511-3 PMC12901986 41554880

[403] J. F. Bot , J. van der Oost , and N. Geijsen , “The Double Life of CRISPR–Cas13,” Current Opinion in Biotechnology 78 (2022): 102789. 10.1016/j.copbio.2022.102789 36115160

[404] G. Serpa , Q. Gong , M. De , et al., “Detection of Pre‐Existing Immunity to Bacterial Cas9 Proteins in People With Cystic Fibrosis,” ImmunoHorizons 9, no. 10 (2025): vlaf041. 10.1093/immhor/vlaf041 PMC12448903 40972653

[405] N. Djassemi , B. Hanisch , C. Motta , et al., “Harnessing Virus‐Specific T Cells: Expanding Therapeutic Strategies Across Diverse Populations,” Blood Advances 9, no. 23 (2025): 5965–5975. 10.1182/bloodadvances.2024013727 PMC12686789 40864193

[406] C. M. Bollard and H. E. Heslop , “T Cells for Viral Infections After Allogeneic Hematopoietic Stem Cell Transplant,” Blood 127, no. 26 (2016): 3331–3340. 10.1182/blood-2016-01-628982 PMC4929925 27207801

[407] N. Su , Z. Liu , P. Sun , F. Gu , X. Yan , and D. Cai , “Donor‐Derived Cytomegalovirus‐Cytotoxic T Lymphocytes and Leflunomide Successfully Control Refractory Cytomegalovirus Infections and Disease of Multiple Sites After Allogeneic‐Hematopoietic Stem Cell Transplantation: A Case Report,” Frontiers in Medicine 9 (2022): 948210. 10.3389/fmed.2022.948210 PMC9485495 36148446

[408] M. Barkhordar , M. Samareh Salavatipour , S. Tavakoli , et al., “Prophylactic Infusion of Donor Derived Cmv Specific T Cells for the Prevention of CMV Reactivation Following Allogeneic HSCT,” Scientific Reports 15, no. 1 (2025): 43163. 10.1038/s41598-025-27354-6 PMC12678814 41345418

[409] C. M. Bollard , C. M. Rooney , and H. E. Heslop , “T‐Cell Therapy in the Treatment of Post‐Transplant Lymphoproliferative Disease,” Nature Reviews Clinical Oncology 9, no. 9 (2012): 510–519. 10.1038/nrclinonc.2012.111 PMC3743122 22801669

[410] M. Quinn , D. A. Erkes , and C. M. Snyder , “Cytomegalovirus and Immunotherapy: Opportunistic Pathogen, Novel Target for Cancer and a Promising Vaccine Vector,” Immunotherapy 8, no. 2 (2016): 211–221. 10.2217/imt.15.110 PMC5619022 26786895

[411] K. A. Baugh , I. Tzannou , and A. M. Leen , “Infusion of Cytotoxic T Lymphocytes for the Treatment of Viral Infections in Hematopoetic Stem Cell Transplant Patients,” Current Opinion in Infectious Diseases 31, no. 4 (2018): 292–300. 10.1097/QCO.0000000000000456 PMC7896200 29750672

[412] A. Papadopoulou , U. Gerdemann , U. L. Katari , et al., “Activity of Broad‐Spectrum T Cells as Treatment for ADV, EBV, CMV, BKV, and HHV6 Infections After HSCT,” Science Translational Medicine 6, no. 242 (2014): 242ra283. 10.1126/scitranslmed.3008825 PMC4181611 24964991

[413] Z. A. Hannouneh , M. Merzkani , C.‐S. Hsieh , and N. Murakami , “Cellular Therapies in Transplantation—Regulatory T Cell Therapies and Virus Specific Therapies,” Current Transplantation Reports 12, no. 1 (2025): 33. 10.1007/s40472-025-00489-1 PMC12499345 41058937

[414] S. Davies , E. Blyth , C. M. Bollard , et al., “Third‐Party Viral Specific T Cells for Immunocompromised Hosts and Access Barriers: A Review and Commentary by an Expert Panel From ASTCT,” Transplantation and Cellular Therapy 32 (2026): 1051.e1–1051.e10. 10.1016/j.jtct.2026.04.027 42081980

[415] J. D. Rubinstein , G. Pham , A. Sridharan , et al., “Outcomes With Third‐Party Virus‐Specific T Cells After the Use of Single‐Antigen Cell Lines to Predict HLA Restriction,” Blood Advances 9, no. 24 (2025): 6305–6313. 10.1182/bloodadvances.2025017097 PMC12744286 40991376

[416] M. D. Padurariu‐Covit , C. Georgescu , M. Andreescu , I. Chiscop , C. Plesea‐Condratovici , and M. Arbune , “Car‐T Cell Therapy for HIV Cure: Current Challenges, Advances and Future Directions,” Viruses 17, no. 12 (2025): 1615. 10.3390/v17121615 PMC12737754 41472284

[417] A. S. Kuhlmann , C. W. Peterson , and H. P. Kiem , “Chimeric Antigen Receptor T‐Cell Approaches to HIV Cure,” Current Opinion in HIV and AIDS 13, no. 5 (2018): 446–453. 10.1097/COH.0000000000000485 PMC6993924 29878913

[418] Y. Mao , Q. Liao , Y. Zhu , et al., “Efficacy and Safety of Novel Multifunctional M10 CAR‐T Cells in HIV‐1‐Infected Patients: A Phase I, Multicenter, Single‐Arm, Open‐Label Study,” Cell Discovery 10, no. 1 (2024): 49. 10.1038/s41421-024-00658-z PMC11091177 38740803

[419] F. H. Rothemejer , N. P. Lauritsen , A. K. Juhl , et al., “Development of HIV‐Resistant CAR T Cells by CRISPR/Cas‐Mediated CAR Integration Into the CCR5 Locus,” Viruses 15, no. 1 (2023): 202. 10.3390/v15010202 PMC9862650 36680242

[420] W. Mu , M. A. Carrillo , and S. G. Kitchen , “Engineering Car T Cells to Target the HIV Reservoir,” Frontiers in Cellular and Infection Microbiology 10 (2020): 410. 10.3389/fcimb.2020.00410 PMC7438537 32903563

[421] F. Wang , J. Li , Y. Huang , et al., “CAR‐T Cell Engineered With TCR‐Like Antibody Specific for HBV Surface Antigen Epitope E183–91/HLA‐A *0201 Exhibit Potent Activity Against HBV‐HCC,” Oncoimmunology 14, no. 1 (2025): 2546404. 10.1080/2162402X.2025.2546404 PMC12363532 40820888

[422] A. Bertoletti and A. T. Tan , “Engineering HBV‐Specific T Cells for the Treatment of HBV‐Related HCC and HBV Infection: Past, Present, and Future. Editorial on ”Genetically‐Modified, Redirected T Cells Target Hepatitis B Surface Antigen‐Positive Hepatocytes and Hepatocellular Carcinoma Lesions in a Clinical Setting“,” Clinical and Molecular Hepatology 30, no. 4 (2024): 728–734. 10.3350/cmh.2024.0469 PMC11540402 38934109

[423] S. J. Lu and Q. Feng , “CAR‐NK Cells From Engineered Pluripotent Stem Cells: Off‐the‐Shelf Therapeutics for all Patients,” Stem Cells Translational Medicine 10 Suppl 2, no. Suppl 2 (2021): S10–S17. 10.1002/sctm.21-0135 PMC8560199 34724715

[424] S. Balkhi , G. Zuccolotto , A. Di Spirito , A. Rosato , and L. Mortara , “CAR‐NK Cell Therapy: Promise and Challenges in Solid Tumors,” Frontiers in Immunology 16 (2025): 1574742. 10.3389/fimmu.2025.1574742 PMC12009813 40260240

[425] UNAIDS , “Global HIV & AIDS Statistics — Fact Sheet. Updated on 10 July 2025,” (2025), https://www.unaids.org/en/resources/fact–sheet.

[426] R. J. Landovitz , B. S. Hanscom , M. E. Clement , et al., “Efficacy and Safety of Long‐Acting Cabotegravir Compared With Daily Oral Tenofovir Disoproxil Fumarate Plus Emtricitabine to Prevent HIV Infection in Cisgender Men and Transgender Women Who Have Sex With Men 1 Year After Study Unblinding: A Secondary Analysis of the Phase 2b and 3 HPTN 083 Randomised Controlled Trial,” Lancet HIV 10, no. 12 (2023): e767–e778. 10.1016/S2352-3018(23)00261-8 PMC11375758 37952550

[427] X. Chen , J. Li , L. Kou , X. Xie , D. Wei , and Y. Li , “Efficacy and Safety of Long‐Acting Cabotegravir Versus Oral Tenofovir Disoproxil Fumarate‐Emtricitabine as HIV Pre‐Exposure Prophylaxis: A Systematic Review and Meta‐Analysis,” Reviews in Medical Virology 33, no. 4 (2023): e2460. 10.1002/rmv.2460 37198721

[428] M. A. Spinelli , B. Grinsztejn , and R. J. Landovitz , “Promises and Challenges: Cabotegravir for Preexposure Prophylaxis,” Current Opinion in HIV and AIDS 17, no. 4 (2022): 186–191. 10.1097/COH.0000000000000733 PMC9240402 35762372

[429] M. M. Sajadi , A. Abbasi , Z. R. Tehrani , et al., “A Comprehensive Engineering Strategy Improves Potency and Manufacturability of a Near Pan‐Neutralizing Antibody Against HIV,” Structure 33, no. 7 (2025): 1150–1164.e8. 10.1016/j.str.2025.04.016 PMC12227296 40373766

[430] J. Tuan and O. Ogbuagu , “Lenacapavir: A Twice‐Yearly Treatment for Adults With Multidrug‐Resistant HIV Infection and Limited Treatment Options,” Expert Review of Anti‐Infective Therapy 21, no. 6 (2023): 565–570. 10.1080/14787210.2023.2203913 37067160

[431] A. Mullard , “Fda Approves Twice‐Yearly Capsid Inhibitor for HIV Prevention,” Nature Reviews Drug Discovery 24, no. 8 (2025): 577. 10.1038/d41573-025-00117-8 40629006

[432] J. E. Flores , A. J. Thompson , M. Ryan , and J. Howell , “The Global Impact of Hepatitis B Vaccination on Hepatocellular Carcinoma,” Vaccines 10, no. 5 (2022): 793. 10.3390/vaccines10050793 PMC9144632 35632549

[433] L. Romano' and A. R. Zanetti , “Hepatitis B Vaccination: A Historical Overview With a Focus on the Italian Achievements,” Viruses 14, no. 7 (2022): 1515. 10.3390/v14071515 PMC9320049 35891495

[434] L. C. Meireles , “Three Decades of Hepatitis B Control With Vaccination,” World Journal of Hepatology 7, no. 18 (2015): 2127. 10.4254/wjh.v7.i18.2127 PMC4550866 26328023

[435] A. S. F. Lok , “Toward a Functional Cure for Hepatitis B,” Gut and Liver 18, no. 4 (2024): 593–601. 10.5009/gnl240023 PMC11249939 38533651

[436] A. Kaewdech and P. Sripongpun , “Challenges in the Discontinuation of Chronic Hepatitis B Antiviral Agents,” World Journal of Hepatology 13, no. 9 (2021): 1042–1057. 10.4254/wjh.v13.i9.1042 PMC8473499 34630873

[437] J. Yang , J.‐L. Qi , X.‐X. Wang , et al., “The Burden of Hepatitis C Virus in the World, China, India, and the United States From 1990 to 2019,” Frontiers in Public Health 11 (2023): 1041201. 10.3389/fpubh.2023.1041201 PMC10018168 36935711

[438] Ontario‐Health , “Hepatitis C Screening: A Health Technology Assessment,” Ontario Health Technology Assessment Series 26, no. 1 (2026):1–139. PMC12929945 41743076

[439] H. K. Bhatia , H. Singh , N. Grewal , and N. K. Natt , “Sofosbuvir: A Novel Treatment Option for Chronic Hepatitis C Infection,” Journal of Pharmacology and Pharmacotherapeutics 5, no. 4 (2014): 278–284. 10.4103/0976-500X.142464 PMC4231565 25422576

[440] D. Gritsenko and G. Hughes , “Ledipasvir/Sofosbuvir (Harvoni): Improving Options for Hepatitis C Virus Infection,” P & T: A Peer‐Reviewed Journal for Formulary Management 40, no. 4 (2015): 256–276. PMC4378517 25859119

[441] A. L. Zignego , M. Monti , and L. Gragnani , “Sofosbuvir/Velpatasvir for the Treatment of Hepatitis C Virus Infection,” Acta Biomedica 89, no. 3 (2018): 321–331. 10.23750/abm.v89i3.7718 PMC6502110 30333452

[442] S. Pol , A. J. Thompson , M. Collins , et al., “Effectiveness and Safety of Glecaprevir/Pibrentasvir for 8 Weeks in the Treatment of Patients With Acute Hepatitis C: A Single‐Arm Retrospective Study,” Hepatology 81, no. 3 (2025): 1006–1018. 10.1097/HEP.0000000000000923 PMC11825497 38768260

[443] F. Cao , Y.‐Z. Li , D.‐Y. Zhang , et al., “Human Papillomavirus Infection and the Risk of Cancer at Specific Sites Other Than Anogenital Tract and Oropharyngeal Region: An Umbrella Review,” EBioMedicine 104 (2024): 105155. 10.1016/j.ebiom.2024.105155 PMC11108822 38744109

[444] A. Yusupov , D. Popovsky , L. Mahmood , A. S. Kim , A. E. Akman , and H. Yuan , “The Nonavalent Vaccine: A Review of High‐Risk HPVS and a Plea to the CDC,” Am J Stem Cells 8, no. 3 (2019): 52–64. PMC6971474 31976155

[445] P. L. Stern , S. H. van der Burg , I. N. Hampson , et al., “Therapy of Human Papillomavirus‐Related Disease,” Vaccine 30 Suppl 5, no. 0 5 (2012): F71–F82. 10.1016/j.vaccine.2012.05.091 PMC4155500 23199967

[446] Y. Kyokha Ameen , “Seasonal Influenza: A Narrative Review of Epidemiology, Clinical Features, and Preventive Strategies,” Cureus 17, no. 10 (2025): e95336. 10.7759/cureus.95336 PMC12640676 41287674

[447] S. D. Jazayeri and C. L. Poh , “Development of Universal Influenza Vaccines Targeting Conserved Viral Proteins,” Vaccines 7, no. 4 (2019): 169. 10.3390/vaccines7040169 PMC6963725 31683888

[448] J. Reina and N. Reina , “[Baloxavir Marboxil: A Potent Cap‐Dependent Endonuclease Inhibitor of Influenza Viruses],” Revista Espanola De Quimioterapia 32, no. 1 (2019): 1–5. PMC6372968 30676002

[449] M. Veldhoen and J. P. Simas , “Endemic SARS‐CoV‐2 Will Maintain Post‐Pandemic Immunity,” Nature Reviews Immunology 21, no. 3 (2021): 131–132. 10.1038/s41577-020-00493-9 PMC7784621 33402727

[450] N. Z. Zabidi , H. L. Liew , I. A. Farouk , et al., “Evolution of SARS‐CoV‐2 Variants: Implications on Immune Escape, Vaccination, Therapeutic and Diagnostic Strategies,” Viruses 15, no. 4 (2023): 944. 10.3390/v15040944 PMC10145020 37112923

[451] X.‐C. Xiong , N.‐N. Zhang , M. Wu , et al., “Development and Characterization of a Bivalent mRNA Vaccine Targeting the Delta and Omicron Variants of SARS‐CoV‐2,” Human Vaccines & Immunotherapeutics 21, no. 1 (2025): 2562730. 10.1080/21645515.2025.2562730 PMC12489999 41025542

[452] M. Azeem , P. Cancemi , F. Mukhtar , et al., “Efficacy and Limitations of SARS‐CoV‐2 Vaccines—A Systematic Review,” Life Sciences 371 (2025): 123610. 10.1016/j.lfs.2025.123610 40189198

[453] C. C. Butler , A. D. Pinto , V. Harris , et al., “Oral Nirmatrelvir–Ritonavir for COVID‐19 in Higher‐Risk Outpatients,” New England Journal of Medicine 394, no. 16 (2026): 1583–1594. 10.1056/NEJMoa2502457 PMC7619176 42019019

[454] T. Kino , I. Burd , and J. H. Segars , “Dexamethasone for Severe COVID‐19: How Does It Work at Cellular and Molecular Levels?,” International Journal of Molecular Sciences 22, no. 13 (2021): 6764. 10.3390/ijms22136764 PMC8269070 34201797

[455] R. J. Pickles and J. P. DeVincenzo , “Respiratory Syncytial Virus (RSV) and Its Propensity for Causing Bronchiolitis,” The Journal of Pathology 235, no. 2 (2015): 266–276. 10.1002/path.4462 PMC5638117 25302625

[456] T. S. Raghuveer and R. Zackula , “Nirsevimab for Prevention of RSV Hospitalizations in Infants,” The New England Journal of Medicine 390, no. 12 (2024): 1152. 10.1056/NEJMc2400983 38507765

[457] J. Torres‐Torres , L. Rojas‐Zepeda , J. R. Villafan‐Bernal , et al., “Maternal RSV Vaccination for Infant Protection: A Systematic Review and Meta‐Analysis of Phase 3 Trials With an Integrated Economic Evaluation,” International Journal of Gynecology & Obstetrics 173, no. 1 (2026): 63–73. 10.1002/ijgo.70641 PMC12988398 41189343

[458] M. C. H. Lassen , N. D. Johansen , S. H. Christensen , et al., “RSV Prefusion F Vaccine for Prevention of Hospitalization in Older Adults,” New England Journal of Medicine 394, no. 2 (2026): 138–151. 10.1056/NEJMoa2509810 40888695

[459] J. B. Domachowske , E. J. Anderson , and M. Goldstein , “The Future of Respiratory Syncytial Virus Disease Prevention and Treatment,” Infectious Diseases and Therapy 10, no. Suppl 1 (2021): 47–60. 10.1007/s40121-020-00383-6 PMC7926075 33656652

[460] S. T. Jacob , I. Crozier , W. A. Fischer , et al., “Ebola Virus Disease,” Nature Reviews Disease Primers 6, no. 1 (2020): 13. 10.1038/s41572-020-0147-3 PMC7223853 32080199

[461] C. M. Kahusu , L. Peckeu‐Abboud , O. Goovaerts , et al., “Long‐Term Antibody Responses to the Ebola Virus and the Vaccine Vector After RVSV‐ZEBOV Vaccination in Drc,” Vaccine 62 (2025): 127537. 10.1016/j.vaccine.2025.127537 40706506

[462] A. N. Pinski and I. Messaoudi , “Therapeutic Vaccination Strategies Against EBOV by rVSV‐EBOV‐GP: The Role of Innate Immunity,” Current Opinion in Virology 51 (2021): 179–189. 10.1016/j.coviro.2021.10.007 PMC8884032 34749265

[463] C. Woolsey and T. W. Geisbert , “Current State of Ebola Virus Vaccines: A Snapshot,” PLOS Pathogens 17, no. 12 (2021): e1010078. 10.1371/journal.ppat.1010078 PMC8659338 34882741

[464] A. N. Prasad , C. Woolsey , V. Borisevich , et al., “Remdesivir, mAb114, REGN‐EB3, and ZMapp Partially Rescue Nonhuman Primates Infected With a Low Passage Kikwit Variant of Ebola Virus,” Nature Communications 16, no. 1 (2025): 3824. 10.1038/s41467-025-59168-5 PMC12019533 40268932

[465] E. S. Paixão , M. G. Teixeira , and L. C. Rodrigues , “Zika, Chikungunya and Dengue: The Causes and Threats of New and Re‐Emerging Arboviral Diseases,” BMJ Global Health 3, no. Suppl 1 (2018): e000530. 10.1136/bmjgh-2017-000530 PMC5759716 29435366

[466] R. Kant , A. Singh , A. B. Ali , S. Nagre , N. K. Ganguly , and R. Rana , “Dengue Vaccine Effectiveness and Safety: A Systematic Analysis of Recent Clinical Trial Data,” Virology Journal 22, no. 1 (2025): 384. 10.1186/s12985-025-02937-x PMC12641897 41287091

[467] X. Sáez‐Llorens , R. DeAntonio , J. G. H. Low , et al., “Tak‐003: Development of a Tetravalent Dengue Vaccine,” Expert Review of Vaccines 24, no. 1 (2025): 324–338. 10.1080/14760584.2025.2490295 40207772

[468] Y. Wang , L. Ling , Z. Zhang , and A. Marin‐Lopez , “Current Advances in Zika Vaccine Development,” Vaccines 10, no. 11 (2022): 1816. 10.3390/vaccines10111816 PMC9694033 36366325

[469] L. H. Chen , A. Fritzer , R. Hochreiter , K. Dubischar , and S. Meyer , “From Bench to Clinic: The Development of Vla1553/Ixchiq, a Live‐Attenuated Chikungunya Vaccine,” Journal of Travel Medicine 31, no. 7 (2024): taae123. 10.1093/jtm/taae123 PMC11497415 39255380

[470] S. Dai , H. Li , S. Li , X. Shao , and X. Cheng , “Research Advances in Chikungunya Virus: Epidemiology, Pathogenesis, and Control,” One Health 22 (2026): 101413. 10.1016/j.onehlt.2026.101413 PMC13098495 42022458

[471] J. Y. Chang , C. Balch , and H. S. Oh , “Toward the Eradication of Herpes Simplex Virus: Vaccination and Beyond,” Viruses 16, no. 9 (2024): 1476. 10.3390/v16091476 PMC11437400 39339952

[472] Y. Sun , E. Kim , C. L. Kong , B. F. Arnold , T. C. Porco , and N. R. Acharya , “Effectiveness of the Recombinant Zoster Vaccine in Adults Aged 50 and Older in the United States: A Claims‐Based Cohort Study,” Clinical Infectious Diseases 73, no. 6 (2021): 949–956. 10.1093/cid/ciab121 PMC8442779 33580245

[473] S. J. Wu , D. O. Villarreal , D. J. Shedlock , and D. B. Weiner , “Synthetic DNA Approach to Cytomegalovirus Vaccine/Immune Therapy,” Advances in Experimental Medicine and Biology 848 (2015): 131–148. 10.1007/978-1-4939-2432-5_7 PMC4573556 25757619

[474] M. T. Xu , A. Wright , A. Mah , et al., “Real‐World Use of Letermovir and Maribavir for CMV Prophylaxis and Treatment in Solid Organ Transplant Recipients: Clinical Outcomes and Resistance Patterns From a Dual‐Center Cohort,” Pharmacotherapy: The Journal of Human Pharmacology and Drug Therapy 45, no. 12 (2025): 815–824. 10.1002/phar.70086 PMC12728795 41439370

[475] A. Mancini , A. M. Inchingolo , G. Marinelli , et al., “Topical and Systemic Therapeutic Approaches in the Treatment of Oral Herpes Simplex Virus Infection: A Systematic Review,” International Journal of Molecular Sciences 26, no. 17 (2025): 8490. 10.3390/ijms26178490 PMC12428954 40943411

[476] G. A. Papanicolaou , R. K. Avery , K. Workowski , et al., “Pritelivir Demonstrated Superior Efficacy Compared to Investigator's Choice Treatment for Refractory Herpes Simplex Virus Infections in Immunocompromised Patients: PRIOH‐1, Phase 3 Safety and Efficacy,” Transplantation and Cellular Therapy 32, no. 3, Supplement (2026): S18–S19.

[477] R. Tayyar and D. Ho , “Herpes Simplex Virus and Varicella Zoster Virus Infections in Cancer Patients,” Viruses 15, no. 2 (2023): 439. 10.3390/v15020439 PMC9961783 36851652

[478] V. Sharma , S. Kaushik , R. Kumar , J. P. Yadav , and S. Kaushik , “Emerging Trends of Nipah Virus: A Review,” Reviews in Medical Virology 29, no. 1 (2019): e2010. 10.1002/rmv.2010 PMC7169151 30251294

[479] C. B. Levine , L. M. Sauer , S. L. F. McLellan , et al., “Nipah Virus: A Summary for Clinicians,” International Journal of Emergency Medicine 18, no. 1 (2025): 126. 10.1186/s12245-025-00916-1 PMC12239417 40634915

[480] C. Degeling , G. L. Gilbert , E. Annand , et al., “Managing the Risk of Hendra Virus Spillover in Australia Using Ecological Approaches: A Report on Three Community Juries,” PLoS ONE 13, no. 12 (2018): e0209798. 10.1371/journal.pone.0209798 PMC6312203 30596719

[481] G. Sulis , A. Peebles , and N. E. Basta , “Lassa Fever Vaccine Candidates: A Scoping Review of Vaccine Clinical Trials,” Tropical Medicine & International Health 28, no. 6 (2023): 420–431. 10.1111/tmi.13876 PMC10247453 37095630

[482] E. G. Playford , T. Munro , S. M. Mahler , et al., “Safety, Tolerability, Pharmacokinetics, and Immunogenicity of a Human Monoclonal Antibody Targeting the G Glycoprotein of Henipaviruses in Healthy Adults: A First‐in‐Human, Randomised, Controlled, Phase 1 Study,” The Lancet Infectious Diseases 20, no. 4 (2020): 445–454. 10.1016/S1473-3099(19)30634-6 32027842

[483] H.‐Y. Cheng , C. E. French , A. P. Salam , et al., “Lack of Evidence for Ribavirin Treatment of Lassa Fever in Systematic Review of Published and Unpublished Studies1,” Emerging Infectious Diseases 28, no. 8 (2022): 1559–1568. 10.3201/eid2808.211787 PMC9328902 35876478

[484] X. Wang and H. Xu , “Residual Proviral Reservoirs: A High Risk for HIV Persistence and Driving Forces for Viral Rebound After Analytical Treatment Interruption,” Viruses 13, no. 2 (2021): 335. 10.3390/v13020335 PMC7926539 33670027

[485] R. Bobadilla , F. MacLean , S. Dave , J. T. Blackard , and S. Gianella , “Hepatic Viral Reservoirs in Concurrent HIV and HBV: From Mechanistic Insight to Integrated Cure Strategies,” Infection and Drug Resistance 19 (2026): 1–21. 10.2147/IDR.S576715 PMC12978007 41821922

[486] E. P. Thi , X. Ye , N. M. Snead , et al., “Control of Hepatitis B Virus With Imdusiran, a Small Interfering RNA Therapeutic,” ACS Infectious Diseases 10, no. 10 (2024): 3640–3649. 10.1021/acsinfecdis.4c00514 39306863

[487] M.‐F. Yuen , Y.‐S. Lim , K. T. Yoon , et al., “VIR‐2218 (Elebsiran) Plus Pegylated Interferon‐ALFA‐2a in Participants With Chronic Hepatitis B Virus Infection: A Phase 2 Study,” The Lancet Gastroenterology & Hepatology 9, no. 12 (2024): 1121–1132. 10.1016/S2468-1253(24)00237-1 39389081

[488] J. Dunning , F. Sahr , A. Rojek , et al., “Experimental Treatment of Ebola Virus Disease With TKM‐130803: A Single‐Arm Phase 2 Clinical Trial,” PLoS medicine 13, no. 4 (2016): e1001997. 10.1371/journal.pmed.1001997 PMC4836798 27093560

[489] Y. Zhang , J. Sun , J. Zheng , et al., “Mosaic RBD Nanoparticles Elicit Protective Immunity Against Multiple Human Coronaviruses in Animal Models,” Advanced science (Weinheim, Baden‐Wurttemberg, Germany) 11, no. 9 (2024): e2303366. 10.1002/advs.202303366 PMC10916629 38105421

[490] C. Rydyznski Moderbacher , C. Kim , J. Mateus , et al., “NVX‐CoV2373 Vaccination Induces Functional SARS‐CoV‐2–Specific CD4+ and CD8+ T Cell Responses,” Journal of Clinical Investigation 132, no. 19 (2022): e160898. 10.1172/JCI160898 PMC9525112 35943810

[491] A. A. Cohen , J. R. Keeffe , L. Manasyan , et al., “mRNA Delivery of Mosaic‐8 Pan‐Sarbecovirus RBD Vaccines Elicits Distinct Antibody Epitope Signatures,” Cell Reports 45, no. 5 (2026): 117335. 10.1016/j.celrep.2026.117335 PMC13286029 42068547

[492] X. Gao , X. Wang , S. Li , M. Saif Ur Rahman , S. Xu , and Y. Liu , “Nanovaccines for Advancing Long‐Lasting Immunity Against Infectious Diseases,” ACS Nano 17, no. 24 (2023): 24514–24538. 10.1021/acsnano.3c07741 38055649

[493] A. Livieratos , G. C. Kagadis , C. Gogos , and K. Akinosoglou , “Ai Methods Tailored to Influenza, RSV, HIV, and SARS‐CoV‐2: A Focused Review,” Pathogens 14, no. 8 (2025): 748. 10.3390/pathogens14080748 PMC12389194 40872258

[494] B. M. Giles and T. M. Ross , “A Computationally Optimized Broadly Reactive Antigen (Cobra) Based H5N1 VLP Vaccine Elicits Broadly Reactive Antibodies in Mice and Ferrets,” Vaccine 29, no. 16 (2011): 3043–3054. 10.1016/j.vaccine.2011.01.100 PMC3090662 21320540

[495] N. Youssef , S. Gurev , F. Ghantous , et al., “Computationally Designed Proteins Mimic Antibody Immune Evasion in Viral Evolution,” Immunity 58, no. 6 (2025): 1411–1421.e6. 10.1016/j.immuni.2025.04.015 PMC13110123 40345199

[496] W. Sun , T. Luo , W. Liu , and J. Li , “Progress in the Development of Universal Influenza Vaccines,” Viruses 12, no. 9 (2020): 1033. 10.3390/v12091033 PMC7551969 32957468

[497] M. Caskey , F. Klein , and M. C. Nussenzweig , “Broadly Neutralizing Anti‐HIV‐1 Monoclonal Antibodies in the Clinic,” Nature Medicine 25, no. 4 (2019): 547–553. 10.1038/s41591-019-0412-8 PMC7322694 30936546

[498] Y. He , J. Zhou , H. Gao , C. Liu , P. Zhan , and X. Liu , “Broad‐Spectrum Antiviral Strategy: Host‐Targeting Antivirals Against Emerging and Re‐Emerging Viruses,” European Journal of Medicinal Chemistry 265 (2024): 116069. 10.1016/j.ejmech.2023.116069 38160620

[499] J. Wagoner , S. Herring , T.‐Y. Hsiang , et al., “Combinations of Host‐ and Virus‐Targeting Antiviral Drugs Confer Synergistic Suppression of SARS‐CoV‐2,” Microbiology Spectrum 10, no. 5 (2022): e0333122. 10.1128/spectrum.03331-22 PMC9718484 36190406

[500] T. N. D. Do , R. Abdelnabi , B. Boda , S. Constant , J. Neyts , and D. Jochmans , “The Triple Combination of Remdesivir (Gs‐441524), Molnupiravir and Ribavirin Is Highly Efficient in Inhibiting Coronavirus Replication in Human Nasal Airway Epithelial Cell Cultures and in a Hamster Infection Model,” Antiviral Research 231 (2024): 105994. 10.1016/j.antiviral.2024.105994 PMC11560660 39237005

[501] F. Zhu , S. Huang , X. Liu , et al., “Safety and Efficacy of the Intranasal Spray SARS‐CoV‐2 Vaccine dNS1‐RBD: A Multicentre, Randomised, Double‐Blind, Placebo‐Controlled, Phase 3 Trial,” The Lancet Respiratory Medicine 11, no. 12 (2023): 1075–1088. 10.1016/S2213-2600(23)00349-1 PMC10682370 37979588

[502] Y. Hou , J. Li , and Y. Wu , “Modulation of Oral Vaccine Efficacy by the Gut Microbiota,” npj Vaccines 10, no. 1 (2025): 179. 10.1038/s41541-025-01240-8 PMC12317090 40750603

[503] J. J. Norman , J. M. Arya , M. A. McClain , P. M. Frew , M. I. Meltzer , and M. R. Prausnitz , “Microneedle Patches: Usability and Acceptability for Self‐Vaccination Against Influenza,” Vaccine 32, no. 16 (2014): 1856–1862. 10.1016/j.vaccine.2014.01.076 PMC3979961 24530146

[504] G. Perego , G. P. Vigezzi , G. Cocciolo , et al., “Safety and Efficacy of Spray Intranasal Live Attenuated Influenza Vaccine: Systematic Review and Meta‐Analysis,” Vaccines 9, no. 9 (2021): 998. 10.3390/vaccines9090998 PMC8472940 34579235

[505] M. T. Yeh , E. Bujaki , P. T. Dolan , et al., “Engineering the Live‐Attenuated Polio Vaccine to Prevent Reversion to Virulence,” Cell Host & Microbe 27, no. 5 (2020): 736–751.e8. 10.1016/j.chom.2020.04.003 PMC7566161 32330425

[506] R. Okuyama , “mRNA and Adenoviral Vector Vaccine Platforms Utilized in Covid‐19 Vaccines: Technologies, Ecosystem, and Future Directions,” Vaccines 11, no. 12 (2023): 1737. 10.3390/vaccines11121737 PMC10748114 38140142

[507] A. Fournillier , L. Frelin , E. Jacquier , et al., “A Heterologous Prime/Boost Vaccination Strategy Enhances the Immunogenicity of Therapeutic Vaccines for Hepatitis C Virus,” The Journal of Infectious Diseases 208, no. 6 (2013): 1008–1019. 10.1093/infdis/jit267 PMC3749006 23776192

[508] D. Ward , L. Pattarapreeyakul , R. Pitaksalee , et al., “Serological Insights From SARS‐CoV‐2 Heterologous Prime and Boost Responses in Thailand,” Scientific Reports 15, no. 1 (2025): 1519. 10.1038/s41598-024-84392-2 PMC11718049 39789037

[509] A. Elkashif , M. Alhashimi , E. E. Sayedahmed , S. Sambhara , and S. K. Mittal , “Adenoviral Vector‐Based Platforms for Developing Effective Vaccines to Combat Respiratory Viral Infections,” Clinical & Translational Immunology 10, no. 10 (2021): e1345. 10.1002/cti2.1345 PMC8510854 34667600

[510] S. Beitari , G. Agbayani , M. Hewitt , et al., “Effectiveness of VSV Vectored SARS‐CoV‐2 Spike When Administered Through Intranasal, Intramuscular or a Combination of Both,” Scientific Reports 13, no. 1 (2023): 21390. 10.1038/s41598-023-48397-7 PMC10695950 38049498

